# Distinct and Modular Organization of Protein Interacting Sites in Long Non-coding RNAs

**DOI:** 10.3389/fmolb.2018.00027

**Published:** 2018-04-04

**Authors:** Saakshi Jalali, Shrey Gandhi, Vinod Scaria

**Affiliations:** ^1^GN Ramachandran Knowledge Center for Genome Informatics, CSIR Institute of Genomics and Integrative Biology, New Delhi, India; ^2^CSIR Institute of Genomics and Integrative Biology, Academy of Scientific and Innovative Research, New Delhi, India

**Keywords:** long non-coding RNAs, RNA binding proteins, protein-lncRNA interactions, Argonaute (ago), MALAT1

## Abstract

**Background:** Long non-coding RNAs (lncRNAs), are being reported to be extensively involved in diverse regulatory roles and have exhibited numerous disease associations. LncRNAs modulate their function through interaction with other biomolecules in the cell including DNA, RNA, and proteins. The availability of genome-scale experimental datasets of RNA binding proteins (RBP) motivated us to understand the role of lncRNAs in terms of its interactions with these proteins. In the current report, we demonstrate a comprehensive study of interactions between RBP and lncRNAs at a transcriptome scale through extensive analysis of the crosslinking and immunoprecipitation (CLIP) experimental datasets available for 70 RNA binding proteins.

**Results:** Our analysis suggests that density of interaction sites for these proteins was significantly higher for specific sub-classes of lncRNAs when compared to protein-coding transcripts. We also observe a positional preference of these RBPs across lncRNA and protein coding transcripts in addition to a significant co-occurrence of RBPs having similar functions, suggesting a modular organization of these elements across lncRNAs.

**Conclusion:** The significant enrichment of RBP sites across some lncRNA classes is suggestive that these interactions might be important in understanding the functional role of lncRNA. We observed a significant enrichment of RBPs which are involved in functional roles such as silencing, splicing, mRNA processing, and transport, indicating the potential participation of lncRNAs in such processes.

## Background

The recent years have seen the discovery of a large number of novel transcripts which belong to the long non-coding RNA (lncRNA) class in humans and other model organisms (Pauli et al., 2012). This has been largely contributed by the availability of high-throughput methodologies for transcriptome annotation, including tiling microarrays (Hafner et al., 2010a; Furey, 2012) and deep sequencing (Roberts et al., 2011). The recent genome-wide analyzes of lncRNA genes in Humans have annotated over 83,215 transcripts from 32,446 lncRNAs genes (Derrien et al., 2012; Harrow et al., 2012). The lncRNA superset presently includes a number of sub-classes which include 3 prime overlapping ncRNA, antisense, bidirectional promoter lncRNA, lincRNA, macro lncRNA, miscRNA, non-coding, processed transcripts, pseudogene, retained intron, sense intronic, sense overlapping, and TEC. By definition lncRNAs encompass all transcripts > 200 nucleotides in length and no ORF coding for more than 30 amino acids (Mercer et al., 2009). The biogenesis and regulation of lncRNAs have not been studied in great detail, though it is believed that they are transcribed majorly by Polymerase II and are capped and polyadenylated (Goodrich and Kugel, 2006; Gibb et al., 2011). One particular class of lncRNAs, the large intergenic non-coding RNA has been primarily discovered through their association with epigenetic marks in the genome (Cabili et al., 2011; Cao, 2014). We have recently shown extensive similarities and specific dissimilarities in epigenetic regulation of lncRNAs in comparison to protein-coding genes (Sati et al., 2012). The precise biological function of many of the lncRNAs are not known, though a handful of the candidates have been recently shown to be mechanistically involved in gene regulation and associated with diseases (Wapinski and Chang, 2011). Recent reports from our group also suggest processing of a subset of lncRNAs to smaller RNAs (Jalali et al., 2012), and that a subset of lncRNAs could be potentially targeted by microRNAs (Jalali et al., 2013), thus constituting an intricate and yet poorly understood network of non-coding RNA mediated regulation.

Mechanistically, the characterization of lncRNA could be generalized as a function of its interactions with other biomolecules in the cell: DNA, RNA, protein, and small-molecules (Bhartiya et al., 2012). Current studies have showed that molecular and computational biology techniques can act as catalyst in discovering lncRNA-mediated regulation via understanding their interactions with different biomolecules (Jalali et al., 2015). Recent reports have also suggested the possibility of protein-lncRNA interactions and regulatory interactions mediated through them (Kung et al., 2013). The present understanding of protein-lncRNA interactions are limited to a handful of candidates associated with proteins involved in epigenetic modifications as in the cases of HOTAIR (Gupta et al., 2010), Anril (Kogo et al., 2011), and Xist (Arthold et al., 2011); splicing as in the case of MALAT1 (or NEAT2) (Tripathi et al., 2010) conserved nuclear ncRNA; transcriptional regulation through interaction with transcription factors as in the case of Gas5 (Kino et al., 2010) and few other candidates like Meg3 (Zhao et al., 2006), DHFR (Blume et al., 2003), and Gomafu (Sheik Mohamed et al., 2010). It has been recommended that computational methods for predicting protein-RNA interactions, though less accurate, could be potentially used to guide in experiments (Puton et al., 2012). Recently experimental methodologies to understand protein-RNA interactions on a genomic-scale, including CLIP-seq (Darnell, 2012) and variants thereof (Hafner et al., 2010a; Jain et al., 2011; Konig et al., 2011) has provided insights into the target-sites of a number of RNA binding proteins with much higher resolution (Popov and Gil, 2010). The availability of genome-scale maps of RNA binding proteins provide a novel opportunity toward understanding patterns of RNA binding proteins interaction sites in different transcript classes and derive clues on the interaction networks, regulation and functional consequences of these interactions.

Recently, Li and coworkers showed the interaction between protein and lncRNAs, in addition to their association with disease causing SNPs. They have deposited all the interaction data in form of bed files in starBase 2.0 database, the same datasets are also included in our current study (Li et al., 2014). Tartaglia and coworkers have also employed a novel algorithm catRAPID to evaluate the binding tendency of protein with RNAs (Livi et al., 2015). A similar study by Park et al. has also attempted to explore the possible functions of lncRNAs by focusing at the RBP-lncRNA interactions. LncRNAtor functionally annotates lncRNA molecules based on their expression profiles and co-expression with mRNAs. It also encompass lncRNA's interaction data with 57 RBPs for 5 organisms (Park et al., 2014).

The functional interactions of lncRNAs could be potentially summarized as the sum total of the interactions between other biomolecules independently or in context of one another. The interaction of lncRNAs with genomic DNA and its involvement in chromatin organization (Lee and Bartolomei, 2013) and with other RNA species (Salmena et al., 2011; Bhartiya et al., 2012; Jalali et al., 2015) including microRNAs (Jalali et al., 2013) has been explored at length. Though there have been a number of reports characterizing functional roles of lncRNAs through their association with proteins (Wilusz et al., 2009), no systematic analysis reports has been published on mapping or on characterizing the functional domains of lncRNAs for protein-binding sites. Our study focuses on providing a platform to explore these interactions at a larger scale using computational approaches to functionally indict the lncRNA molecules.

In the present report, we have performed a comprehensive analysis of 70 experimental RNA binding protein datasets available in the public domain. We have derived the peak information (or the most probable site of interaction between protein and RNA) for these RNA binding protein sites at a genome-scale from doRiNA (Blin et al., 2015), starBase (Yang et al., 2011; Li et al., 2014), and CLIPdb (Yang et al., 2015) and analyzed their binding sites in lncRNAs and protein coding transcripts. Our analysis suggests 6 lncRNA subtypes (viz; antisense, lincRNA, miscRNA, processed transcripts, retained intron, and sense intronic) to be largely enriched for protein-binding sites compared to other subclasses hence potentially contribute to a novel layer of regulatory interactions mediated through protein-RNA interactions in ncRNA transcripts. Our analysis shows the distribution of RBP binding sites on the lncRNA loci as opposed to only protein coding transcripts. In our study, we also reveal an interesting pattern of positional clustering of RBP target sites in lncRNAs suggesting a modular organization of regulatory sites in lncRNAs. We also propose how the functionally similar proteins co-occur in both protein coding and lncRNA transcripts. To our knowledge, this is the most comprehensive study on the comparison of lncRNA-RBP interactions as opposed to protein coding loci.

## Methods

### Long non-coding RNA datasets

We used the comprehensive compendium of lncRNAs available from GENCODE Version 24 (August 2015 freeze, GRCh38, Ensembl 83, 84) (http://www.gencodegenes.org/) (Harrow et al., 2012). The lncRNA dataset had a total of 32,446 genes encompassing 83,215 transcripts having 3,14,672 exons comprising of both Ensembl and Havana annotations. LncRNA transcripts were assigned into 13 biotypes, viz, 3prime overlapping ncRNA, antisense, bidirectional promoter lncRNA, lincRNA, macro lncRNA, miscRNA, non-coding, processed transcripts, pseudogene, retained intron, sense intronic, sense overlapping, and TEC. We also extracted the 19,655 protein coding genes with 79,930 transcripts and their 7,11,466 exons.

### Genome scale datasets for protein-RNA interactions

We have compiled and analyzed the protein-RNA interaction datasets from public domain for 70 unique proteins derived from 51 publications across 3 databases (detailed in **Table 2**). The RBP binding sites were downloaded from 3 databases namely: starBase v2.0 (Yang et al., 2011; Li et al., 2014), doRiNA 2.0 (Blin et al., 2015), and ClipDB v1.0 (last updated: April, 2015) (Yang et al., 2015). The ClipDB database consisted of datasets analyzed using 4 different softwares PARalyzer (Corcoran et al., 2011), CIMS (Crosslinking induced mutation site) (Moore et al., 2014), CITS (Weyn-Vanhentenryck et al., 2014), and Piranha (Uren et al., 2012).

These datasets comprise of positions of interaction of RNA binding protein and RNA target sites derived after PAR-CLIP (Photoactivatable Ribonucleoside Enhanced Crosslinking and Immunoprecipitation), HITS-CLIP-seq (High Throughput sequencing of RNA isolated by crosslinking immunoprecipitation), RIP-seq (RNA immunoprecipitation), iCLIP (individual nucleotide resolution crosslinking and immunoprecipitation), PAR-iCLIP (Photoactivatable Ribonucleoside Enhanced individual nucleotide resolution crosslinking and immunoprecipitation) and CLASH (cross-linking ligation and sequencing of hybrids) followed by sequencing of the pull-down fraction of RNA. The sequenced RNA is further used to identify exact or probable binding site using various bioinformatic approaches. In case of ClipDB, the peak calling and identification were done using PARalyzer, CIMS, Piranha, and CITS software tools. Hence, we stored each of files derived from all databases in form of peaks as separate files for downstream analysis. Details of all the techniques and methodologies used to process the data used in our analysis is given in Table 1.

**Table 1:** The summary of total datasets examined in our study for analysis.

**Database**	**Technique used**	**Software to predict binding sites**	**Total datasets**	**No of unique RBP**
starBase	CLASH, HITS-CLIP, iCLIP, PAR-CLIP	Ago PAR-CLIP raw data were reanalyzed using PARalyzer v1.1, other CLIP-identified binding sites clusters/peaks were used directly from their respective publications	86	38
CLIPdb	HITS-CLIP, iCLIP, PAR-CLIP, PAR-iCLIP	PARalyzer, CIMS, Piranha, CITS	430	59
doRiNA	iCLIP, HITS-CLIP,CLIP-Seq, PAR-CLIP	Binding sites directly adopted from their respective publications	79	37

All these RNA binding sites were liftover to hg38/GRCh38 assembly using the CrossMap-0.2.2 tool (Zhao et al., 2014). The peak information was available for proteins as shown in Supplementary Tables 1A,B. In total, we considered 7 datasets for our study, namely: (1) starBase; (2) doRiNA; (3) Clipdb-PARalyzer; (4) CLIPdb-CIMS; (5) CLIPdb-CITS; (6) CLIPdb-Piranha-stranded); and (7) CLIPdb-Piranha-non-stranded.

### Mapping of RNA binding protein interaction sites

The peaks of the RNA binding protein interaction sites were mapped to the lncRNA exons using bespoken perl script and BEDtools (v2.17.0) (Quinlan and Hall, 2010). The most probable site of interaction (or the peaks) between protein and RNA were derived from datasets taken from doRiNA, starBase, and CLIPdb databases which were processed through standard computational pipelines (as listed in Table 2), offering an easy comparability at the analysis point of view. Further, we tried to analyze the binding sites in each of the individual lncRNA subclasses as defined by GENCODE annotations (i.e., 3 prime overlapping ncRNA, antisense, bidirectional promoter lncRNA, lincRNA, macro lncRNA, miscRNA, non-coding, processed transcripts, pseudogene, retained intron, sense intronic, sense overlapping, and TEC). Similarly, we also plotted the distribution of the binding sites across the protein-coding exons derived from the GENCODE v24 annotation file.

**Table 2:** List of the 70 RNA binding proteins derived from the respective databases.

**S. no**.	**Name**	**Full name**	**doRiNA**	**starBase**	**Clipdb**	**Publications**
1	AGO1	Argonaute RISC catalytic component 1	✓	✓	✓	Hafner et al., 2010b; Helwak et al., 2013; Memczak et al., 2013
2	AGO2	Argonaute RISC catalytic component 2	✓	✓	✓	Hafner et al., 2010b; Gottwein et al., 2011; Kishore et al., 2011; Lipchina et al., 2011; Haecker et al., 2012; Riley et al., 2012; Skalsky et al., 2012; Karginov and Hannon, 2013; Memczak et al., 2013; Xue et al., 2013
3	AGO3	Argonaute RISC catalytic component 3	✓	✓	✓	Hafner et al., 2010b
4	AGO4	Argonaute RISC catalytic component 4	✓	✓	✓	Hafner et al., 2010b
5	ALKBH5	Alpha-Ketoglutarate-Dependent Dioxygenase AlkB Homolog 5	✓	✓	✓	Baltz et al., 2012
6	ATXN2	Ataxin 2	✓		✓	Yokoshi et al., 2014
7	C17ORF85/NCBP3	Chromosome 17 Open Reading Frame 85/Nuclear Cap Binding Subunit 3	✓	✓	✓	Baltz et al., 2012
8	C22ORF28/RTCB	Chromosome 22 Open Reading Frame 28 /RNA 2,3-Cyclic Phosphate And 5-OH Ligase	✓	✓		Baltz et al., 2012
9	CAPRIN1	Cytoplasmic Activation- And Proliferation-Associated Protein 1	✓	✓	✓	Baltz et al., 2012
10	CPSF1	Cleavage And Polyadenylation Specific Factor 1			✓	Martin et al., 2012
11	CPSF2	Cleavage And Polyadenylation Specific Factor 2			✓	Martin et al., 2012
12	CPSF3	Cleavage And Polyadenylation Specific Factor 3			✓	Martin et al., 2012
13	CPSF4	Cleavage And Polyadenylation Specific Factor 4			✓	Martin et al., 2012
14	CPSF6	Cleavage And Polyadenylation Specific Factor 6			✓	Martin et al., 2012
15	CPSF7	Cleavage And Polyadenylation Specific Factor 7			✓	Martin et al., 2012
16	CSTF2	Cleavage Stimulation Factor Subunit 2			✓	Martin et al., 2012; Yao et al., 2012
17	CSTF2T	Cleavage Stimulation Factor Subunit 2, Tau Variant			✓	Martin et al., 2012
18	DGCR8	DiGeorge Syndrome Critical Region 8	✓	✓	✓	Macias et al., 2012
19	EIF4A3	Eukaryotic Translation Initiation Factor 4A3	✓	✓	✓	Saulière et al., 2012
20	ELAVL1/HUR	Embryonic Lethal Abnormal Vision Drosophila)-Like binding protein 1/Human Antigen R	✓	✓	✓	Lebedeva et al., 2011; Mukherjee, 2011; Friedersdorf and Keene, 2014
21	EWSR1	Ewing Sarcoma Breakpoint Region 1	✓	✓	✓	Hoell et al., 2011; Paronetto et al., 2014
22	EZH2	Enhancer Of Zeste 2 Polycomb Repressive Complex 2 Subunit			✓	Kaneko et al., 2013
23	FBL	Fibrillarin			✓	Kishore et al., 2013
24	FIP1L1	Factor Interacting With PAPOLA And CPSF1			✓	Martin et al., 2012
25	FMR1	Fragile X Mental Retardation 1	✓	✓	✓	Ascano et al., 2012
26	FOX2	RNA binding protein fox-1 homolog 2	✓			Yeo et al., 2009
27	FUS	Fused in Sarcoma	✓	✓	✓	Hoell et al., 2011; Lagier-Tourenne et al., 2012; Nakaya et al., 2013; Yokoshi et al., 2014
28	FXR1	Fragile X Mental Retardation, Autosomal Homolog 1	✓	✓	✓	Ascano et al., 2012
29	FXR2	Fragile X Mental Retardation Autosomal Homolog 2	✓	✓	✓	Ascano et al., 2012
30	HNRNPA1	Heterogeneous Nuclear Ribonucleoprotein A1			✓	Huelga et al., 2012
31	HNRNPA2B1	Heterogeneous Nuclear Ribonucleoprotein A2/B1			✓	Huelga et al., 2012
32	HNRNPC	Heterogeneous Nuclear Ribonucleoprotein C (C1/C2)	✓	✓	✓	Zarnack et al., 2010, 2013
33	HNRNPD	Heterogeneous Nuclear Ribonucleoprotein D			✓	Yoon et al., 2014
34	HNRNPF	Heterogeneous Nuclear Ribonucleoprotein F			✓	Huelga et al., 2012
35	HNRNPH	Heterogeneous Nuclear Ribonucleoprotein H1			✓	Katz et al., 2010
36	HNRNPL	Heterogeneous Nuclear Ribonucleoprotein L	✓		✓	Shankarling et al., 2014
37	HNRNPM	Heterogeneous Nuclear Ribonucleoprotein M			✓	Huelga et al., 2012
38	HNRNPU	Heterogeneous Nuclear Ribonucleoprotein U (Scaffold Attachment Factor A)			✓	Huelga et al., 2012; Xiao et al., 2012
39	IGF2BP1	Insulin Like Growth Factor 2 MRNA Binding Protein 1	✓	✓	✓	Hafner et al., 2010b
40	IGF2BP2	Insulin Like Growth Factor 2 MRNA Binding Protein 2	✓	✓	✓	Hafner et al., 2010b
41	IGF2BP3	Insulin Like Growth Factor 2 MRNA Binding Protein 3	✓	✓	✓	Hafner et al., 2010b
42	LIN28A	Lin-28 Homolog A	✓	✓	✓	Wilbert et al., 2012; Hafner et al., 2013
43	LIN28B	Lin-28 Homolog B	✓	✓	✓	Graf et al., 2013; Hafner et al., 2013
44	METTL3	Methyltransferase-Like Protein 3	✓	✓	✓	Ping et al., 2014
45	MOV10	Moloney Leukemia Virus 10 RISC Complex RNA Helicase	✓	✓	✓	Sievers et al., 2012
46	NOP56	Nucleolar Protein 5A			✓	Kishore et al., 2013
47	NOP58	Nucleolar Protein 5			✓	Kishore et al., 2013
48	NUDT21	Nudix (Nucleoside Diphosphate Linked Moiety X)-Type Motif 21			✓	Martin et al., 2012
49	PTBP1	Polypyrimidine Tract Binding Protein 1		✓	✓	Xue et al., 2013; Raj et al., 2014
50	PTBP1 and PTBP2	Polypyrimidine Tract Binding Protein 1 and Polypyrimidine Tract Binding Protein 2			✓	Xue et al., 2009
51	PUM2	Pumilio RNA Binding Family Member 2	✓	✓	✓	Hafner et al., 2010b
52	QKI	Quaking homolog KH domain RNA binding	✓	✓	✓	Hafner et al., 2010b
53	RBM10	RNA Binding Motif Protein 10	✓			Wang et al., 2013
54	RBPMS	RNA Binding Protein With Multiple Splicing	✓			Farazi et al., 2014
55	RTCB	RNA 2′,3′-Cyclic Phosphate And 5'-OH Ligase			✓	Baltz et al., 2012
56	SFRS1	Splicing factor arginine/serine-rich 1	✓	✓		Sanford et al., 2009
57	SRRM4	Serine/Arginine Repetitive Matrix 4			✓	Raj et al., 2014
58	TAF15	TATA-Box Binding Protein Associated Factor 15	✓	✓	✓	Hoell et al., 2011; Ibrahim et al., 2013
59	TARDBP/TDP-43	Transactive response DNA binding protein 43 kDa	✓	✓	✓	Tollervey et al., 2011; Yokoshi et al., 2014
60	TIA1	T-Cell-Restricted Intracellular Antigen-1	✓	✓	✓	Ule et al., 2010
61	TIAL1	TIA-1-Related Protein	✓	✓		Ule et al., 2010
62	TNRC6A	Trinucleotide repeat-containing gene 6A		✓	✓	Hafner et al., 2010b
63	TNRC6B	Trinucleotide repeat-containing gene 6B		✓	✓	Hafner et al., 2010b
64	TNRC6C	Trinucleotide repeat-containing gene 6C		✓	✓	Hafner et al., 2010b
65	U2AF65	U2 Small Nuclear Ribonucleoprotein Auxiliary Factor (65kD)		✓		Zarnack et al., 2013
66	UPF1	Up-Frameshift Suppressor 1 Homolog		✓		Zünd et al., 2013
67	WDR33	WD Repeat Domain 33			✓	Schönemann et al., 2014
68	WTAP	Wilms Tumor 1 Associated Protein	✓			Ping et al., 2014
69	YTHDF2	YTH N6-Methyladenosine RNA Binding Protein 2			✓	Wang et al., 2014
70	ZC3H7B	Zinc Finger CCCH-Type Containing 7B	✓	✓	✓	Baltz et al., 2012

We further tested the significance of binding frequency for each of the lncRNA biotype when compared to the protein coding transcripts. The normalized frequency of binding was calculated by dividing the unique number of RBP peaks mapped from each dataset by unique number of bases of lncRNA/protein coding/random transcripts per kb. Statistical unpaired *t*-test was applied using R (version 3.1.3) (R Core Team, 2015) script, to test if any of the lncRNA biotypes had significantly higher RBP binding frequency as compared to the protein coding transcripts.

### Combinatorial patterns for RNA binding protein interaction sites in lncRNAs

We explored the possibility of positional clustering of RNA binding protein interaction sites across the lncRNA and protein coding transcripts. For this, we calculated the co-occurrence binding frequencies for each of the 70 RBPs from the six datasets for each of the lncRNAs and protein coding transcripts in the annotation list. For this analysis we did not consider the CLIPdb-Piranha-non-stranded) dataset due to lack of strand orientation information. Bespoke shell scripts were used to identify RBP sites which co-occurred with each other and were therefore clubbed together.

The coordinates for each RBP peak dataset were intersected separately with both the lncRNA and protein coding exons using BEDtools. These intersecting coordinates were then used to calculate the number of bases which were shared between each of the protein datasets to examine their co-occurrence. The values were further normalized by dividing it with the total number of unique bases of individual RBP datasets which were intersecting with lncRNA and protein coding exons. The mapping percentage in protein coding transcripts provided the baseline for co-occurrence frequency of the binding sites. These co-occurrence frequencies were calculated independently for all the RBP across six datasets.

### Positional preference of RNA binding protein interaction sites in lncRNAs

We also examined the positional preference of the RNA binding protein interaction sites across the length of lncRNA transcript. As the length of the transcripts varied considerably in our analysis therefore, we briefly define the length of the transcripts as divided into three equal parts. The length of long non-coding transcripts were normalized to 100 nucleotides and arbitrary divided into three equal parts viz., 5 prime end, the middle region, and 3 prime end for comparisons. The notation 5 prime, middle region, and 3 prime denote the positions of the three equal fragments and have no bearing with 5 prime and 3 prime UTRs. Except for datasets analyzed using Piranha, which did not have strand information of the called RBP peaks, all other datasets were used to check for their positional preference. The unique number of bases intersecting with each of the three lncRNA segments was calculated for each dataset. These were further normalized by dividing these values with the unique number of bases in the respective lncRNA segment. Percentage preference was calculated for each segment and the positional location of RNA protein-binding sites were enumerated and plotted as heatmaps.

Additionally, we also plotted the counts of the RNA binding protein interactions sites in protein coding transcripts derived from GENCODE annotation file and the mappings were divided into 3 regions: 5 prime UTR, coding exons, and 3 prime UTR of the coding genes. The CLIPdb-Piranha-non-stranded dataset were not used for the analysis due to the lack of strand information of the peaks.

## Results

### Analysis of mapping of RNA binding proteins datasets

We analyzed publicly available datasets for 70 RNA binding proteins derived from seven datasets encompassing five technologies viz. PAR-CLIP, HITS-CLIP, iCLIP, RIP-seq, and CLASH. The experimental datasets were downloaded for RNA binding proteins from three databases (details in Table 1). The experiments briefly included high-throughput genome-scale analysis of RNA protein interactions through pull down and sequencing. The derived data in form of interaction sites (or peaks) which were pre-processed using different computational pipelines including PARalyzer, CIMS, Piranha, and CITS for each of the proteins and were mapped onto the hg38 build of the Human reference genome. The total number of peaks mapping to the genome for respective datasets corresponding to each RNA binding protein has been detailed in Supplementary Tables 1A,B. Each of the dataset was kept as a separate file even if the name of the RNA binding protein was same. This was followed to maintain the identity of each dataset as there were differences in number of peaks for same proteins across different databases which could be attributed to the different experimental protocols used for processing including difference in cell lines, conditions or end points, or downstream computational processing. As same protein was present in more than one dataset, we did not group them as one because different databases had differences in the number and position of peaks owing to the differences in the peak calling softwares and computational pipelines adopted by the users. Nevertheless, the differences in the global frequencies have not been influenced by these.

### Comparison of RNA binding protein interaction sites within lncRNAs and protein coding genes

We compared the interaction sites for each of the RNA binding proteins in lncRNAs as well as protein-coding transcripts. Toward this end, we used the transcript annotations as provided by GENCODE V24 (Harrow et al., 2012) for protein-coding transcripts and lncRNAs. In total the dataset comprised of 79,930 protein-coding transcripts from 19,655 genes and 83,215 lncRNA transcripts arising out of 32,446 genic loci. We analyzed the distribution of RNA binding protein interaction sites across lncRNAs and protein coding transcripts.

All proteins showed distinct frequency distribution across both protein-coding and long non-coding transcripts. In general, RBP binding was higher in protein coding transcripts when compared to long non-coding transcripts. But when we looked closely, few of RBPs showed higher enrichment for lncRNA subclass when compared to protein coding transcripts. We tested the significance of the enrichment of RBP sites across lncRNA subtypes as opposed to protein coding transcripts using paired *t*-test. We observed that six of the biotypes including antisense, lincRNA, miscRNA, processed transcripts, retained intron, and sense intronic were more enriched (*p*-value ≤ 0.05) for RBP sites as opposed to protein coding transcripts in some or the other RBP dataset.

**Figure 1:**
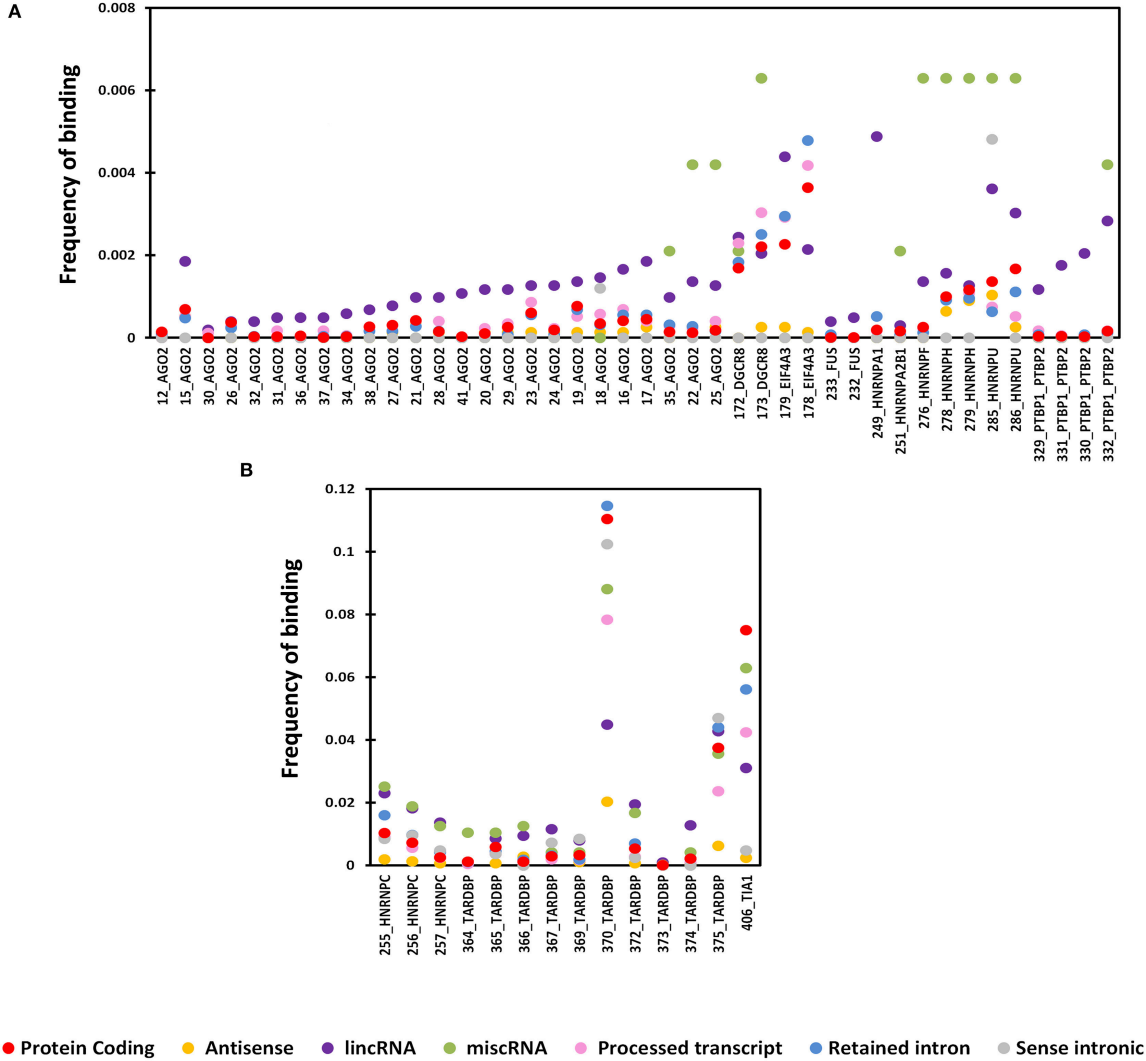
Distribution of RNA binding proteins from CLIPdb-CIMS across 6 biotypes of lncRNA genes and protein-coding genes. X-axis of the graph shows the distribution of RNA binding protein interaction sites in subclasses of lncRNAs and protein coding genes frequency of binding sites. The Y-axis represents the normalized frequency of RBP binding, which was calculated as Unique No. of RBP peaks mapped/Unique No. of Exonic bases/1000. Different ranges of frequency are plotted in **A** (0-0.008) and **B** (0-0.12).

We plotted the binding frequencies of RBPs in lncRNAs and protein coding transcripts for each of the seven datasets as separate graphs. Those datasets and biotypes which had a significantly higher binding for RBPs have been plotted (Figure 1, Supplementary Figures 1, 2). The RBP binding frequency for CLIPdb-CIMS dataset was significantly higher in lincRNA class when compared to protein coding transcripts for all proteins, while HNRNP (F, H, and U) protein had consistent enrichment for miscRNA class (Figure 1). HNRNP complexes help in processing of pre-mRNAs into functional, translatable mRNAs in the cytoplasm. AGO group from CLIPdb-Piranha-non-stranded dataset were mostly enriched for miscRNA, sense intronic, and lincRNA class compared to protein coding transcript while most of proteins showed enrichment for miscRNA and lincRNA classes (Supplementary Figure 1). In Supplementary Figure 1B, we observed miscRNA and lincRNA class to be mostly enriched for most of proteins including AGO proteins, CSTF2 in sense intronic and DGCR8 in retained intron class. AGO2 protein is an important part of RNA-induced silencing complex (RISC) and is required for RNA-mediated gene silencing (RNAi). CSTF2 plays role in polyadenylation and 3'-end cleavage of mammalian pre-mRNAs. DGCR8 is a component of the microprocessor complex that acts as a RNA- and heme-binding protein that is involved in the initial step of microRNA (miRNA) biogenesis. For the starBase, CLIPdb-CITS, doRiNA, Clipdb-PARalyzer datasets RBPs showed higher frequency distribution for lncRNAs (miscRNA, retained intron processed transcript) compared to protein coding transcripts (Supplementary Figures 2A–D), ATXN2 protein from Supplementary Figure 2D had a comparable binding frequency in miscRNA class to protein coding transcripts. This protein is involved in EGFR trafficking, acting as negative regulator of endocytic EGFR internalization at the plasma membrane. Proteins from CLIPdb-Piranha-stranded had enrichment for miscRNA class when compared to protein coding transcripts (Supplementary Figure 2E).

We additionally chose a random set of 1,000,000 (1 million) genomic loci as a control set with an average length of 240 bases and mapped the RBP sites across this control set. The frequencies of protein binding sites across these random genomic loci, lncRNA, and protein coding transcripts of randomly chosen RBPs from each of the six datasets have been depicted in the Supplementary Figure 3, to illustrate that the frequency of protein binding sites in lncRNAs is not an arbitrary event. The observed RBP frequency was significantly lower for these random positions when compared to protein coding transcripts and lncRNAs. This clearly substantiates the fact that the observed RBP distribution frequencies are not just due to randomness but are inherently due to the class of RNA they bind.

### Combinatorial patterns for protein-binding sites in lncRNAs show similar proteins have overlapping binding sites

The seven datasets considered in this study were observed to map onto lncRNA transcripts as well as protein-coding transcripts. To understand whether they map to common subset of loci in the respective transcripts, we evaluated the positional overlaps of the binding sites for each protein from these seven datasets individually. The counts of overlaps were measured as proportion of the total number of independent occurrences of binding sites for each protein. The overlaps were counted separately for all positions in the protein coding transcripts and in lncRNAs. The mapping in protein coding transcripts served as the control set which provided a fair idea of the general overlap in the genomic scale.

**Figure 2:**
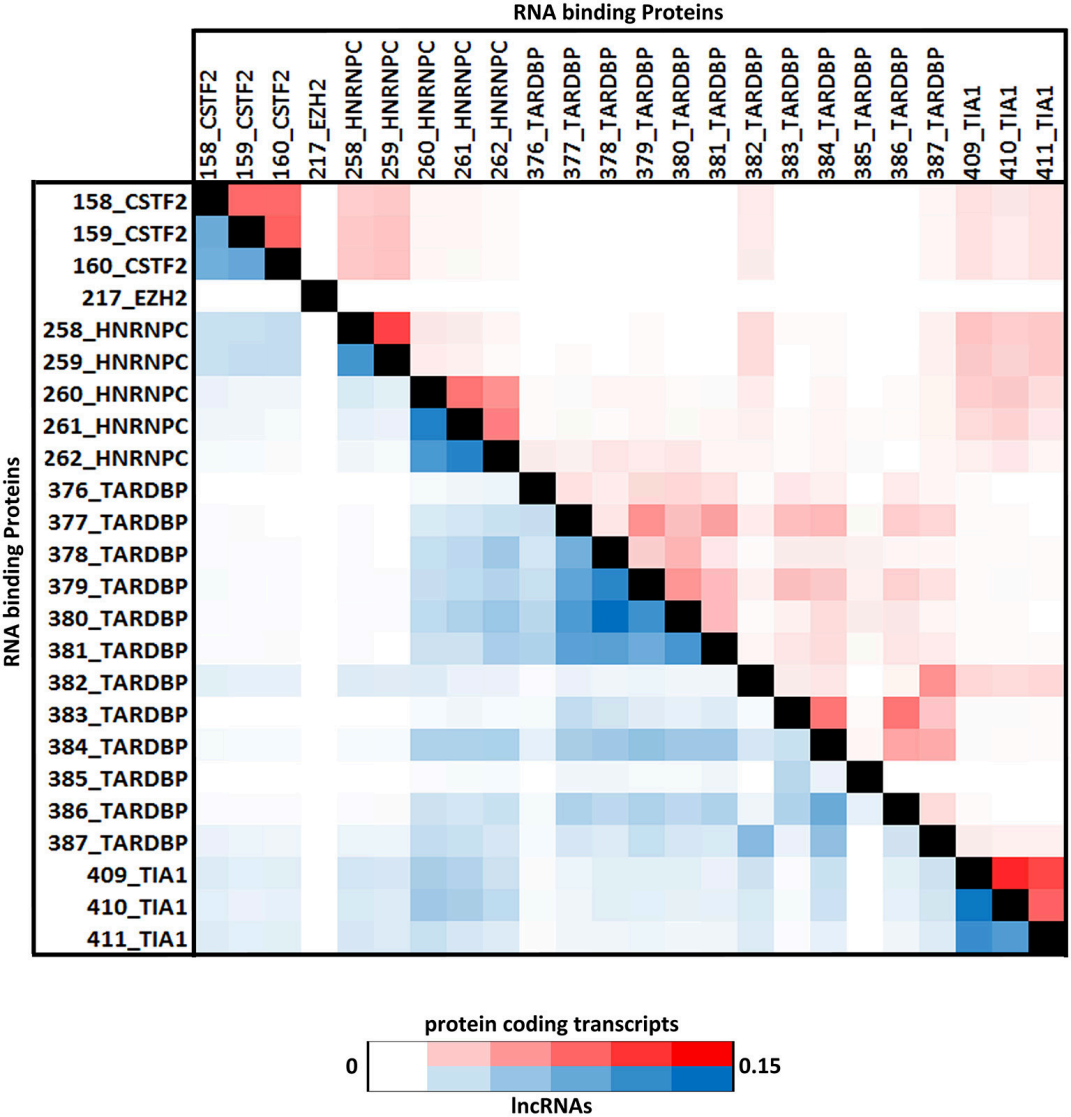
The Heatmap depicts the combinatorial patterns of clustered protein-binding sites across lncRNAs (blue in color) and protein coding transcripts (red in color) for CLIPdb-CITS RBPs. The scale here signifies the number of overlapping binding sites per total number of occurrences for the independent proteins. The diagonal blocks represent a value of 1 corresponding to the exact overlap between the individual protein datasets.

Four proteins from the CLIPdb-CITS dataset CSTF2, HNRNPC, TARDB, and TIA1 showed maximum co-occurrence with their respective set of proteins both in protein coding and lncRNAs transcripts while CSTF2, HNRNPC, and TIAL1 co-occurred with each other as well. Our analysis revealed that similar functioning proteins have significantly higher overlapping binding sites with each other, as expected, while EZH2 was an exception in this dataset (Figure 2).

Similarly, RBPs from other five datasets also showed same behavior of co-occurrence between the same set of proteins as shown in Supplementary Figures 4–8 as heatmap. ELAVL1 co-occurred with HUR proteins from doRiNA dataset with high co-occurrence binding frequency as both being the alternate name of same protein. HNRNPF co-occurred with HNRNPU; both are part of the same HNRNP complex, infact all the HNRNP proteins are related to each other.

While protein having similar function such as AGO and DGCR8 proteins were co-occurring in both the doRiNA and CLIPdb-CIMS datasets. Similarly, TNRC6 (A-C) proteins co-occurred with AGO proteins from CLIPdb-Piranha-stranded, Clipdb-PARalyzer, and starBase datasets, from previous observations it is has been seen that functionally related proteins co-occur as in case of TNRC6 with Argonautes, as they have shown to be to play important roles in microRNA mediated regulation of transcripts (Baillat and Shiekhattar, 2009; Chen et al., 2009). ATXN2 and TARDB from Clipdb-PARalyzer are known to associate in one complex depending on RNA where they bind, we observed them to co-occur in our analysis (Elden et al., 2010). From Clipdb-PARalyzer dataset CSTF2 co-occurred with CPSF proteins. Argonaute protein was observed to co-occur with FUS, HNRNP, PTBP1, and PTBP2 from CLIPdb-CIMS datasets and from literature it has been reported that all these proteins interact with each except AGO, hence we believe if other proteins co-occur then AGO should also functionally correlate with these proteins. From starBase dataset, we also observed TAF15 and FUS co-occurred. In addition, we also observed that FUS and TARDB proteins co-occurred from Clipdb-PARalyzer dataset and AGO group of proteins from CLIPdb-CIMS dataset co-occured with HNRNP2B1, HNRNPF, HNRNPM, and HNRNPU proteins. There were other proteins also which co-occurred but with low co-occurrence binding frequency. There was no stark difference in the overlaps of the binding sites between protein coding transcripts and lncRNA sites for each of the proteins considered in our analysis.

### Positional clustering of the protein-binding sites

**Figure 3:**
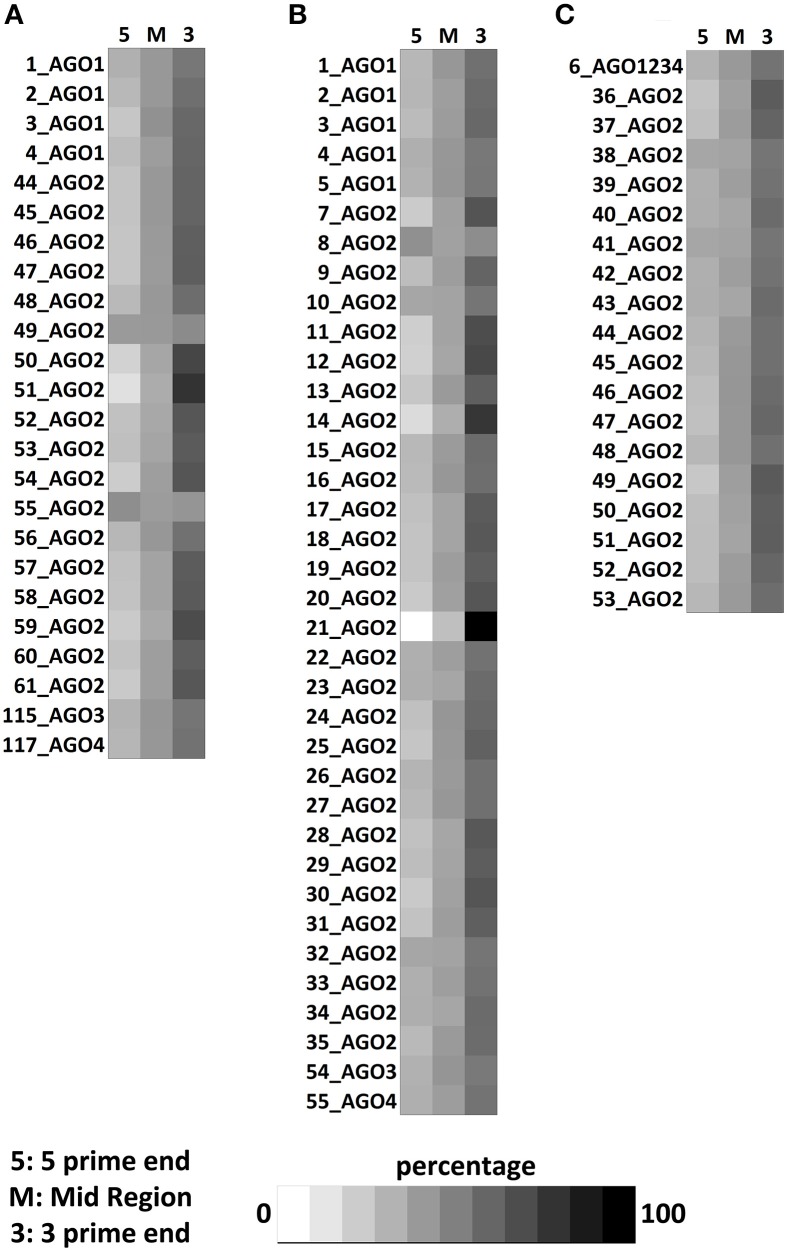
Positional preference of protein-binding sites in lncRNAs transcripts for CLIPdb-CITS. **(A)** Clipdb-PARalyzer, **(B)** starBase, and **(C)** doRiNA.

**Figure 4:**
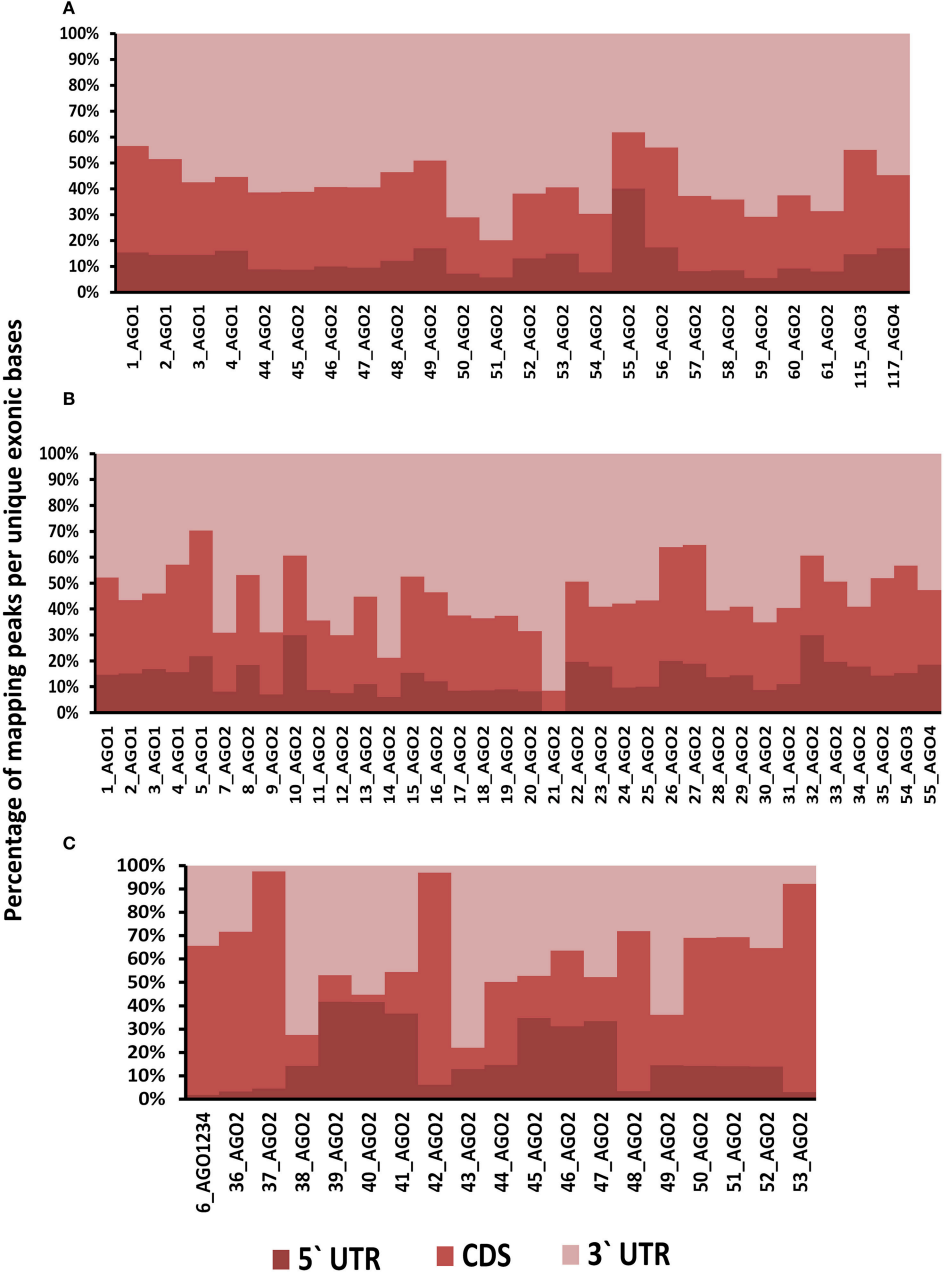
**(A)** Distribution of RNA binding proteins sites from **(A)** Clipdb-PARalyzer, **(B)** starBase, and **(C)** doRiNA datasets across protein coding genes. X-axis of the graph depicts the RBP sites in protein coding genes and Y-axis is the percentage of mapping of these binding sites across the 5′UTR, CDS, and 3′ UTR.

Positional preferences of the RNA binding protein interaction sites were examined across the entire length lncRNAs. The entire length of transcript was calculated by summing up the lengths of individual exons falling in a transcript and then calculating the position of the mapped RNA binding protein interaction site across this calculated length. As the length of the transcript varied therefore, the entire length was arbitrarily divided into three equal parts viz. 5 prime end, middle region, and 3 prime end. Our analysis revealed that the number of RNA binding protein interaction sites for most of the proteins were in majorly mapping to the 3 prime end and the mid segment of the transcripts as shown in Figure 3 and Supplementary Figure 9. To observe the frequencies of binding sites in protein coding transcripts, we mapped and analyzed the RNA binding protein interaction sites in the protein coding transcripts. The binding frequencies for RBPs were evaluated in protein coding transcripts which were divided as 5 prime UTR, CDS, and 3 prime UTR. The data for the same was derived from GENCODE annotation file in form of bed files. We observed that RNA binding protein interaction sites were distributed in 3 prime UTR, 5 prime UTR, and coding exons and frequencies varied for each protein. The HUR/ELAV1 protein showed a positional preference toward the 3 prime end across the lncRNA transcript and the same has been reported recently by Wang and group (Wang et al., 2015) (Figure 4, Supplementary Figures 10, 11).

We further observed that AGO proteins across the three datasets, namely; Clipdb-PARalyzer, starBase, and doRiNA showed to have a positional preference in protein coding and lncRNA transcripts (Figures 3, 4). When we examined the mapping for the three datasets in protein coding transcripts, we observed that AGO protein showed preference toward the 3 prime UTR. Previous reports have shown AGO proteins bound to miRNAs to target toward 3 prime end of mRNA thereby affecting its translation (Pillai et al., 2004). Such positional preference for AGO proteins is an established fact when targeting the 3′ end of mRNAs leading to post-transcriptional silencing. We observed similar positional preference for AGO protein in lncRNAs, thereby suggesting certain regulatory roles.

### High frequencies of RNA binding protein interaction sites in a subset of transcripts

**Figure 5:**
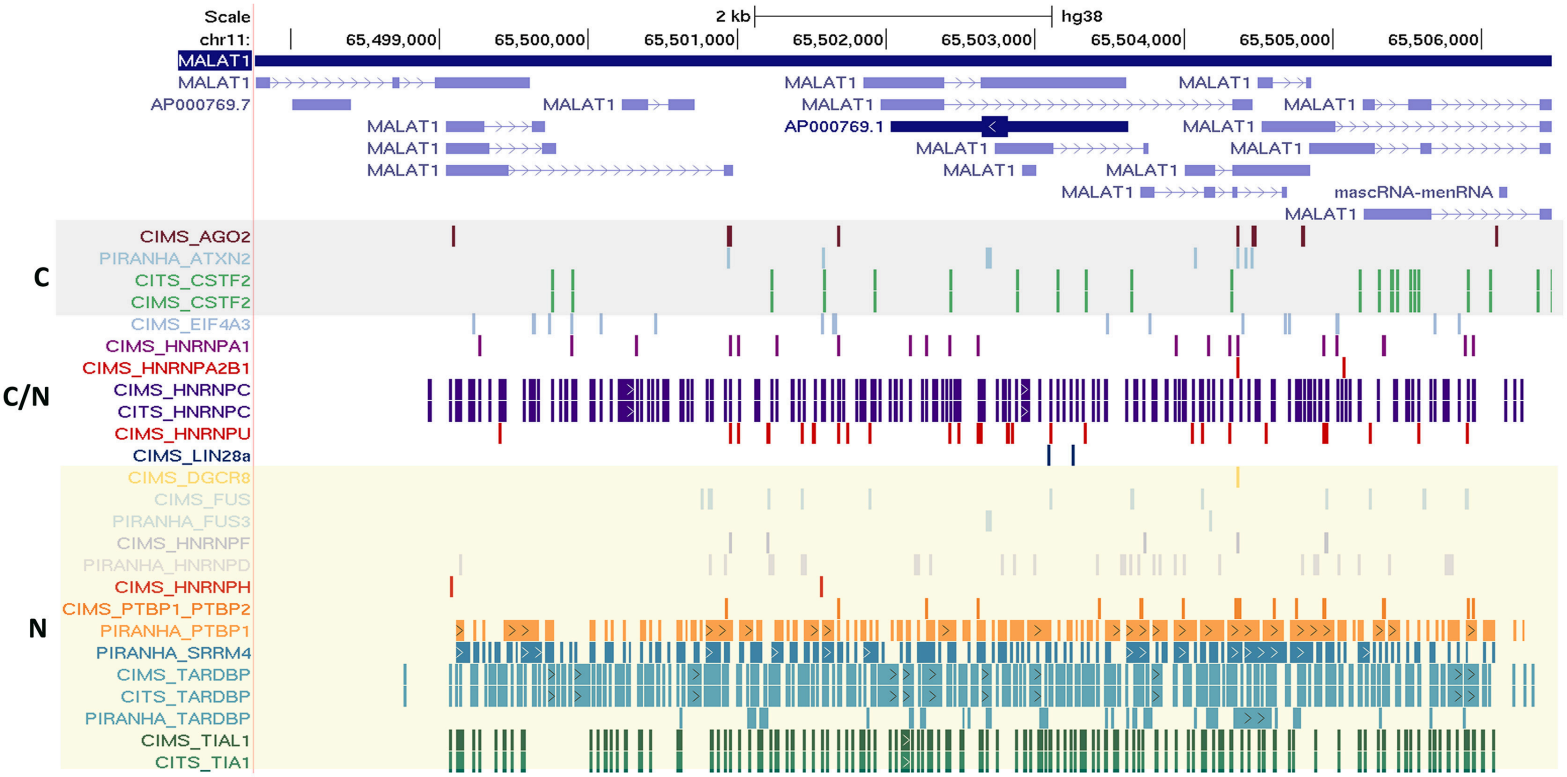
Depiction of the mapping of RNA binding protein interaction sites from CLIPdb-CIMS, CLIPdb-CITS, and CLIPdb-Piranha-stranded datasets across the length of MALAT1 lncRNA. The RBP highlighted in gray box are the ones generally localized to cytoplasm (C). The RBP generally localized to nucleus (N) are marked as yellow box. C/N labeled RBPs is the ones which are present in both Nucleus and Cytoplasm.

We also observed that many well-known lncRNAs including XIST, NEAT1, OIP5-AS1, and MALAT1 had large number of RNA binding protein sites across their length. A subset of well-annotated lncRNA genes had consistently large number of binding sites for majority of the proteins considered. MALAT1 (metastasis associated lung adenocarcinoma transcript 1), a well-studied lncRNA with intricate roles in the pathophysiology of cancer Metastases is one of such candidate (Gutschner et al., 2013). MALAT1 is highly conserved amongst mammals and is known to be localized in nucleus. We plotted the binding sites for all RBPs to the full-length of MALAT1 transcripts and the same is shown in Figure 5 for ClipDB-CIMS, CLIPdb-CITS, and CLIPdb-Piranha-stranded datasets. We combined all the datasets for each protein within a database and divided them into three classes (Cytoplasmic, Nuclear, or Both) based on their cellular localization. The distribution profiles for all the RBPs across the MALAT1 gene was derived using UCSC Genome Browser (Meyer et al., 2013).

We observed that the RBPs known to be localizing in nucleus were shown to have higher binding sites across MALAT1 when compared to other RBPs. The functional interaction of MALAT1 with a number of RNA binding proteins have been previously studied (Tripathi et al., 2010), suggesting extensive functional link to the interactions and thereby providing interesting insights for lncRNA functions and biological regulatory networks they take part in. The mapping for all other datasets across the MALAT1 lncRNA is shown in Supplementary Figures 12, 13.

## Discussion

LncRNAs have lately emerged as one of the major transcript forms encoded by the human genome, the numbers growing as much as the number of protein-coding transcripts over the years. GENCODE v24 has 83,215 lncRNA loci compared to 79,930 protein-coding gene loci. The functional role of many candidate lncRNAs have been extensively studied in the recent past, nevertheless the general lack of conservation of lncRNAs, even between closely related organisms, barring a handful of candidate lncRNAs has restricted the possibility to model functionalities of lncRNAs in model systems.

The availability of genome-scale assays for evaluating protein-binding sites in RNA (Kishore et al., 2011), has offered new opportunities to address this issue at much higher confidence and resolution than which were provided by computational approaches (Bellucci et al., 2011; Puton et al., 2012). To date, seven datasets for genome-scale protein-RNA interactions are available in public domain (i.e., doRiNA, Clipdb, starBase) and the present analysis makes use of all these available datasets. We show such approaches involving repurposing of datasets could provide immense insights into the biological functions with potential regulation of lncRNAs.

In the present study, we have used the peak information (or the most probable site of interaction between protein and RNA) from seven datasets processed through standardized computational pipeline for accurate assessment of protein-RNA interaction sites (doRiNA, Clipdb, starBase). This allowed us to compare the frequencies of the protein binding sites in systematic fashion. It has not escaped our attention that the datasets encompass a diverse set of experiments; cell line, and experimental protocols, nevertheless; our findings hold true despite these differences available in public domain as part of this analysis encompassing six experimental databases of RNA binding proteins. For instance, one of the most studied RBP, the Argonaute datasets showed similar trends regardless of the diverse experimental protocols (HITS-CLIP, iCLIP, PAR-CLIP) and analysis methodologies employed.

The RBPs considered in our study are known to be involved in varied types of functional roles including silencing, splicing, stability, mRNA processing, and transport. In the current study, we observed RBPs enriched for specific lncRNA biotypes are involved in diverse functions, suggesting their probable functional mechanism of action. RBPs such as AGO, DGCR8, EWSR1, TNRC6A/B/C, and FUS, involved in maintenance of the stability of RNA, were having significant enrichment for the lincRNA, miscRNA, retained intron subclasses suggesting they might be acting as either transporters or as sponges for these RBPs. Another set of RBPs such as CPSF complex, FBL, TAF15, and HNRNP family, playing a role in mRNA processing were shown to be enriched in lncRNA subclasses, signifying that lncRNAs inturn might be acting as guides. These proteins might be also involved in mechanism of lncRNA biogenesis. Enrichment was also observed for proteins such ATXN2, C17ORF85, and HNRNPs which predominantly are involved in the export and transporting of RNA moieties, in addition to proteins such as EIF4A3, FOX2, PTBP1, QKI, SFRS1, SRRM4 among others which are predominantly involved in splicing. Hence our analysis suggests that interaction of lncRNA with such types of RBPs surely provide hints about the possible functional roles lncRNAs might be playing which can be validated by experimental approaches.

We also highlight the localization of lncRNAs and RBPs within a cell. We classified the RBPs based on their known localization within the cells and overlapped it with MALAT1, which is an established nuclear enriched lncRNA. The results indicated that the intensity of nuclear localized RBPs were higher for MALAT1 across all the seven datasets. This further strengthened the fact that these bindings were not an arbitrary event and are indeed interacting with the co-localized lncRNAs.

The present analysis reveals a set of interesting characteristics of protein-RNA interaction in the context of lncRNAs: (1) high frequency of RNA-protein interaction sites in lncRNAs subclasses; (2) co-occurrence of RNA binding protein interaction sites; and (3) positional preference for the binding sites across the transcript length. This analysis, to our best of knowledge is the most comprehensive analysis of RNA binding protein interaction sites in lncRNAs, and provides the basis for further analysis on the functional consequences of these patterns. It has also not escaped our attention that targeting protein-interaction sites and thus the functionalities could be in the future therapeutically explored. Recent reports from other laboratories have explored the possibility of targeting RNA structures using small molecules (Jamal et al., 2012; Bose et al., 2013). Further availability of genome-scale protein-RNA interaction datasets and availability of tools to query RNA secondary structures at genome scale (Hofacker, 2003) would provide us with immense opportunities toward understanding the entire repertoire of functional RNA interactions and phenotypic correlates at a genome-scale level. This would also form the much-needed resource of knowledge to potentially query and understand consequences of genomic variations at these loci.

## Conclusion

The interactions between proteins and RNA molecules can provide the essential insights into the functioning of the lncRNAs. In this study, we highlight the enrichment of RBP sites across some of the lncRNA transcript classes in comparison with protein coding transcripts. We have systematically demonstrated that proteins having similar functional roles showed a higher co-occurrence across both lncRNA and protein coding transcripts. Also, the positional preference of most of RBPs agreed with their possible functional roles. Our study gives a compendium of lncRNA and RBP interactions suggesting a large number of functional roles which they can play including silencing, splicing, mRNA processing, export or transport.

## Author contributions

VS conceptualized the analysis. Data analysis was performed by SJ and SG. SJ prepared the data summaries and visualization. SJ and SG wrote the manuscript. All authors reviewed the manuscript.

### Conflict of interest statement

The authors declare that the research was conducted in the absence of any commercial or financial relationships that could be construed as a potential conflict of interest. The reviewer NA and handling Editor declared their shared affiliation.

## Supplementary material

Supplementary Figure 1 **(A,B)** Distribution of RNA binding proteins from CLIPdb-Piranha-stranded across six biotypes of lncRNA genes and protein-coding genes. X-axis of the graph shows the distribution of RNA binding protein interaction sites in subclasses of lncRNAs and protein coding genes frequency of binding sites.

Supplementary Figure 2 Distribution of RNA binding proteins from **(A)** starBase, **(B)** CLIPdb-CITS, **(C)** doRiNA, **(D)** Clipdb-PARalyzer, and **(E)** CLIPdb-Piranha-non-stranded across 6 biotypes of lncRNA genes and protein-coding genes. X-axis of the graph shows the distribution of RNA binding protein interaction sites in subclasses of lncRNAs and protein coding genes frequency of binding sites.

Supplementary Figure 3 Distribution of RNA binding proteins CLIPdb-CIMS (CSTF2), starBase (AGO1), CLIPdb-CITS (HNRNPC), doRiNA (AGO2), Clipdb-PARalyzer (AGO2), and CLIPdb-Piranha-non-stranded (AGO2) across lncRNA, Protein Coding Transcript and Random Genomic Loci. X-axis of the graph represents random RBPs selected from each dataset and Y-axis depicts the normalized frequency of RNA binding protein interaction sites. The frequency is calculated as the number of unique RBP peaks per unique number of exonic bases per kilobase mapped.

Supplementary Figure 4 The Heatmap depicts the combinatorial patterns of clustered protein-binding sites across lncRNAs (blue in color) and protein coding transcripts (red in color) for doRiNA dataset RBPs. The scale here signifies the number of overlapping binding sites per total number of occurrences for the independent proteins.

Supplementary Figure 5 The Heatmap depicts the combinatorial patterns of clustered protein-binding sites across lncRNAs (blue in color) and protein coding transcripts (red in color) for CLIPdb-CIMS dataset RBPs. The scale here signifies the number of overlapping binding sites per total number of occurrences for the independent proteins.

Supplementary Figure 6 The Heatmap depicts the combinatorial patterns of clustered protein-binding sites across lncRNAs (blue in color) and protein coding transcripts (red in color) for CLIPdb-Piranha-non-stranded dataset) RBP. The scale here signifies the number of overlapping binding sites per total number of occurrences for the independent proteins.

Supplementary Figure 7 The Heatmap depicts the combinatorial patterns of clustered protein-binding sites across lncRNAs (blue in color) and protein coding transcripts (red in color) for starBase dataset RBPs. The scale here signifies the number of overlapping binding sites per total number of occurrences for the independent proteins.

Supplementary Figure 8 The Heatmap depicts the combinatorial patterns of clustered protein-binding sites across lncRNAs (blue in color) and protein coding transcripts (red in color) for Clipdb-PARalyzer dataset) RBPs. The scale here signifies the number of overlapping binding sites per total number of occurrences for the independent proteins.

Supplementary Figure 9 Positional preference of protein-binding sites in lncRNAs transcripts for **(A)** Clipdb-PARalyzer, **(B)** CLIPdb-CIMS, **(C)** starBase, **(D)** doRiNA, **(E)** CLIPdb-CITS, and **(F)** CLIPdb-Piranha-stranded.

Supplementary Figure 10 **(A)** Distribution of RNA binding proteins sites from **(A)** Clipdb-PARalyzer, **(B)** CLIPdb-CIMS, and **(C)** starBase datasets across Refseq genes. X-axis of the graph depicts the distribution of RNA binding protein interaction sites in refseq genes and Y-axis is the frequency of binding sites.

Supplementary Figure 11 **(B)** Distribution of RNA binding proteins sites from **(A)** doRiNA, **(B)** CLIPdb-CITS and **(C)** CLIPdb-Piranha-stranded datasets across Refseq genes. X-axis of the graph depicts the distribution of RNA binding protein interaction sites in refseq genes and Y-axis is the frequency of binding sites.

Supplementary Figure 12 Depiction of the mapping of RNA binding protein interaction sites from Clipdb-PARalyzer datasets across the length of MALAT1 lncRNA. The RBP highlighted in gray box are the ones generally localized to cytoplasm (C). The RBP generally localized to nucleus (N) are marked as yellow box. C/N labeled RBPs is the ones which are present in both Nucleus and Cytoplasm.

Supplementary Figure 13 Depiction of the mapping of RNA binding protein interaction sites from ClipDB (doRiNA and starBase datasets) across the length of MALAT1 lncRNA. The RBP highlighted in gray box are the ones generally localized to cytoplasm (C). The RBP generally localized to nucleus (N) are marked as yellow box. C/N labeled RBPs is the ones which are present in both Nucleus and Cytoplasm.

Supplementary Tables 1 **(A)** Detailed list of publically available datasets derieved from Starbase and Dorina databases. **(B)** Detailed list of publically available datasets derieved from CLIPdb database.

## Abbreviations

lncRNA: long non-coding RNA
DNA: Deoxyribonucleic acid
CLIP: cross-linking immunoprecipitation
HITS-CLIP: UV cross-linking and immunoprecipitation with high-throughput sequencing
PAR-CLIP: Photoactivatable Ribonucleoside-Enhanced Crosslinking and Immunoprecipitation
iCLIP: individual-nucleotide resolution Cross-Linking and Immuno Precipitation
ncRNA: Non-coding RNA
lincRNA: long intergenic RNA
TEC: To be Experimentally Confirmed
RIP-seq: RNA Immunoprecipitation sequencing
CLASH: cross-linking ligation and sequencing of hybrids
CIMS: Crosslinking induced mutation site
CITS: crosslinking induced truncation analysis
RISC: RNA-induced silencing complex
RNAi: RNA interference
miRNA: microRNA
RBP: RNA binding protein
UTR: untranslated region
CDS: coding sequence
UCSC: University of California, Santa Cruz.

## References

[B1] Arthold S. Kurowski A. Wutz A. (2011). Mechanistic insights into chromosome-wide silencing in X inactivation. Hum. Genet. 130, 295–305. 10.1007/s00439-011-1002-0 21567178

[B2] Ascano M. Jr. Mukherjee N. Bandaru P. Miller J. B. Nusbaum J. D. Corcoran D. L.. (2012). FMRP targets distinct mRNA sequence elements to regulate protein expression. Nature 492, 382–386. 10.1038/nature11737 23235829 PMC3528815

[B3] Baillat D. Shiekhattar R. (2009). Functional dissection of the human TNRC6 (GW182-related) family of proteins. Mol. Cell. Biol. 29, 4144–4155. 10.1128/MCB.00380-09 19470757 PMC2715800

[B4] Baltz A. G. Munschauer M. Schwanhäusser B. Vasile A. Murakawa Y. Schueler M.. (2012). The mRNA-bound proteome and its global occupancy profile on protein-coding transcripts. Mol. Cell 46, 674–690. 10.1016/j.molcel.2012.05.021 22681889

[B5] Bellucci M. Agostini F. Masin M. Tartaglia G. G. (2011). Predicting protein associations with long noncoding RNAs. Nat. Methods 8, 444–445. 10.1038/nmeth.1611 21623348

[B6] Bhartiya D. Kapoor S. Jalali S. Sati S. Kaushik K. Sachidanandan C.. (2012). Conceptual approaches for lncRNA drug discovery and future strategies. Expert Opin. Drug Discov. 7, 503–513. 10.1517/17460441.2012.682055 22559214

[B7] Blin K. Dieterich C. Wurmus R. Rajewsky N. Landthaler M. Akalin A. (2015). DoRiNA 2.0-upgrading the dorina database of RNA interactions in post-transcriptional regulation. Nucleic Acids Res. 43, D160–D167. 10.1093/nar/gku1180 25416797 PMC4383974

[B8] Blume S. W. Meng Z. Shrestha K. Snyder R. C. Emanuel P. D. (2003). The 5′-untranslated RNA of the human dhfr minor transcript alters transcription pre-initiation complex assembly at the major (core) promoter. J. Cell. Biochem. 88, 165–180. 10.1002/jcb.10326 12461786

[B9] Bose D. Jayaraj G. G. Kumar S. Maiti S. (2013). A molecular-beacon-based screen for small molecule inhibitors of miRNA maturation. ACS Chem. Biol. 8, 930–938. 10.1021/cb300650y 23402670

[B10] Cabili M. N. Trapnell C. Goff L. Koziol M. Tazon-Vega B. Regev A.. (2011). Integrative annotation of human large intergenic noncoding RNAs reveals global properties and specific subclasses. Genes Dev. 25, 1915–1927. 10.1101/gad.17446611 21890647 PMC3185964

[B11] Cao J. (2014). The functional role of long non-coding RNAs and epigenetics. Biol. Proced. Online 16:11. 10.1186/1480-9222-16-11 25276098 PMC4177375

[B12] Chen C.-Y. A. Zheng D. Xia Z. Shyu A.-B. (2009). Ago-TNRC6 triggers microRNA-mediated decay by promoting two deadenylation steps. Nat. Struct. Mol. Biol. 16, 1160–1166. 10.1038/nsmb.1709 19838187 PMC2921184

[B13] Corcoran D. L. Georgiev S. Mukherjee N. Gottwein E. Skalsky R. L. Keene J. D.. (2011). PARalyzer: definition of RNA binding sites from PAR-CLIP short-read sequence data. Genome Biol. 12:R79. 10.1186/gb-2011-12-8-r79 21851591 PMC3302668

[B14] Darnell R. (2012). CLIP (Cross-linking and immunoprecipitation) identification of RNAs bound by a specific protein. Cold Spring Harb. Protoc. 7, 1146–1160. 10.1101/pdb.prot072132 23118367

[B15] Derrien T. Johnson R. Bussotti G. Tanzer A. Djebali S. Tilgner H.. (2012). The GENCODE v7 catalog of human long noncoding RNAs: analysis of their gene structure, evolution, and expression. Genome Res. 22, 1775–1789. 10.1101/gr.132159.111 22955988 PMC3431493

[B16] Elden A. C. Kim H. -J. Hart M. P. Chen-Plotkin A. S. Johnson B. S. Fang X.. (2010). Ataxin-2 intermediate-length polyglutamine expansions are associated with increased risk for ALS. Nature 466, 1069–1075. 10.1038/nature09320 20740007 PMC2965417

[B17] Farazi T. A. Leonhardt C. S. Mukherjee N. Mihailovic A. Li S. Max K. E.. (2014). Identification of the RNA recognition element of the RBPMS family of RNA-binding proteins and their transcriptome-wide mRNA targets. RNA 20, 1090–1102. 10.1261/rna.045005.114 24860013 PMC4114688

[B18] Friedersdorf M. B. Keene J. D. (2014). Advancing the functional utility of PAR-CLIP by quantifying background binding to mRNAs and lncRNAs. Genome Biol. 15:R2. 10.1186/gb-2014-15-1-r2 24393468 PMC4053780

[B19] Furey T. S. (2012). ChIP-seq and beyond: new and improved methodologies to detect and characterize protein-DNA interactions. Nat. Rev. Genet. 13, 840–852. 10.1038/nrg3306 23090257 PMC3591838

[B20] Gibb E. A. Brown C. J. Lam W. L. (2011). The functional role of long non-coding RNA in human carcinomas. Mol. Cancer 10:38. 10.1186/1476-4598-10-38 21489289 PMC3098824

[B21] Goodrich J. A. Kugel J. F. (2006). Non-coding-RNA regulators of RNA polymerase II transcription. Nat. Rev. Mol. Cell Biol. 7, 612–616. 10.1038/nrm1946 16723972

[B22] Gottwein E. Corcoran D. L. Mukherjee N. Skalsky R. L. Hafner M. Nusbaum J. D.. (2011). Viral microRNA targetome of KSHV-Infected primary effusion lymphoma cell lines. Cell Host Microbe 10, 515–526. 10.1016/j.chom.2011.09.012 22100165 PMC3222872

[B23] Graf R. Munschauer M. Mastrobuoni G. Mayr F. Heinemann U. Kempa S.. (2013). Identification of LIN28B-bound mRNAs reveals features of target recognition and regulation. RNA Biol. 10, 1146–1159. 10.4161/rna.25194 23770886 PMC3849162

[B24] Gupta R. A. Shah N. Wang K. C. Kim J. Horlings H. M. Wong D. J.. (2010). Long non-coding RNA HOTAIR reprograms chromatin state to promote cancer metastasis. Nature 464, 1071–1076. 10.1038/nature08975 20393566 PMC3049919

[B25] Gutschner T. Hämmerle M. Eißmann M. Hsu J. Kim Y. Hung G.. (2013). The noncoding RNA MALAT1 is a critical regulator of the metastasis phenotype of lung cancer cells. Cancer Res. 73, 1180–1189. 10.1158/0008-5472.CAN-12-2850 23243023 PMC3589741

[B26] Haecker I. Gay L. A. Yang Y. Hu J. Morse A. M. McIntyre L. M.. (2012). Ago HITS-CLIP expands understanding of Kaposi's sarcoma-associated herpesvirus miRNA function in primary effusion Lymphomas. PLoS Pathog. 8:e1002884. 10.1371/journal.ppat.1002884 22927820 PMC3426530

[B27] Hafner M. Landthaler M. Burger L. Khorshid M. Hausser J. Berninger P.. (2010a). PAR-CliP–a method to identify transcriptome-wide the binding sites of RNA binding proteins. J. Vis. Exp. 2034. 10.3791/2034 20644507 PMC3156069

[B28] Hafner M. Landthaler M. Burger L. Khorshid M. Hausser J. Berninger P.. (2010b). Transcriptome-wide identification of RNA-binding protein and MicroRNA target sites by PAR-CLIP. Cell 141, 129–141. 10.1016/j.cell.2010.03.009 20371350 PMC2861495

[B29] Hafner M. Max K. E. A. Bandaru P. Morozov P. Gerstberger S. Brown M.. (2013). Identification of mRNAs bound and regulated by human LIN28 proteins and molecular requirements for RNA recognition. RNA 19, 613–626. 10.1261/rna.036491.112 23481595 PMC3677277

[B30] Harrow J. Frankish A. Gonzalez J. M. Tapanari E. Diekhans M. Kokocinski F.. (2012). GENCODE: the reference human genome annotation for the ENCODE project. Genome Res. 22, 1760–1774. 10.1101/gr.135350.111 22955987 PMC3431492

[B31] Helwak A. Kudla G. Dudnakova T. Tollervey D. (2013). Mapping the human miRNA interactome by CLASH reveals frequent noncanonical binding. Cell 153, 654–665. 10.1016/j.cell.2013.03.043 23622248 PMC3650559

[B32] Hoell J. I. Larsson E. Runge S. Nusbaum J. D. Duggimpudi S. Farazi T. A.. (2011). RNA targets of wild-type and mutant FET family proteins. Nat. Struct. Mol. Biol. 18, 1428–1431. 10.1038/nsmb.2163 22081015 PMC3230689

[B33] Hofacker I. L. (2003). Vienna RNA secondary structure server. Nucleic Acids Res. 31, 3429–3431. 10.1093/nar/gkg599 12824340 PMC169005

[B34] Huelga S. C. Vu A. Q. Arnold J. D. Liang T. D. Liu P. P. Yan B. Y.. (2012). Integrative genome-wide analysis reveals cooperative regulation of alternative splicing by hnRNP proteins. Cell Rep. 1, 167–178. 10.1016/j.celrep.2012.02.001 22574288 PMC3345519

[B35] Ibrahim F. Maragkakis M. Alexiou P. Maronski M. A. Dichter M. A. Mourelatos Z. (2013). Identification of *in vivo*, Conserved, TAF15 RNA binding sites reveals the impact of TAF15 on the neuronal transcriptome. Cell Rep. 3, 301–308. 10.1016/j.celrep.2013.01.021 23416048 PMC3594071

[B36] Jain R. Devine T. George A. D. Chittur S. V. Baroni T. E. Penalva L. O.. (2011). RIP-chip analysis: RNA-binding protein immunoprecipitation-microarray (chip) profiling. Methods Mol. Biol. 703, 247–263. 10.1007/978-1-59745-248-9_17 21125495

[B37] Jalali S. Bhartiya D. Lalwani M. K. Sivasubbu S. Scaria V. (2013). Systematic transcriptome wide analysis of lncRNA-miRNA interactions. PLoS ONE 8:e53823. 10.1371/journal.pone.0053823 23405074 PMC3566149

[B38] Jalali S. Jayaraj G. Scaria V. Kapranov P. Cheng J. Dike S.. (2012). Integrative transcriptome analysis suggest processing of a subset of long non-coding RNAs to small RNAs. Biol. Direct 7:25. 10.1186/1745-6150-7-25 22871084 PMC3477000

[B39] Jalali S. Kapoor S. Sivadas A. Bhartiya D. Scaria V. (2015). Computational approaches towards understanding human long non-coding RNA biology. Bioinformatics 31, 2241–2251. 10.1093/bioinformatics/btv148 25777523

[B40] Jamal S. Periwal V. Scaria V. (2012). Computational analysis and predictive modeling of small molecule modulators of microRNA. J. Cheminform. 4:16. 10.1186/1758-2946-4-16 22889302 PMC3466443

[B41] Kaneko S. Son J. Shen S. S. Reinberg D. Bonasio R. (2013). PRC2 binds active promoters and contacts nascent RNAs in embryonic stem cells. Nat. Struct. Mol. Biol. 20, 1258–1264. 10.1038/nsmb.2700 24141703 PMC3839660

[B42] Karginov F. V. Hannon G. J. (2013). Remodeling of Ago2-mRNA interactions upon cellular stress reflects miRNA complementarity and correlates with altered translation rates. Genes Dev. 27, 1624–1632. 10.1101/gad.215939.113 23824327 PMC3731550

[B43] Katz Y. Wang E. T. Airoldi E. M. Burge C. B. (2010). Analysis and design of RNA sequencing experiments for identifying isoform regulation. Nat. Methods 7, 1009–1015. 10.1038/nmeth.1528 21057496 PMC3037023

[B44] Kino T. Hurt D. E. Ichijo T. Nader N. Chrousos G. P. (2010). Noncoding RNA gas5 is a growth arrest- and starvation-associated repressor of the glucocorticoid receptor. Sci. Signal. 3:ra8. 10.1126/scisignal.2000568 20124551 PMC2819218

[B45] Kishore S. Gruber A. R. Jedlinski D. J. Syed A. P. Jorjani H. Zavolan M. (2013). Insights into snoRNA biogenesis and processing from PAR-CLIP of snoRNA core proteins and small RNA sequencing. Genome Biol. 14:R45. 10.1186/gb-2013-14-5-r45 23706177 PMC4053766

[B46] Kishore S. Jaskiewicz L. Burger L. Hausser J. Khorshid M. Zavolan M. (2011). A quantitative analysis of CLIP methods for identifying binding sites of RNA-binding proteins. Nat. Methods 8, 559–564. 10.1038/nmeth.1608 21572407

[B47] Kogo R. Shimamura T. Mimori K. Kawahara K. Imoto S. Sudo T.. (2011). Long noncoding RNA HOTAIR regulates polycomb-dependent chromatin modification and is associated with poor prognosis in colorectal cancers. Cancer Res. 71, 6320–6326. 10.1158/0008-5472.CAN-11-1021 21862635

[B48] Konig J. Zarnack K. Rot G. Curk T. Kayikci M. Zupan B.. (2011). iCLIP–transcriptome-wide mapping of protein-RNA interactions with individual nucleotide resolution. J. Vis. Exp. 2638. 10.3791/2638 21559008 PMC3169244

[B49] Kung J. T. Y. Colognori D. Lee J. T. (2013). Long noncoding RNAs: past, present, and future. Genetics 193, 651–669. 10.1534/genetics.112.146704 23463798 PMC3583990

[B50] Lagier-Tourenne C. Polymenidou M. Hutt K. R. Vu A. Q. Baughn M. Huelga S. C.. (2012). Divergent roles of ALS-linked proteins FUS/TLS and TDP-43 intersect in processing long pre-mRNAs. Nat. Neurosci. 15, 1488–1497. 10.1038/nn.3230 23023293 PMC3586380

[B51] Lebedeva S. Jens M. Theil K. Schwanhäusser B. Selbach M. Landthaler M.. (2011). Transcriptome-wide analysis of regulatory interactions of the RNA-binding protein HuR. Mol. Cell 43, 340–352. 10.1016/j.molcel.2011.06.008 21723171

[B52] Lee J. T. Bartolomei M. S. (2013). X-inactivation, imprinting, and long noncoding RNAs in health and disease. Cell 152, 1308–1323. 10.1016/j.cell.2013.02.016 23498939

[B53] Li J. H. Liu S. Zhou H. Qu L. H. Yang J. H. (2014). StarBase v2.0: decoding miRNA-ceRNA, miRNA-ncRNA and protein-RNA interaction networks from large-scale CLIP-Seq data. Nucleic Acids Res. 42, D92–D97. 10.1093/nar/gkt1248 24297251 PMC3964941

[B54] Lipchina I. Elkabetz Y. Hafner M. Sheridan R. Mihailovic A. Tuschl T.. (2011). Genome-wide identification of microRNA targets in human ES cells reveals a role for miR-302 in modulating BMP response. Genes Dev. 25, 2173–2186. 10.1101/gad.17221311 22012620 PMC3205587

[B55] Livi C. M. Klus P. Delli Ponti R. Tartaglia G. G. (2015). CatRAPID signature: identification of ribonucleoproteins and RNA-binding regions. Bioinformatics 32, 773–775. 10.1093/bioinformatics/btv629 26520853 PMC4795616

[B56] Macias S. Plass M. Stajuda A. Michlewski G. Eyras E. Cáceres J. F.. (2012). DGCR8 HITS-CLIP reveals novel functions for the microprocessor. Nat. Struct. Mol. Biol. 19, 760–766. 10.1038/nsmb.2344 22796965 PMC3442229

[B57] Martin G. Gruber A. R. Keller W. Zavolan M. (2012). Genome-wide analysis of pre-mRNA 3′ end processing reveals a decisive role of human cleavage factor I in the regulation of 3′ UTR length. Cell Rep. 1, 753–763. 10.1016/j.celrep.2012.05.003 22813749

[B58] Memczak S. Jens M. Elefsinioti A. Torti F. Krueger J. Rybak A.. (2013). Circular RNAs are a large class of animal RNAs with regulatory potency. Nature 495, 333–338. 10.1038/nature11928 23446348

[B59] Mercer T. R. Dinger M. E. Mattick J. S. (2009). Long non-coding RNAs: insights into functions. Nat. Rev. Genet. 10, 155–159. 10.1038/nrg2521 19188922

[B60] Meyer L. R. Zweig A. S. Hinrichs A. S. Karolchik D. Kuhn R. M. Wong M.. (2013). The UCSC genome browser database: extensions and updates 2013. Nucleic Acids Res. 41, D64–D69. 10.1093/nar/gks1048 23155063 PMC3531082

[B61] Moore M. J. Zhang C. Gantman E. C. Mele A. Darnell J. C. Darnell R. B. (2014). Mapping argonaute and conventional RNA-binding protein interactions with RNA at single-nucleotide resolution using HITS-CLIP and CIMS analysis. Nat. Protoc. 9, 263–293. 10.1038/nprot.2014.012 24407355 PMC4156013

[B62] Mukherjee N. (2011). Integrative regulatory mapping indicates that the RNA-binding protein HuR couples pre-mRNA processing and mRNA stability. Mol. Cell 43, 327–339. 10.1016/j.molcel.2011.06.007 21723170 PMC3220597

[B63] Nakaya T. Alexiou P. Maragkakis M. Chang A. Mourelatos Z. (2013). FUS regulates genes coding for RNA-binding proteins in neurons by binding to their highly conserved introns. RNA 19, 498–509. 10.1261/rna.037804.112 23389473 PMC3677260

[B64] Park C. Yu N. Choi I. Kim W. Lee S. (2014). LncRNAtor: a comprehensive resource for functional investigation of long non-coding RNAs. Bioinformatics 30, 2480–2485. 10.1093/bioinformatics/btu325 24813212

[B65] Paronetto M. P. Bernardis I. Volpe E. Bechara E. Sebestyén E. Eyras E.. (2014). Regulation of FAS exon definition and apoptosis by the ewing sarcoma protein. Cell Rep. 7, 1211–1226. 10.1016/j.celrep.2014.03.077 24813895

[B66] Pauli A. Valen E. Lin M. F. Garber M. Vastenhouw N. L. Levin J. Z.. (2012). Systematic identification of long noncoding RNAs expressed during zebrafish embryogenesis. Genome Res. 22, 577–591. 10.1101/gr.133009.111 22110045 PMC3290793

[B67] Pillai R. S. Artus C. G. Filipowicz W. (2004). Tethering of human Ago proteins to mRNA mimics the miRNA-mediated repression of protein synthesis. RNA 10, 1518–1525. 10.1261/rna.7131604 15337849 PMC1370638

[B68] Ping X.-L. Sun B. -F. Wang L. Xiao W. Yang X. Wang W. -J.. (2014). Mammalian WTAP is a regulatory subunit of the RNA N6-methyladenosine methyltransferase. Cell Res. 24, 177–189. 10.1038/cr.2014.3 24407421 PMC3915904

[B69] Popov N. Gil J. (2010). Epigenetic regulation of the INK4B-ARF-INK4a locus: in sickness and in health. Epigenetics 5, 685–690. 10.4161/epi.5.8.12996 20716961 PMC3052884

[B70] Puton T. Kozlowski L. Tuszynska I. Rother K. Bujnicki J. M. (2012). Computational methods for prediction of protein-RNA interactions. J. Struct. Biol. 179, 261–268. 10.1016/j.jsb.2011.10.001 22019768

[B71] Quinlan A. R. Hall I. M. (2010). BEDTools: a flexible suite of utilities for comparing genomic features. Bioinformatics 26, 841–842. 10.1093/bioinformatics/btq033 20110278 PMC2832824

[B72] Raj B. Irimia M. Braunschweig U. Sterne-Weiler T. O'Hanlon D. Lin Z. Y.. (2014). A global regulatory mechanism for activating an exon network required for neurogenesis. Mol. Cell 56, 90–103. 10.1016/j.molcel.2014.08.011 25219497 PMC4608043

[B73] R Core Team (2015). R: A Language and Environment for Statistical Computing. Vienna: R Found. Stat. Comput Available online at: http://www.r-project.org

[B74] Riley K. J. Rabinowitz G. S. Yario T. A. Luna J. M. Darnell R. B. Steitz J. A. (2012). EBV and human microRNAs co-target oncogenic and apoptotic viral and human genes during latency. EMBO J. 31, 2207–2221. 10.1038/emboj.2012.63 22473208 PMC3343464

[B75] Roberts A. Pimentel H. Trapnell C. Pachter L. (2011). Identification of novel transcripts in annotated genomes using RNA-seq. Bioinformatics 27, 2325–2329. 10.1093/bioinformatics/btr355 21697122

[B76] Salmena L. Poliseno L. Tay Y. Kats L. Pandolfi P. P. (2011). A ceRNA hypothesis: the rosetta stone of a hidden RNA language? Cell 146, 353–358. 10.1016/j.cell.2011.07.014 21802130 PMC3235919

[B77] Sanford J. R. Wang X. Mort M. Vanduyn N. Cooper D. N. Mooney S. D.. (2009). Splicing factor SFRS1 recognizes a functionally diverse landscape of RNA transcripts. Genome Res. 19, 381–394. 10.1101/gr.082503.108 19116412 PMC2661799

[B78] Sati S. Ghosh S. Jain V. Scaria V. Sengupta S. (2012). Genome-wide analysis reveals distinct patterns of epigenetic features in long non-coding RNA loci. Nucleic Acids Res. 40, 10018–10031. 10.1093/nar/gks776 22923516 PMC3488231

[B79] Saulière J. Murigneux V. Wang Z. Marquenet E. Barbosa I. Le Tonquèze O. (2012). CLIP-seq of eIF4AIII reveals transcriptome-wide mapping of the human exon junction complex. TL - 19. Nat. Struct. Mol. Biol. 19, 1124–1131. 10.1038/nsmb.2420 23085716

[B80] Schönemann L. Kühn U. Martin G. Schäfer P. Gruber A. R. Keller W.. (2014). Reconstitution of CPSF active in polyadenylation: recognition of the polyadenylation signal by WDR33. Genes Dev. 28, 2381–2393. 10.1101/gad.250985.114 25301781 PMC4215183

[B81] Shankarling G. Cole B. S. Mallory M. J. Lynch K. W. (2014). Transcriptome-wide RNA interaction profiling reveals physical and functional targets of hnRNP L in human T cells. Mol. Cell. Biol. 34, 71–83. 10.1128/MCB.00740-13 24164894 PMC3911283

[B82] Sheik Mohamed J. Gaughwin P. M. Lim B. Robson P. Lipovich L. (2010). Conserved long noncoding RNAs transcriptionally regulated by Oct4 and Nanog modulate pluripotency in mouse embryonic stem cells. RNA 16, 324–337. 10.1261/rna.1441510 20026622 PMC2811662

[B83] Sievers C. Schlumpf T. Sawarkar R. Comoglio F. Paro R. (2012). Mixture models and wavelet transforms reveal high confidence RNA-protein interaction sites in MOV10 PAR-CLIP data. Nucleic Acids Res. 40:e160. 10.1093/nar/gks697 22844102 PMC3488208

[B84] Skalsky R. L. Corcoran D. L. Gottwein E. Frank C. L. Kang D. Hafner M.. (2012). The viral and cellular microRNA targetome in lymphoblastoid cell lines. PLoS Pathog. 8:e1002484. 10.1371/journal.ppat.1002484 22291592 PMC3266933

[B85] Tollervey J. R. Curk T. Rogelj B. Briese M. Cereda M. Kayikci M.. (2011). Characterizing the RNA targets and position-dependent splicing regulation by TDP-43. Nat. Neurosci. 14, 452–458. 10.1038/nn.2778 21358640 PMC3108889

[B86] Tripathi V. Ellis J. D. Shen Z. Song D. Y. Pan Q. Watt A. T.. (2010). The nuclear-retained noncoding RNA MALAT1 regulates alternative splicing by modulating SR splicing factor phosphorylation. Mol. Cell 39, 925–938. 10.1016/j.molcel.2010.08.011 20797886 PMC4158944

[B87] Ule J. Wang Z. Kayikci M. Briese M. Zarnack K. Luscombe N. M.. (2010). iCLIP predicts the dual splicing effects of TIA-RNA interactions. PLoS Biol. 8:e1000530. 10.1371/journal.pbio.1000530 21048981 PMC2964331

[B88] Uren P. J. Bahrami-Samani E. Burns S. C. Qiao M. Karginov F. V. Hodges E.. (2012). Site identification in high-throughput RNA-protein interaction data. Bioinformatics 28, 3013–3020. 10.1093/bioinformatics/bts569 23024010 PMC3509493

[B89] Wang Z. Bhattacharya A. Ivanov D. N. (2015). Identification of Small-Molecule Inhibitors of the HuR/RNA Interaction using a fluorescence polarization screening assay followed by NMR validation. PLoS. One. 10:e0138780. 10.1371/journal.pone.0138780 26390015 PMC4577092

[B90] Wang X. Lu Z. Gomez A. Hon G. C. Yue Y. Han D.. (2014). N6-methyladenosine-dependent regulation of messenger RNA stability. Nature 505, 117–120. 10.1038/nature12730 24284625 PMC3877715

[B91] Wang Y. Gogol-Döring A. Hu H. Fröhler S. Ma Y. Jens M.. (2013). Integrative analysis revealed the molecular mechanism underlying RBM10-mediated splicing regulation. EMBO Mol. Med. 5, 1431–1442. 10.1002/emmm.201302663 24000153 PMC3799496

[B92] Wapinski O. Chang H. Y. (2011). Long noncoding RNAs and human disease. Trends Cell Biol. 21, 354–361. 10.1016/j.tcb.2011.04.001 21550244

[B93] Weyn-Vanhentenryck S. M. Mele A. Yan Q. Sun S. Farny N. Zhang Z.. (2014). HITS-CLIP and integrative modeling define the Rbfox splicing-regulatory network linked to brain development and autism. Cell Rep. 6, 1139–1152. 10.1016/j.celrep.2014.02.005 24613350 PMC3992522

[B94] Wilbert M. L. Huelga S. C. Kapeli K. Stark T. J. Liang T. Y. Chen S. X.. (2012). LIN28 binds messenger RNAs at GGAGA motifs and regulates splicing factor abundance. Mol. Cell 48, 195–206. 10.1016/j.molcel.2012.08.004 22959275 PMC3483422

[B95] Wilusz J. E. Sunwoo H. Spector D. L. (2009). Long noncoding RNAs: functional surprises from the RNA world. Genes Dev. 23, 1494–1504. 10.1101/gad.1800909 19571179 PMC3152381

[B96] Xiao R. Tang P. Yang B. Huang J. Zhou Y. Shao C.. (2012). Nuclear matrix factor hnRNP U/SAF-A exerts a global control of alternative splicing by regulating U2 snRNP maturation. Mol. Cell 45, 656–668. 10.1016/j.molcel.2012.01.009 22325991 PMC3299905

[B97] Xue Y. Ouyang K. Huang J. Zhou Y. Ouyang H. Li H.. (2013). Direct conversion of fibroblasts to neurons by reprogramming PTB-regulated microRNA circuits. Cell 152, 82–96. 10.1016/j.cell.2012.11.045 23313552 PMC3552026

[B98] Xue Y. Zhou Y. Wu T. Zhu T. Ji X. Kwon Y. S. (2009). Genome-wide analysis of PTB-RNA Interactions reveals a strategy used by the general splicing repressor to modulate exon inclusion or Skipping. Mol. Cell 36, 996–1006. 10.1016/j.molcel.2009.12.003 20064465 PMC2807993

[B99] Yang J. H. Li J. H. Shao P. Zhou H. Chen Y. Q. Qu L. H. (2011). StarBase: a database for exploring microRNA-mRNA interaction maps from Argonaute CLIP-Seq and degradome-Seq data. Nucleic Acids Res. 39, D202–D209. 10.1093/nar/gkq1056 21037263 PMC3013664

[B100] Yang Y.-C. T. Di C. Hu B. Zhou M. Liu Y. Song N.. (2015). CLIPdb: a CLIP-seq database for protein-RNA interactions. BMC Genomics 16:51. 10.1186/s12864-015-1273-2 25652745 PMC4326514

[B101] Yao C. Biesinger J. Wan J. Weng L. Xing Y. Xie X.. (2012). Transcriptome-wide analyses of CstF64–RNA interactions in global regulation of mRNA alternative polyadenylation. Proc. Natl. Acad. Sci. U.S.A. 109, 18773–18778. 10.1073/pnas.1211101109 23112178 PMC3503179

[B102] Yeo G. W. Coufal N. G. Liang T. Y. Peng G. E. Fu X. -D. Gage F. H. (2009). An RNA code for the FOX2 splicing regulator revealed by mapping RNA-protein interactions in stem cells. Nat. Struct. Mol. Biol. 16, 130–137. 10.1038/nsmb.1545 19136955 PMC2735254

[B103] Yokoshi M. Li Q. Yamamoto M. Okada H. Suzuki Y. Kawahara Y. (2014). Direct binding of Ataxin-2 to distinct elements in 3′ UTRs promotes mRNA stability and protein expression. Mol. Cell 55, 186–198. 10.1016/j.molcel.2014.05.022 24954906

[B104] Yoon J. -H. De S. Srikantan S. Abdelmohsen K. Grammatikakis I. Kim J.. (2014). PAR-CLIP analysis uncovers AUF1 impact on target RNA fate and genome integrity. Nat. Commun. 5, 1–15. 10.1038/ncomms6248 25366541 PMC4291169

[B105] Zarnack K. König J. Tajnik M. Martincorena I. Eustermann S. Stévant I.. (2013). Direct competition between hnRNP C and U2AF65 protects the transcriptome from the exonization of alu elements. Cell 152, 453–466. 10.1016/j.cell.2012.12.023 23374342 PMC3629564

[B106] Zarnack K. Rot G. Kayikci M. Turner D. J. Luscombe N. M. Ule J. (2010). Technical reports. Nat. Struct. Mol. Biol. 17, 909–916. 10.1038/nsmb.1838 20601959 PMC3000544

[B107] Zhao H. Sun Z. Wang J. Huang H. Kocher J. P. Wang L. (2014). CrossMap: a versatile tool for coordinate conversion between genome assemblies. Bioinformatics 30, 1006–1007. 10.1093/bioinformatics/btt730 24351709 PMC3967108

[B108] Zhao J. Zhang X. Zhou Y. Ansell P. J. Klibanski A. (2006). Cyclic AMP stimulates MEG3 gene expression in cells through a cAMP-response element (CRE) in the MEG3 proximal promoter region. Int. J. Biochem. Cell Biol. 38, 1808–1820. 10.1016/j.biocel.2006.05.004 16793321

[B109] Zünd D. Gruber A. R. Zavolan M. Mühlemann O. (2013). Translation-dependent displacement of UPF1 from coding sequences causes its enrichment in 3′ UTRs. Nat. Struct. Mol. Biol. 20, 936–943. 10.1038/nsmb.2635 23832275

