# Supplementary figures and images for: Distinct and Modular Organization of Protein Interacting Sites in Long Non-coding RNAs
Source: Front Mol Biosci. 2018 Apr 4;5:27. doi: 10.3389/fmolb.2018.00027 (PMC5893854; doi:10.3389/fmolb.2018.00027)

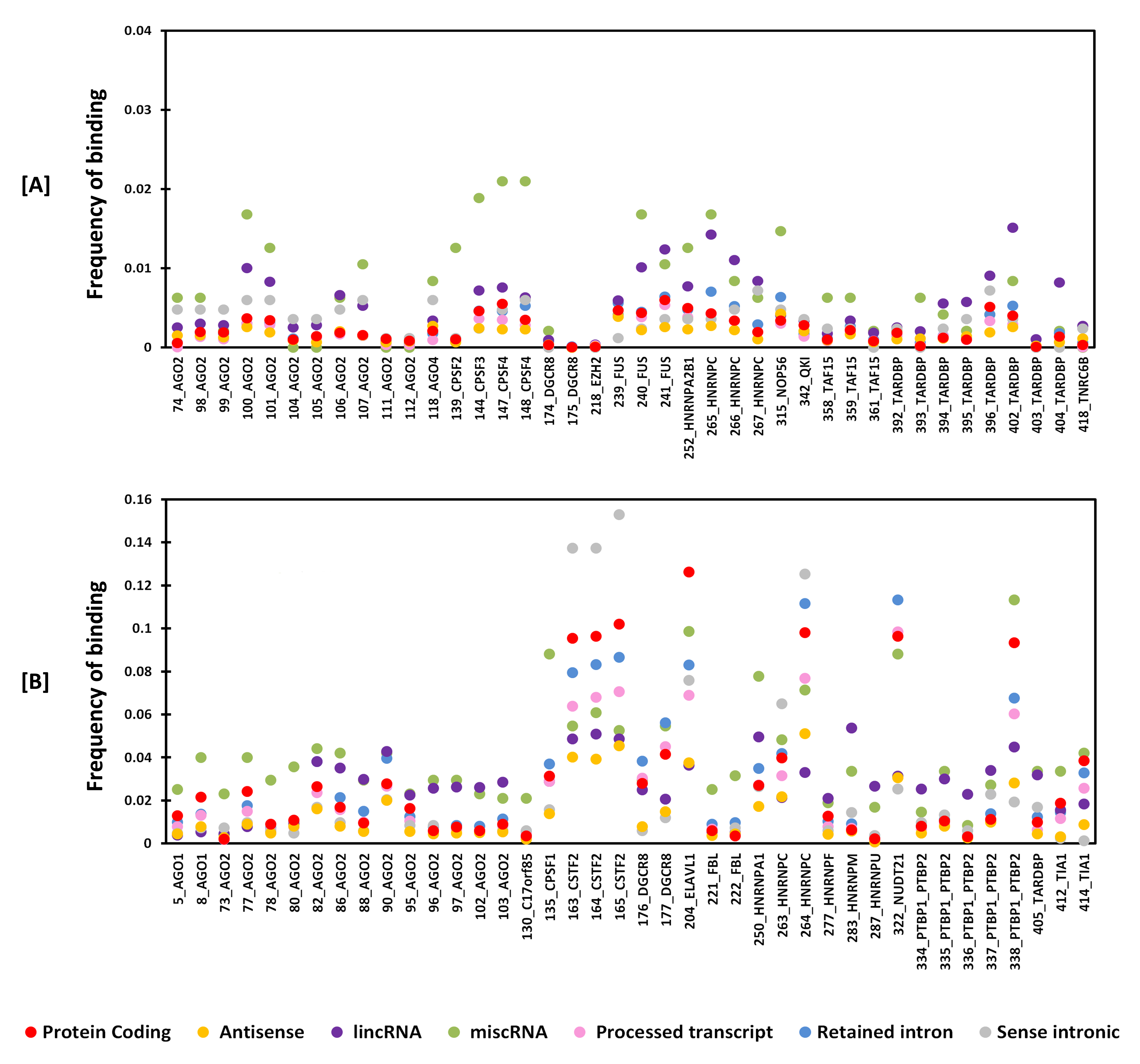

Supplement: Supplementary Figure 1 — (A,B) Distribution of RNA binding proteins from CLIPdb-Piranha-stranded across six biotypes of lncRNA genes and protein-coding genes. X-axis of the graph shows the distribution of RNA binding protein interaction sites in subclasses of lncRNAs and protein coding genes frequency of binding sites. [file Image1.JPEG]

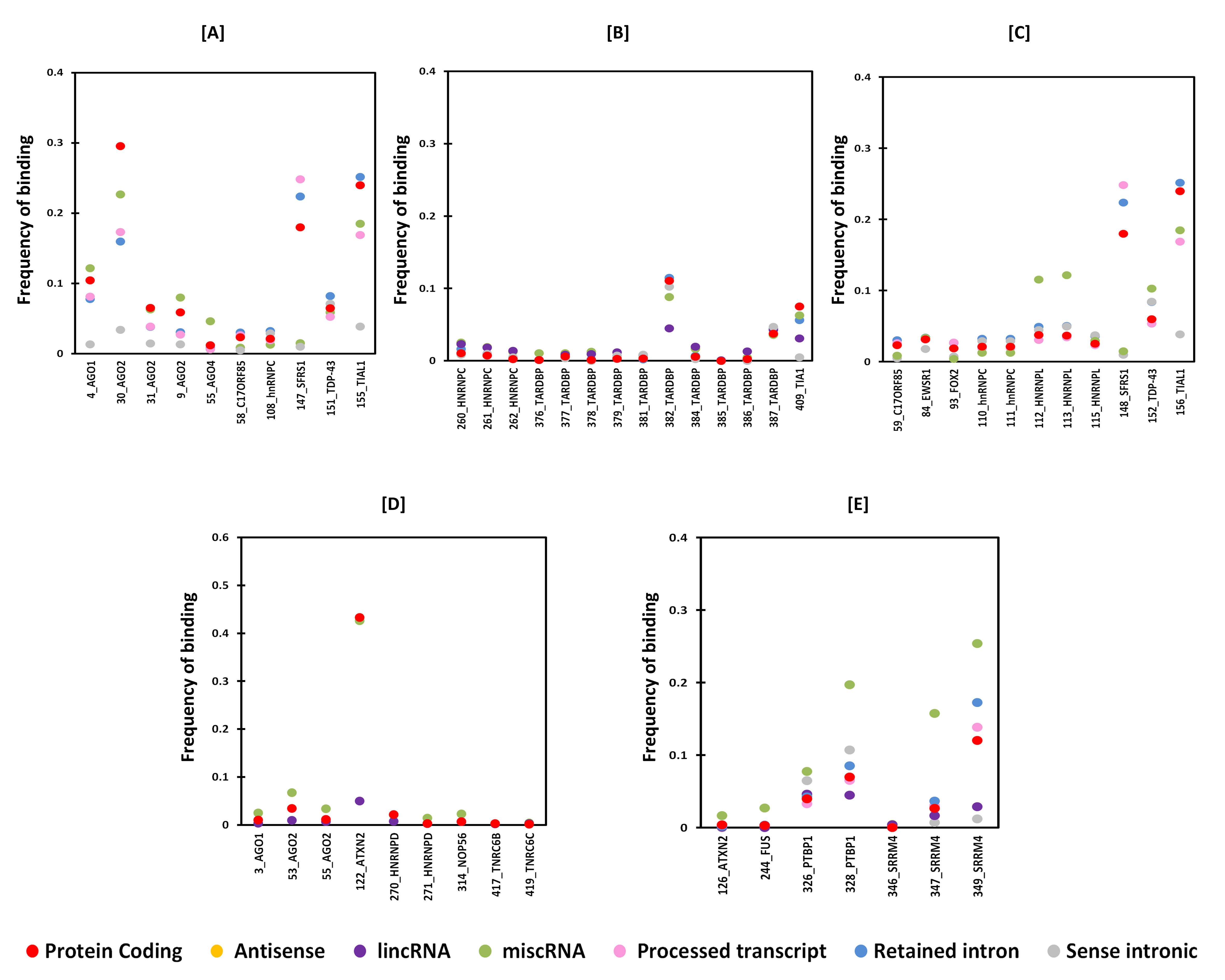

Supplement: Supplementary Figure 2 — Distribution of RNA binding proteins from (A) starBase, (B) CLIPdb-CITS, (C) doRiNA, (D) Clipdb-PARalyzer, and (E) CLIPdb-Piranha-non-stranded across 6 biotypes of lncRNA genes and protein-coding genes. X-axis of the graph shows the distribution of RNA binding protein interaction sites in subclasses of lncRNAs and protein coding genes frequency of binding sites. [file Image2.JPEG]

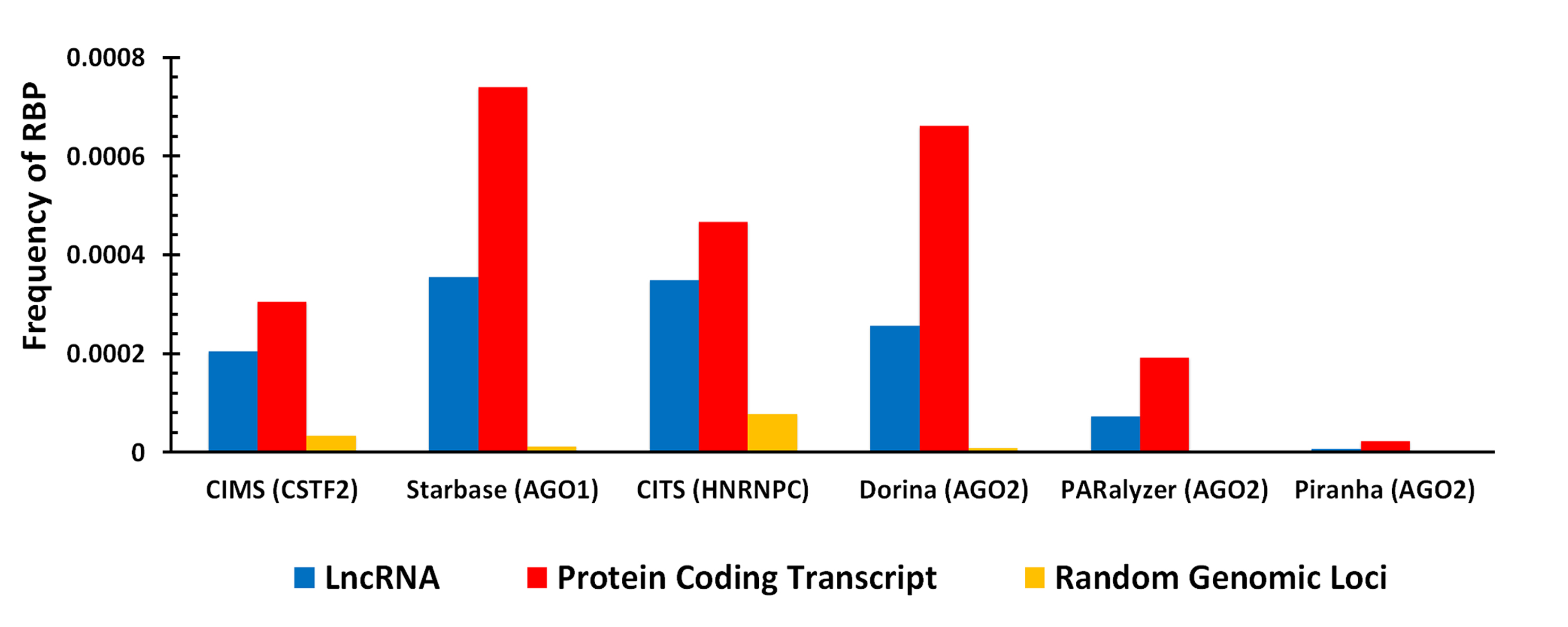

Supplement: Supplementary Figure 3 — Distribution of RNA binding proteins CLIPdb-CIMS (CSTF2), starBase (AGO1), CLIPdb-CITS (HNRNPC), doRiNA (AGO2), Clipdb-PARalyzer (AGO2), and CLIPdb-Piranha-non-stranded (AGO2) across lncRNA, Protein Coding Transcript and Random Genomic Loci. X-axis of the graph represents random RBPs selected from each dataset and Y-axis depicts the normalized frequency of RNA binding protein interaction sites. The frequency is calculated as the number of unique RBP peaks per unique number of exonic bases per kilobase mapped. [file Image3.JPEG]

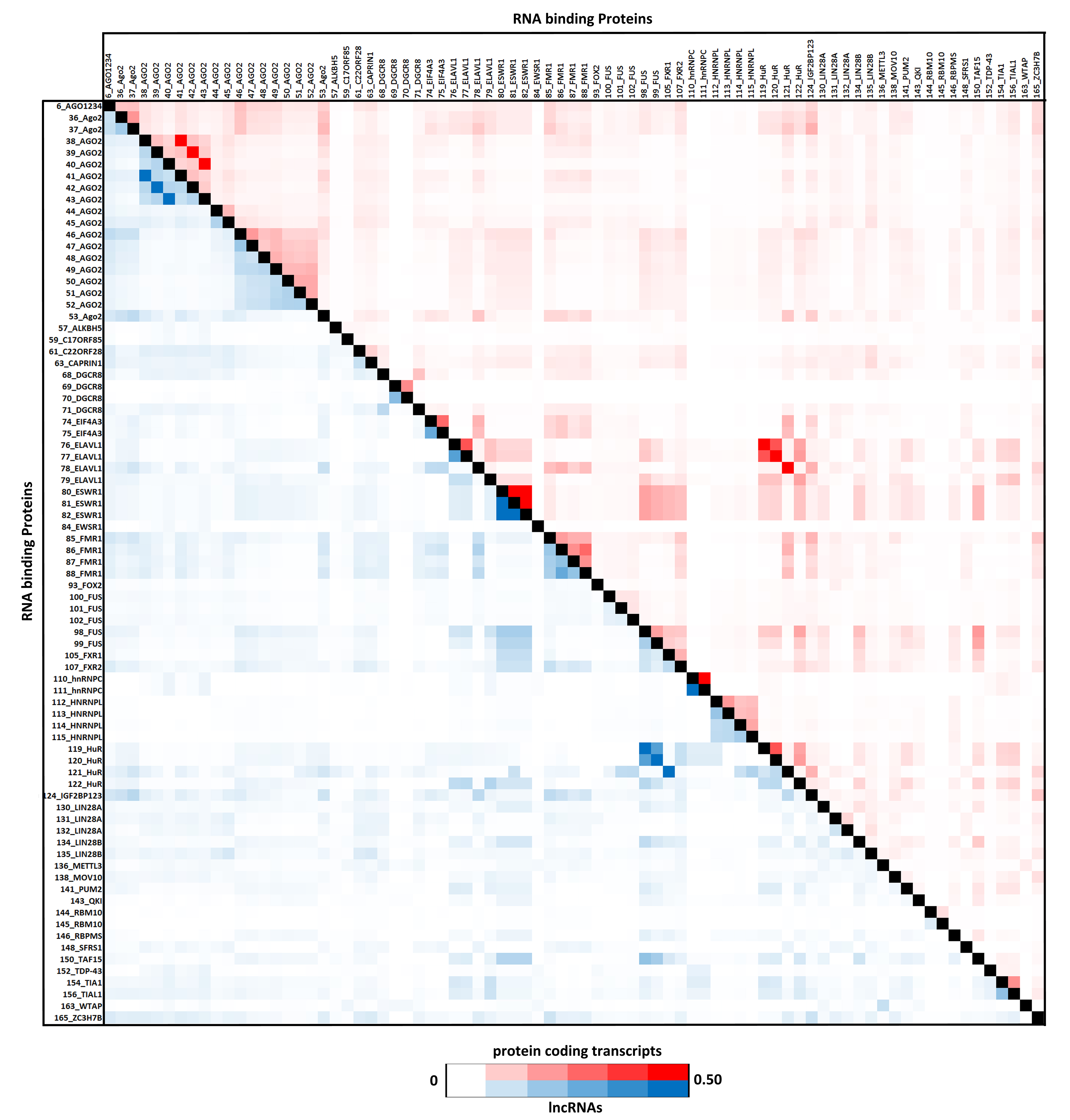

Supplement: Supplementary Figure 4 — The Heatmap depicts the combinatorial patterns of clustered protein-binding sites across lncRNAs (blue in color) and protein coding transcripts (red in color) for doRiNA dataset RBPs. The scale here signifies the number of overlapping binding sites per total number of occurrences for the independent proteins. [file Image4.JPEG]

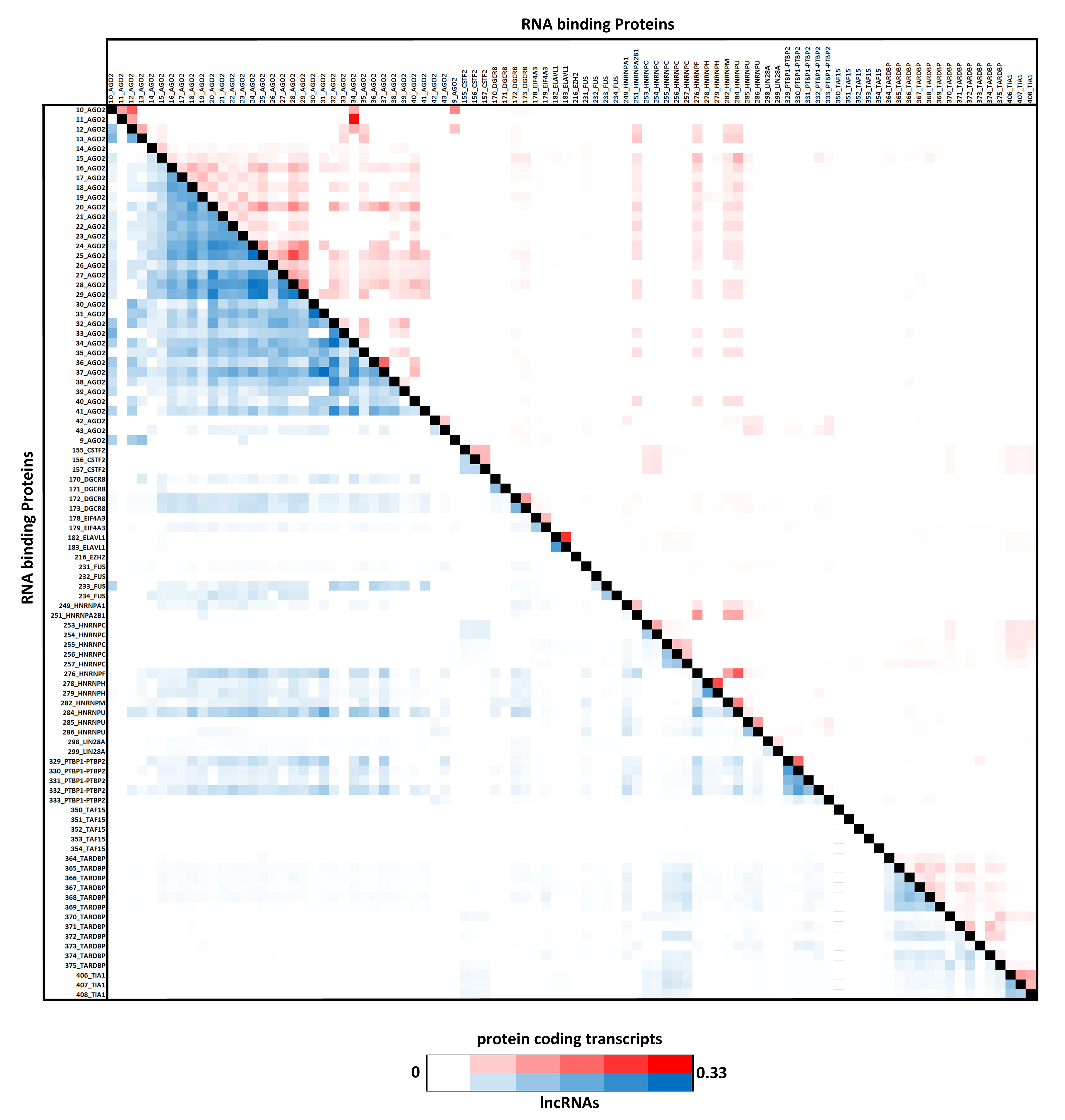

Supplement: Supplementary Figure 5 — The Heatmap depicts the combinatorial patterns of clustered protein-binding sites across lncRNAs (blue in color) and protein coding transcripts (red in color) for CLIPdb-CIMS dataset RBPs. The scale here signifies the number of overlapping binding sites per total number of occurrences for the independent proteins. [file Image5.JPEG]

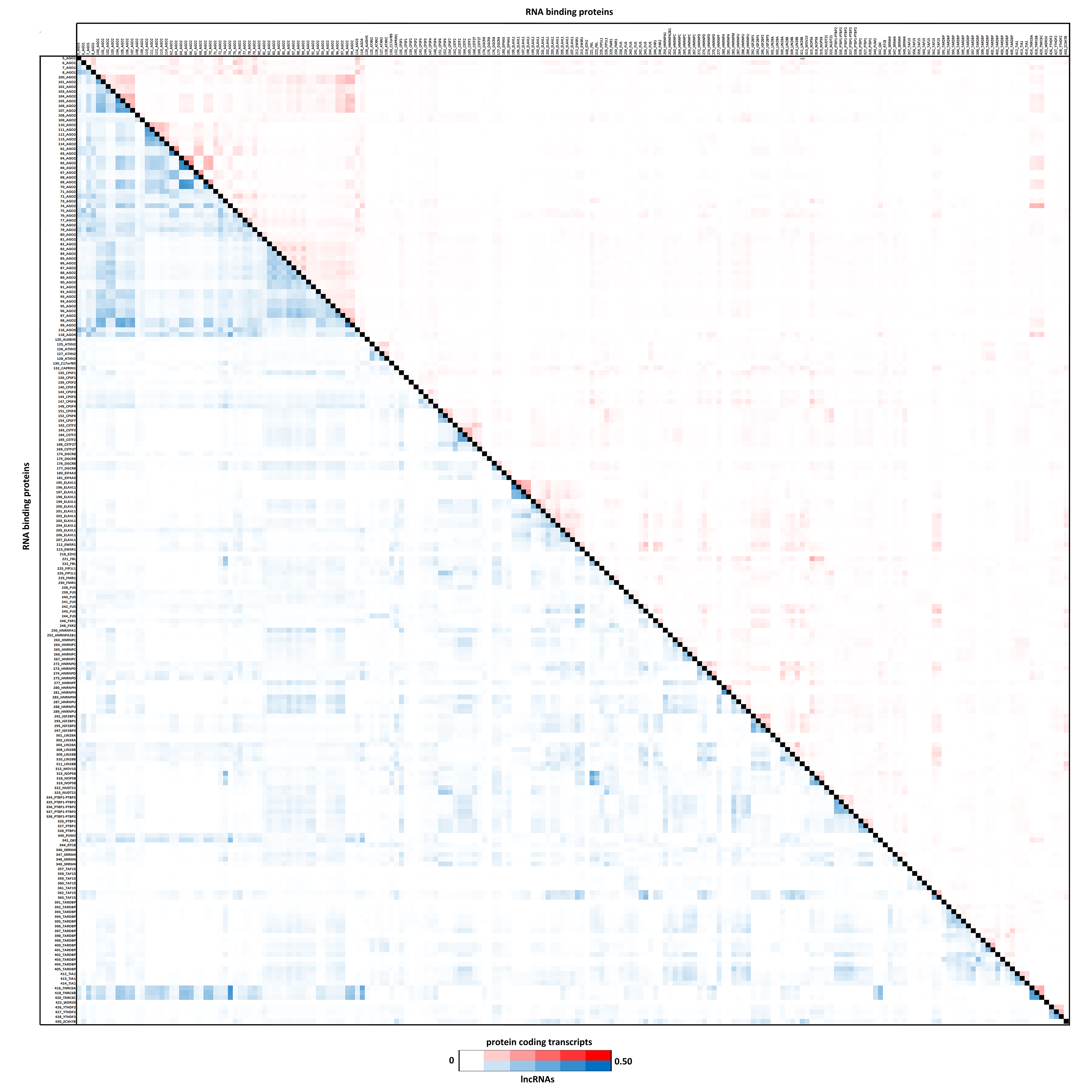

Supplement: Supplementary Figure 6 — The Heatmap depicts the combinatorial patterns of clustered protein-binding sites across lncRNAs (blue in color) and protein coding transcripts (red in color) for CLIPdb-Piranha-non-stranded dataset) RBP. The scale here signifies the number of overlapping binding sites per total number of occurrences for the independent proteins. [file Image6.JPEG]

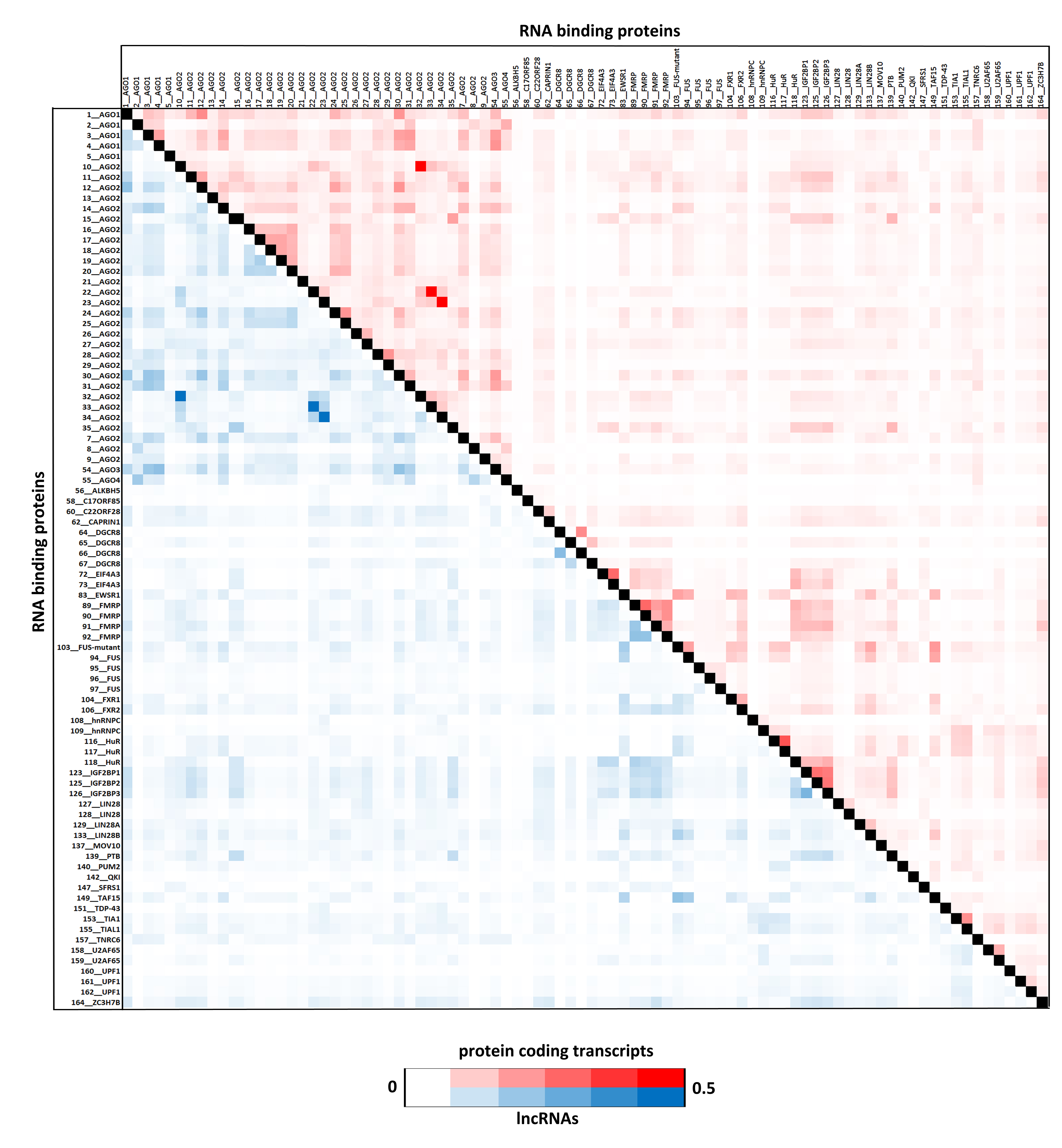

Supplement: Supplementary Figure 7 — The Heatmap depicts the combinatorial patterns of clustered protein-binding sites across lncRNAs (blue in color) and protein coding transcripts (red in color) for starBase dataset RBPs. The scale here signifies the number of overlapping binding sites per total number of occurrences for the independent proteins. [file Image7.JPEG]

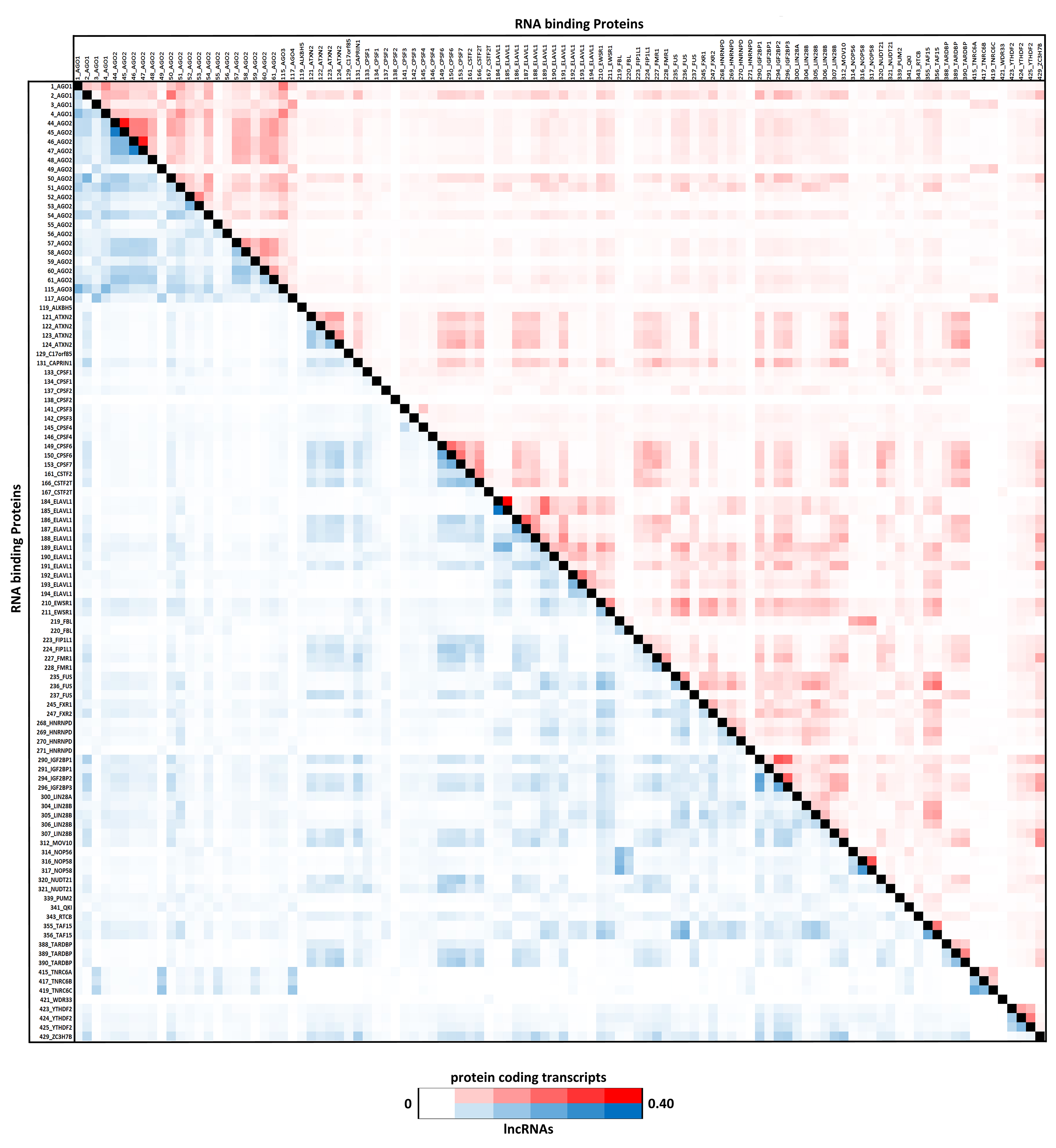

Supplement: Supplementary Figure 8 — The Heatmap depicts the combinatorial patterns of clustered protein-binding sites across lncRNAs (blue in color) and protein coding transcripts (red in color) for Clipdb-PARalyzer dataset) RBPs. The scale here signifies the number of overlapping binding sites per total number of occurrences for the independent proteins. [file Image8.JPEG]

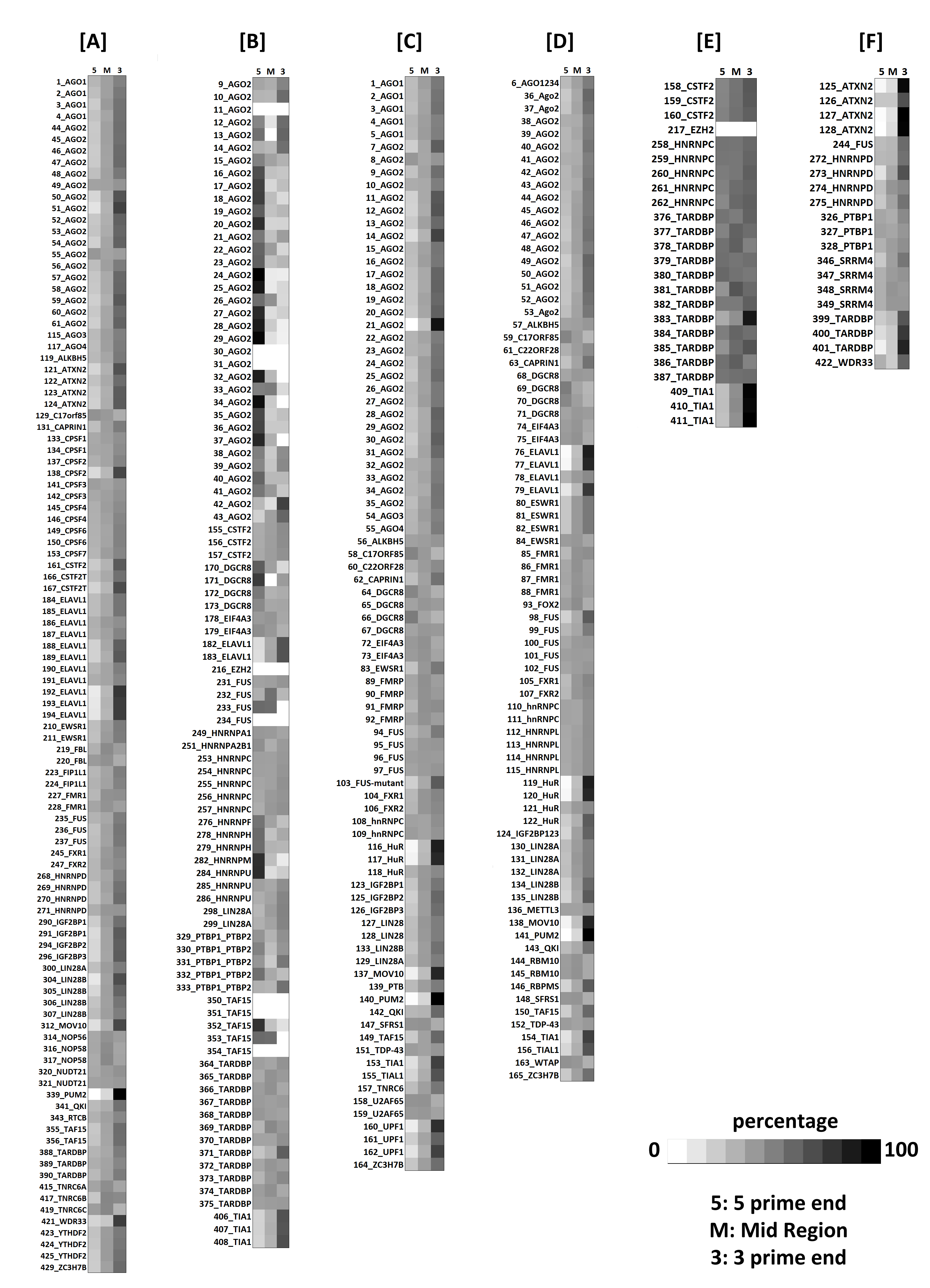

Supplement: Supplementary Figure 9 — Positional preference of protein-binding sites in lncRNAs transcripts for (A) Clipdb-PARalyzer, (B) CLIPdb-CIMS, (C) starBase, (D) doRiNA, (E) CLIPdb-CITS, and (F) CLIPdb-Piranha-stranded. [file Image9.JPEG]

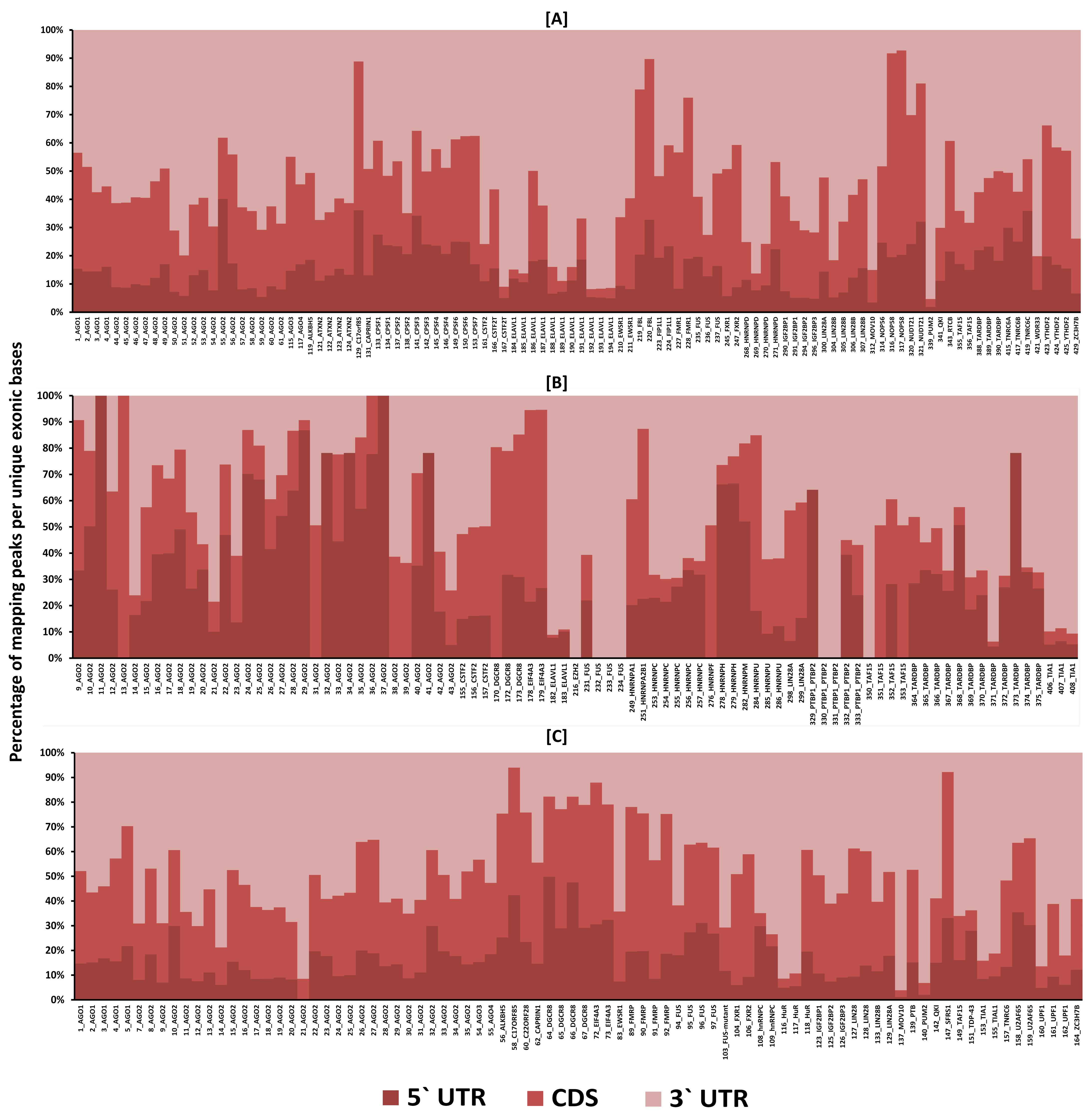

Supplement: Supplementary Figure 10 — (A) Distribution of RNA binding proteins sites from (A) Clipdb-PARalyzer, (B) CLIPdb-CIMS, and (C) starBase datasets across Refseq genes. X-axis of the graph depicts the distribution of RNA binding protein interaction sites in refseq genes and Y-axis is the frequency of binding sites. [file Image10.JPEG]

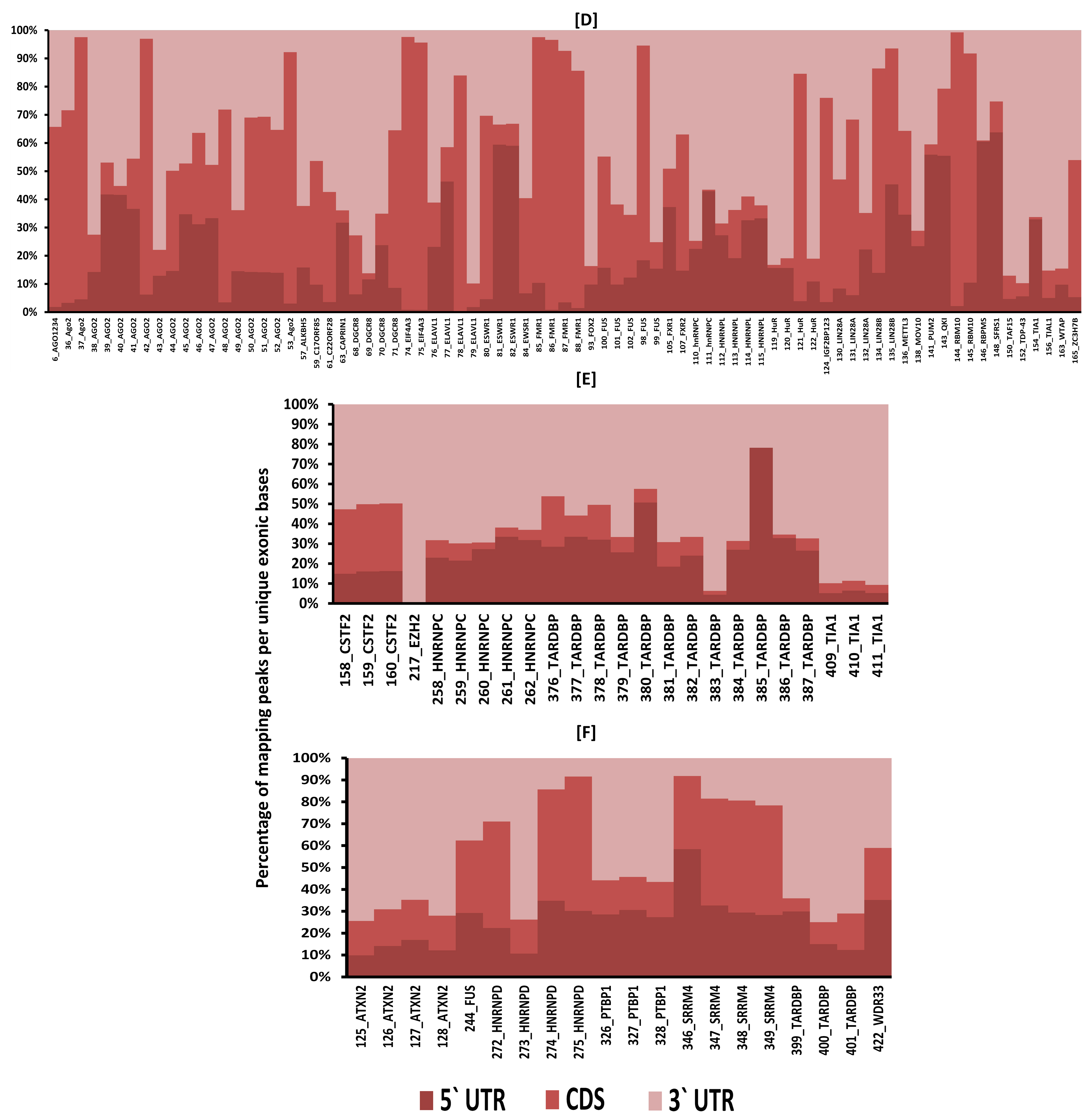

Supplement: Supplementary Figure 11 — (B) Distribution of RNA binding proteins sites from (A) doRiNA, (B) CLIPdb-CITS and (C) CLIPdb-Piranha-stranded datasets across Refseq genes. X-axis of the graph depicts the distribution of RNA binding protein interaction sites in refseq genes and Y-axis is the frequency of binding sites. [file Image11.JPEG]

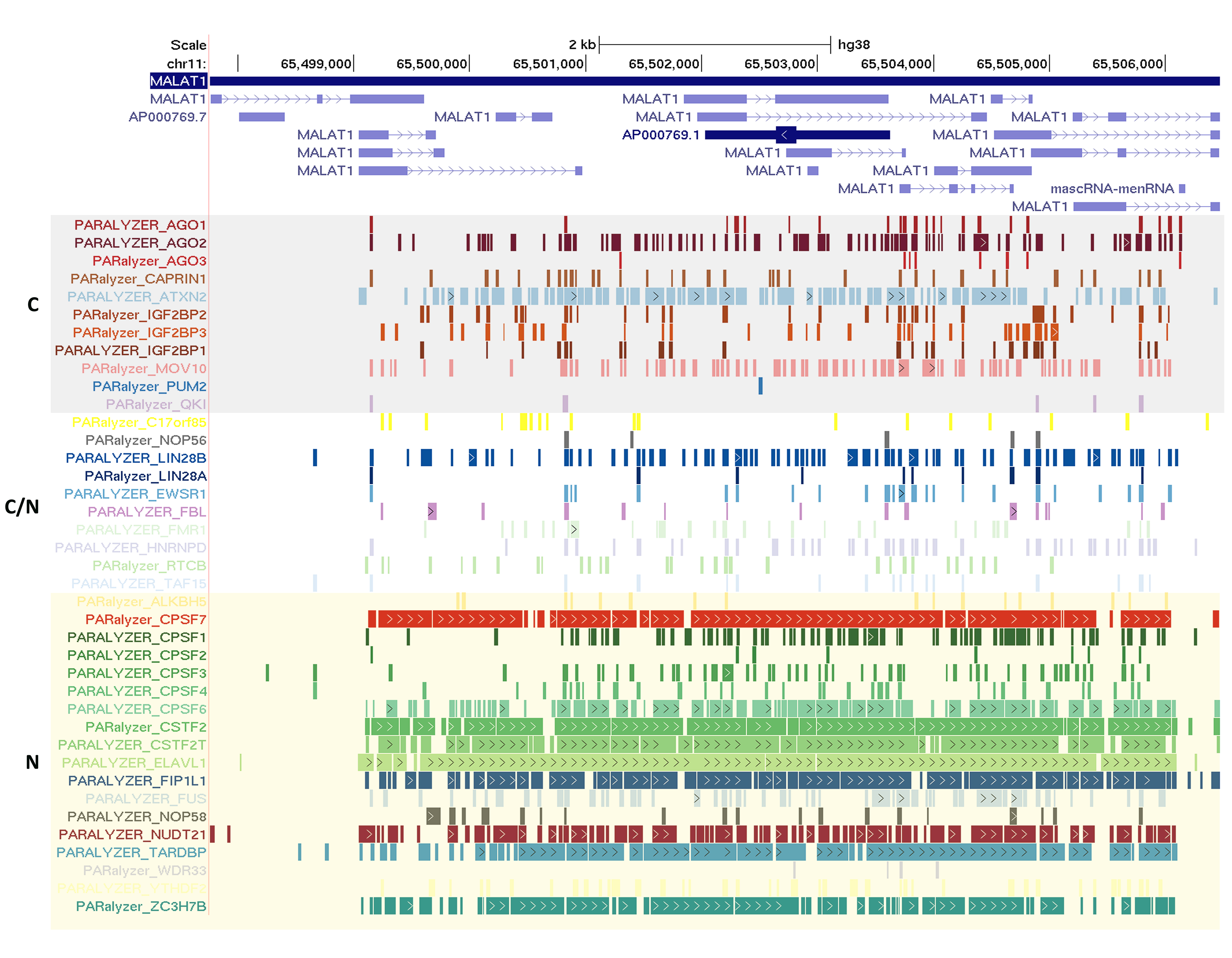

Supplement: Supplementary Figure 12 — Depiction of the mapping of RNA binding protein interaction sites from Clipdb-PARalyzer datasets across the length of MALAT1 lncRNA. The RBP highlighted in gray box are the ones generally localized to cytoplasm (C). The RBP generally localized to nucleus (N) are marked as yellow box. C/N labeled RBPs is the ones which are present in both Nucleus and Cytoplasm. [file Image12.JPEG]

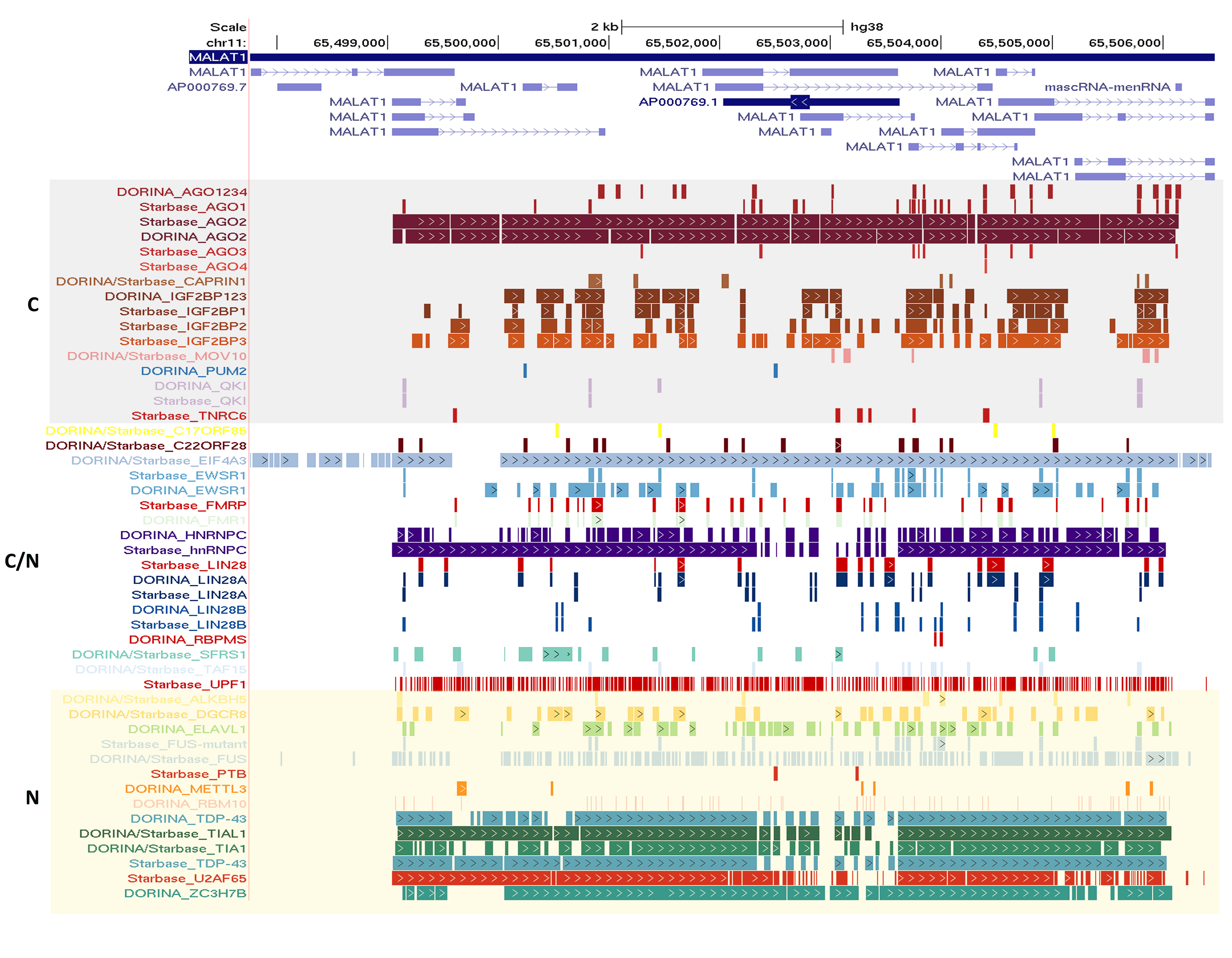

Supplement: Supplementary Figure 13 — Depiction of the mapping of RNA binding protein interaction sites from ClipDB (doRiNA and starBase datasets) across the length of MALAT1 lncRNA. The RBP highlighted in gray box are the ones generally localized to cytoplasm (C). The RBP generally localized to nucleus (N) are marked as yellow box. C/N labeled RBPs is the ones which are present in both Nucleus and Cytoplasm. [file Image13.JPEG]
