# Supplementary material for: Distinct and Modular Organization of Protein Interacting Sites in Long Non-coding RNAs
Source: Front Mol Biosci. 2018 Apr 4;5:27. doi: 10.3389/fmolb.2018.00027 (PMC5893854; doi:10.3389/fmolb.2018.00027)
Supplement: Supplementary Tables 1 — (A) Detailed list of publically available datasets derieved from Starbase and Dorina databases. (B) Detailed list of publically available datasets derieved from CLIPdb database. [file Table1.PDF]

Supplementary Table 1(A): Detailed list of publically available datasets derieved from Starbase and Dorina databases.										
**S.No**	**Source Database**	**RBP Name**	**Pubmed Id**	**Publication**	**Technique Used**	**No of peaks**	**No. of peaks after liftover (hg19to38 using CrossMap-0.2.2)**	**File name**	**Renamed To**	**Cell Line**
1	Starbase	AGO1	23446348	Memczak S, et al. Nature 2013	PAR-CLIP	82196	82219	starBase2_NAR2014_HPRO6.bed	1_Starbase_AGO1.bed	HEK293
2	Starbase	AGO1	23446348	Memczak S, et al. Nature 2013	PAR-CLIP	3453	3454	starBase2_NAR2014_HPRO7.bed	2_Starbase_AGO1.bed	HEK293
3	Starbase	AGO1	23446348	Memczak S, et al. Nature 2013	PAR-CLIP	15887	15890	starBase2_NAR2014_HPRO8.bed	3_Starbase_AGO1.bed	HEK293
4	Starbase	AGO1	20371350	Hafner M, et al. Cell 2010	PAR-CLIP	12163	12165	starBase2_NAR2014_HPTA1.bed	4_Starbase_AGO1.bed	HEK293
5	Starbase	AGO1	23622248	Helwak A, et al. Cell 2013	CLASH	26520	26531	starBase2_NAR2014_HCTA1.bed	5_Starbase_AGO1.bed	Flp_In_T_REx_293_PTH_AGO1
6	Dorina	AGO1234	20371350	Hafner, 2010	PAR-CLIP	41450	41457	PARCLIP_AGO1234_Hafner2010a_hg19.bed	6_Dorina_AGO1234.bed	HEK293
7	Starbase	AGO2	23446348	Memczak S, et al. Nature 2013	PAR-CLIP	27697	27705	starBase2_NAR2014_HPRT3.bed	7_Starbase_AGO2.bed	HEK293
8	Starbase	AGO2	20371350	Hafner M, et al. Cell 2010	PAR-CLIP	2082	2082	starBase2_NAR2014_HPTA2.bed	8_Starbase_AGO2.bed	HEK293
9	Starbase	AGO2	22012620	Lipchina I, et al. Genes Dev 2011	PAR-CLIP	9169	9172	starBase2_NAR2014_HPHES.bed	9_Starbase_AGO2.bed	hESCs_WA_09
10	Starbase	AGO2	23824327	Karginov FV, et al. Genes Dev 2013	HITS-CLIP	24041	24048	starBase2_NAR2014_HHHSA.bed	10_Starbase_AGO2.bed	293S
11	Starbase	AGO2	21572407	Kishore S, et al. Nat Methods 2011	HITS-CLIP	52812	52817	starBase2_NAR2014_HHKTA.bed	11_Starbase_AGO2.bed	HEK293
12	Starbase	AGO2	21572407	Kishore S, et al. Nat Methods 2011	PAR-CLIP	65679	65688	starBase2_NAR2014_HPKTA.bed	12_Starbase_AGO2.bed	HEK293
13	Starbase	AGO2	21572407	Kishore S, et al. Nat Methods 2011	HITS-CLIP	4103	4103	starBase2_NAR2014_HHKTB.bed	13_Starbase_AGO2.bed	HEK293
14	Starbase	AGO2	21572407	Kishore S, et al. Nat Methods 2011	PAR-CLIP	11465	11468	starBase2_NAR2014_HPKTB.bed	14_Starbase_AGO2.bed	HEK293
15	Starbase	AGO2	23313552	Xue Y, et al. Cell 2013	HITS-CLIP	169346	169365	starBase2_NAR2014_HHFCT.bed	15_Starbase_AGO2.bed	Hela
16	Starbase	AGO2	22291592	Skalsky RL, et al. PLoS Pathog 2012	PAR-CLIP	24608	24611	starBase2_NAR2014_HPSEF.bed	16_Starbase_AGO2.bed	EF3D_AGO2
17	Starbase	AGO2	22291592	Skalsky RL, et al. PLoS Pathog 2012	PAR-CLIP	8070	8069	starBase2_NAR2014_HPSLB.bed	17_Starbase_AGO2.bed	LCL_BAC
18	Starbase	AGO2	22291592	Skalsky RL, et al. PLoS Pathog 2012	PAR-CLIP	10248	10249	starBase2_NAR2014_HPSD1.bed	18_Starbase_AGO2.bed	LCL_BACD1
19	Starbase	AGO2	22291592	Skalsky RL, et al. PLoS Pathog 2012	PAR-CLIP	10959	10960	starBase2_NAR2014_HPSD3.bed	19_Starbase_AGO2.bed	LCL_BACD3
20	Starbase	AGO2	22291592	Skalsky RL, et al. PLoS Pathog 2012	PAR-CLIP	21262	21264	starBase2_NAR2014_HPSLC.bed	20_Starbase_AGO2.bed	LCL35
21	Starbase	AGO2	22473208	Riley KJ, et al. EMBO J 2012	HITS-CLIP	2773	2776	starBase2_NAR2014_HHSJI.bed	21_Starbase_AGO2.bed	Jijoye
22	Starbase	AGO2	23824327	Karginov FV, et al. Genes Dev 2013	HITS-CLIP	22506	22508	starBase2_NAR2014_HHHSE.bed	22_Starbase_AGO2.bed	293S
23	Starbase	AGO2	23824327	Karginov FV, et al. Genes Dev 2013	HITS-CLIP	4768	4769	starBase2_NAR2014_HHHSH.bed	23_Starbase_AGO2.bed	293S
24	Starbase	AGO2	22100165	Gottwein E, et al. Cell Host Microbe 2011	PAR-CLIP	43997	44003	starBase2_NAR2014_HPCB1.bed	24_Starbase_AGO2.bed	BC_1
25	Starbase	AGO2	22100165	Gottwein E, et al. Cell Host Microbe 2011	PAR-CLIP	25506	25509	starBase2_NAR2014_HPCB3.bed	25_Starbase_AGO2.bed	BC_3
26	Starbase	AGO2	22927820	Haecker I, et al. PLoS Pathog 2012	HITS-CLIP	14220	14255	starBase2_NAR2014_HHRBC.bed	26_Starbase_AGO2.bed	BC_3
27	Starbase	AGO2	22927820	Haecker I, et al. PLoS Pathog 2012	HITS-CLIP	15795	15841	starBase2_NAR2014_HHRBL.bed	27_Starbase_AGO2.bed	BCBL_1
28	Starbase	AGO2	21572407	Kishore S, et al. Nat Methods 2011	PAR-CLIP	15871	15882	starBase2_NAR2014_HPKMA.bed	28_Starbase_AGO2.bed	HEK293
29	Starbase	AGO2	21572407	Kishore S, et al. Nat Methods 2011	PAR-CLIP	8648	8656	starBase2_NAR2014_HPKMB.bed	29_Starbase_AGO2.bed	HEK293
30	Starbase	AGO2	20371350	Hafner M, et al. Cell 2010	PAR-CLIP	31805	31810	starBase2_NAR2014_HPTK1.bed	30_Starbase_AGO2.bed	HEK293
31	Starbase	AGO2	20371350	Hafner M, et al. Cell 2010	PAR-CLIP	8411	8412	starBase2_NAR2014_HPTK7.bed	31_Starbase_AGO2.bed	HEK293
32	Starbase	AGO2	23824327	Karginov FV, et al. Genes Dev 2013	HITS-CLIP	24041	24048	starBase2_NAR2014_HHHNA.bed	32_Starbase_AGO2.bed	293S
33	Starbase	AGO2	23824327	Karginov FV, et al. Genes Dev 2013	HITS-CLIP	22506	22508	starBase2_NAR2014_HHHNE.bed	33_Starbase_AGO2.bed	293S
34	Starbase	AGO2	23824327	Karginov FV, et al. Genes Dev 2013	HITS-CLIP	4768	4769	starBase2_NAR2014_HHHNH.bed	34_Starbase_AGO2.bed	293S
35	Starbase	AGO2	23313552	Xue Y, et al. Cell 2013	HITS-CLIP	155845	155864	starBase2_NAR2014_HHFKP.bed	35_Starbase_AGO2.bed	Hela
36	Dorina	Ago2	21572407	Kishore, 2011	CLIP-Seq	52767	52779	CLIPSeq_Ago2_Kishore2011a_hg19.bed	36_Dorina_Ago2.bed	HEK293
37	Dorina	Ago2	21572407	Kishore, 2011	PAR-CLIP	86500	86536	PARCLIP_Ago2_Kishore2011b_hg19.bed	37_Dorina_Ago2.bed	HEK293
38	Dorina	AGO2	23824327	Karginov, 2013	HITS-CLIP	24040	24047	HITSCLIP_AGO2Karginov2013a_hg19.bed	38_Dorina_AGO2.bed	293S

39	Dorina	AGO2	23824327	Karginov, 2013	HITS-CLIP	22505	22507	HITSCLIP_AGO2Karginov2013c_hg19.bed	39_Dorina_AGO2.bed	293S
40	Dorina	AGO2	23824327	Karginov, 2013	HITS-CLIP	4767	4768	HITSCLIP_AGO2Karginov2013b_hg19.bed	40_Dorina_AGO2.bed	293S
41	Dorina	AGO2	23824327	Karginov, 2013	HITS-CLIP	24040	24047	HITSCLIP_AGO2Karginov2013d_hg19.bed	41_Dorina_AGO2.bed	293S
42	Dorina	AGO2	23824327	Karginov, 2013	HITS-CLIP	22505	22507	HITSCLIP_AGO2Karginov2013f_hg19.bed	42_Dorina_AGO2.bed	293S
43	Dorina	AGO2	23824327	Karginov, 2013	HITS-CLIP	4767	4768	HITSCLIP_AGO2Karginov2013e_hg19.bed	43_Dorina_AGO2.bed	293S
44	Dorina	AGO2	22927820	Haecker, 2012	HITS-CLIP	14219	14254	HITSCLIP_AGO2Haecker2012a_hg19.bed	44_Dorina_AGO2.bed	BC_3
45	Dorina	AGO2	22927820	Haecker, 2012	HITS-CLIP	15794	15840	HITSCLIP_AGO2Haecker2012b_hg19.bed	45_Dorina_AGO2.bed	BCBL_1
46	Dorina	AGO2	22100165	Gottwein, 2011	PAR-CLIP	70133	70137	PARCLIP_AGO2Gottwein2011a_hg19.bed	46_Dorina_AGO2.bed	BC_1
47	Dorina	AGO2	22100165	Gottwein, 2011	PAR-CLIP	44120	44124	PARCLIP_AGO2Gottwein2011b_hg19.bed	47_Dorina_AGO2.bed	BC_3
48	Dorina	AGO2	22291592	Skalsky, 2012	PAR-CLIP	46915	46922	PARCLIP_AGO2Skalsky2012a_hg19.bed	48_Dorina_AGO2.bed	EF3D_AGO2
49	Dorina	AGO2	22291592	Skalsky, 2012	PAR-CLIP	43376	43378	PARCLIP_AGO2Skalsky2012b_hg19.bed	49_Dorina_AGO2.bed	LCL35
50	Dorina	AGO2	22291592	Skalsky, 2012	PAR-CLIP	12615	12614	PARCLIP_AGO2Skalsky2012c_hg19.bed	50_Dorina_AGO2.bed	LCLBAC
51	Dorina	AGO2	22291592	Skalsky, 2012	PAR-CLIP	16983	16985	PARCLIP_AGO2Skalsky2012d_hg19.bed	51_Dorina_AGO2.bed	LCLBACD1
52	Dorina	AGO2	22291592	Skalsky, 2012	PAR-CLIP	32873	32876	PARCLIP_AGO2Skalsky2012e_hg19.bed	52_Dorina_AGO2.bed	LCLBACD3
53	Dorina	Ago2	21572407	Kishore, 2011	PAR-CLIP	52042	52051	PARCLIP_Ago2MNase_Kishore2011c_hg19.bed	53_Dorina_Ago2.bed	HEK293
54	Starbase	AGO3	20371350	Hafner M, et al. Cell 2010	PAR-CLIP	20001	20006	starBase2_NAR2014_HPTA3.bed	54_Starbase_AGO3.bed	HEK293
55	Starbase	AGO4	20371350	Hafner M, et al. Cell 2010	PAR-CLIP	4397	4398	starBase2_NAR2014_HPTA4.bed	55_Starbase_AGO4.bed	HEK293
56	Starbase	ALKBH5	22681889	Baltz AG, et al. Mol Cell 2012	PAR-CLIP	1596	1597	starBase2_NAR2014_HPLAL.bed	56_Starbase_ALKBH5.bed	HEK293
57	Dorina	ALKBH5	22681889	Baltz, 2012	PAR-CLIP	1596	1597	PARCLIP_ALKBH5_Baltz2012a_hg19.bed	57_Dorina_ALKBH5.bed	HEK293
58	Starbase	C17ORF85	22681889	Baltz AG, et al. Mol Cell 2012	PAR-CLIP	2387	2389	starBase2_NAR2014_HPLC1.bed	58_Starbase_C17ORF85.bed	HEK293
59	Dorina	C17ORF85	22681889	Baltz, 2012	PAR-CLIP	2387	2389	PARCLIP_C17ORF85_Baltz2012b_hg19.bed	59_Dorina_C17ORF85.bed	HEK293
60	Starbase	C22ORF28	22681889	Baltz AG, et al. Mol Cell 2012	PAR-CLIP	11076	11078	starBase2_NAR2014_HPLC2.bed	60_Starbase_C22ORF28.bed	HEK293
61	Dorina	C22ORF28	22681889	Baltz, 2012	PAR-CLIP	11076	11078	PARCLIP_C22ORF28_Baltz2012c_hg19.bed	61_Dorina_C22ORF28.bed	HEK293
62	Starbase	CAPRIN1	22681889	Baltz AG, et al. Mol Cell 2012	PAR-CLIP	10498	10500	starBase2_NAR2014_HPLCA.bed	62_Starbase_CAPRIN1.bed	HEK293
63	Dorina	CAPRIN1	22681889	Baltz, 2012	PAR-CLIP	10498	10500	PARCLIP_CAPRIN1_Baltz2012d_hg19.bed	63_Dorina_CAPRIN1.bed	HEK293
64	Starbase	DGCR8	22796965	Macias S, et al. Nat Struct Mol Biol 2012	HITS-CLIP	58704	58714	starBase2_NAR2014_HHCD1.bed	64_Starbase_DGCR8.bed	HEK293T
65	Starbase	DGCR8	22796965	Macias S, et al. Nat Struct Mol Biol 2012	HITS-CLIP	124354	124378	starBase2_NAR2014_HHCD2.bed	65_Starbase_DGCR8.bed	HEK293T
66	Starbase	DGCR8	22796965	Macias S, et al. Nat Struct Mol Biol 2012	HITS-CLIP	33478	33485	starBase2_NAR2014_HHCT1.bed	66_Starbase_DGCR8.bed	HEK293T
67	Starbase	DGCR8	22796965	Macias S, et al. Nat Struct Mol Biol 2012	HITS-CLIP	38579	38594	starBase2_NAR2014_HHCT2.bed	67_Starbase_DGCR8.bed	HEK293T
68	Dorina	DGCR8	22796965	Macias, 2012	HITS-CLIP	38578	38593	HITSCLIP_DGCR8Macias2012d_hg19.bed	68_Dorina_DGCR8.bed	HEK293T
69	Dorina	DGCR8	22796965	Macias, 2012	HITS-CLIP	33477	33484	HITSCLIP_DGCR8Macias2012c_hg19.bed	69_Dorina_DGCR8.bed	HEK293T
70	Dorina	DGCR8	22796965	Macias, 2012	HITS-CLIP	58703	58713	HITSCLIP_DGCR8Macias2012a_hg19.bed	70_Dorina_DGCR8.bed	HEK293T
71	Dorina	DGCR8	22796965	Macias, 2012	HITS-CLIP	124353	124377	HITSCLIP_DGCR8Macias2012b_hg19.bed	71_Dorina_DGCR8.bed	HEK293T
72	Starbase	EIF4A3	23085716	Sauliere J, et al. Nat Struct Mol Biol 2012	HITS-CLIP	364659	364815	starBase2_NAR2014_HHLE1.bed	72_Starbase_EIF4A3.bed	Hela
73	Starbase	EIF4A3	23085716	Sauliere J, et al. Nat Struct Mol Biol 2012	HITS-CLIP	1262573	1262940	starBase2_NAR2014_HHLE2.bed	73_Starbase_EIF4A3.bed	Hela
74	Dorina	EIF4A3	23085716	Saulière, 2011	HITS-CLIP	1262572	1262939	HITSCLIP_EIF4A3Sauliere2012a_hg19.bed	74_Dorina_EIF4A3.bed	Hela
75	Dorina	EIF4A3	23085716	Saulière, 2011	HITS-CLIP	364658	364814	HITSCLIP_EIF4A3Sauliere2012b_hg19.bed	75_Dorina_EIF4A3.bed	Hela
76	Dorina	ELAVL1	21572407	Kishore, 2011	CLIP-Seq	9873	9880	CLIPSEQ_ELAVL1_hg19.bed	76_Dorina_ELAVL1.bed	HEK293
77	Dorina	ELAVL1	21572407	Kishore, 2011	PAR-CLIP	10778	10782	PARCLIP_ELAVL1A_hg19.bed	77_Dorina_ELAVL1.bed	HEK293
78	Dorina	ELAVL1	21572407	Kishore, 2011	PAR-CLIP	144886	144918	PARCLIP_ELAVL1MNASE_hg19.bed	78_Dorina_ELAVL1.bed	HEK293
79	Dorina	ELAVL1	21723171	Lebedeva, 2011	PAR-CLIP	32129	32146	PARCLIP_ELAVL1_Lebedeva2011_hg19.bed	79_Dorina_ELAVL1.bed	Hela
80	Dorina	EWSR1	22081015	Hoell, 2011	PAR-CLIP	19019	19024	PARCLIP_EWSR1_hg19.bed	80_Dorina_EWSR1.bed	HEK293
81	Dorina	EWSR1	22081015	Hoell, 2011	PAR-CLIP	19018	19023	PARCLIP_EWSR1Hoel2011_hg19.bed	81_Dorina_EWSR1.bed	HEK293
82	Dorina	EWSR1	22081015	Hoell, 2011	PAR-CLIP	19019	19024	PARCLIP_EWSR1_Hoell2011b_hg19.bed	82_Dorina_EWSR1.bed	HEK293
83	Starbase	EWSR1	22081015	Hoell JI, et al. Nat Struct Mol Biol 2011	PAR-CLIP	19019	19024	starBase2_NAR2014_HPHEW.bed	83_Starbase_EWSR1.bed	HEK293
84	Dorina	EWSR1	24813895	Paronetto, 2014	HITS-CLIP	82058	82077	HITSCLIP_EWSR1Paronetto2014_hg19.bed	84_Dorina_EWSR1.bed	Hela
85	Dorina	FMR1	23235829	Ascano, 2012	PAR-CLIP	121903	121908	PARCLIP_FMR1_Ascano2012a_hg19.bed	85_Dorina_FMR1.bed	HEK293

86	Dorina	FMR1	23235829	Ascano, 2012	PAR-CLIP	132219	132224	PARCLIP_FMR1_Ascano2012b_hg19.bed	86_Dorina_FMR1.bed	HEK293
87	Dorina	FMR1	23235829	Ascano, 2012	PAR-CLIP	78495	78500	PARCLIP_FMR1_Ascano2012c_hg19.bed	87_Dorina_FMR1.bed	HEK293
88	Dorina	FMR1	23235829	Ascano, 2012	PAR-CLIP	88644	88640	PARCLIP_FMR1_Ascano2012d_hg19.bed	88_Dorina_FMR1.bed	HEK293
89	Starbase	FMR1	23235829	Ascano M Jr, et al. Nature 2012	PAR-CLIP	132219	132224	starBase2_NAR2014_HPAM1.bed	89_Starbase_FMRP.bed	HEK293
90	Starbase	FMR1	23235829	Ascano M Jr, et al. Nature 2012	PAR-CLIP	88644	88640	starBase2_NAR2014_HPAM7.bed	90_Starbase_FMRP.bed	HEK293
91	Starbase	FMR1	23235829	Ascano M Jr, et al. Nature 2012	PAR-CLIP	121903	121908	starBase2_NAR2014_HPAW1.bed	91_Starbase_FMRP.bed	HEK293
92	Starbase	FMR1	23235829	Ascano M Jr, et al. Nature 2012	PAR-CLIP	78495	78500	starBase2_NAR2014_HPAW7.bed	92_Starbase_FMRP.bed	HEK293
93	Dorina	FOX2	19136955	Yeo, 2009	HITS-CLIP	3546	3569	HITSCLIP_FOX2Yeo2009_hg19.bed	93_Dorina_FOX2.bed	HUES6_hESCs
94	Starbase	FUS	22081015	Hoell JI, et al. Nat Struct Mol Biol 2011	PAR-CLIP	39983	39988	starBase2_NAR2014_HPHFW.bed	94_Starbase_FUS.bed	HEK293
95	Starbase	FUS	23389473	Nakaya T, et al. RNA 2013	HITS-CLIP	326115	325659	starBase2_NAR2014_HHMF1.bed	95_Starbase_FUS.bed	Brain_temporal_cortices
96	Starbase	FUS	23389473	Nakaya T, et al. RNA 2013	HITS-CLIP	415091	414603	starBase2_NAR2014_HHMF2.bed	96_Starbase_FUS.bed	Brain_temporal_cortices
97	Starbase	FUS	23389473	Nakaya T, et al. RNA 2013	HITS-CLIP	471590	471039	starBase2_NAR2014_HHMF3.bed	97_Starbase_FUS.bed	Brain_temporal_cortices
98	Dorina	FUS	22081015	Hoell, 2011	PAR-CLIP	14953	14953	PARCLIP_FUS_Hoell2011a_hg19.bed	98_Dorina_FUS.bed	HEK293
99	Dorina	FUS	22081015	Hoell, 2011	PAR-CLIP	39983	39988	PARCLIP_FUS_Hoell2011c_hg19.bed	99_Dorina_FUS.bed	HEK293
100	Dorina	FUS	23389473	Nakaya, 2013	HITS-CLIP	326114	325658	HITSCLIP_FUSNakaya2013c_hg19.bed	100_Dorina_FUS.bed	Brain
101	Dorina	FUS	23389473	Nakaya, 2013	HITS-CLIP	415090	414602	HITSCLIP_FUSNakaya2013d_hg19.bed	101_Dorina_FUS.bed	Brain
102	Dorina	FUS	23389473	Nakaya, 2013	HITS-CLIP	471589	471038	HITSCLIP_FUSNakaya2013e_hg19.bed	102_Dorina_FUS.bed	Brain
103	Starbase	FUS-mutant	22081015	Hoell JI, et al. Nat Struct Mol Biol 2011	PAR-CLIP	14953	14953	starBase2_NAR2014_HPHFM.bed	103_Starbase_FUS-mutant.bed	HEK293
104	Starbase	FXR1	23235829	Ascano M Jr, et al. Nature 2012	PAR-CLIP	9017	9017	starBase2_NAR2014_HPAR1.bed	104_Starbase_FXR1.bed	HEK293
105	Dorina	FXR1	23235829	Ascano, 2012	PAR-CLIP	9017	9017	PARCLIP_FXR1_Ascano2012e_hg19.bed	105_Dorina_FXR1.bed	HEK293
106	Starbase	FXR2	23235829	Ascano M Jr, et al. Nature 2012	PAR-CLIP	34521	34522	starBase2_NAR2014_HPAR2.bed	106_Starbase_FXR2.bed	HEK293
107	Dorina	FXR2	23235829	Ascano, 2012	PAR-CLIP	34521	34522	PARCLIP_FXR2_Ascano2012f_hg19.bed	107_Dorina_FXR2.bed	HEK293
108	Starbase	hnRNPC	20601959	Koenig J, et al. Nat Struct Mol Biol 2010	iCLIP	24448	24446	starBase2_NAR2014_HIUHN.bed	108_Starbase_hnRNPC.bed	Hela
109	Starbase	hnRNPC	23374342	Zarnack K, et al. Cell 2013	iCLIP	438360	438395	starBase2_NAR2014_HIUHC.bed	109_Starbase_hnRNPC.bed	Hela
110	Dorina	hnRNPC	20601959	Koenig, 2010	iCLIP	24448	24446	ICLIP_HNRNPC_hg19.bed	110_Dorina_hnRNPC.bed	Hela
111	Dorina	hnRNPC	20601959	Koenig, 2010	iCLIP	24448	24446	iCLIP_hnRNPC_koenig2010_hg19.bed	111_Dorina_hnRNPC.bed	Hela
112	Dorina	HNRNPL	24164894	Shankarling, 2014	HITS-CLIP	49020	49063	HITSCLIP_HNRNPLShankarling2014c_hg19.bed	112_Dorina_HNRNPL.bed	Jurkat
113	Dorina	HNRNPL	24164894	Shankarling, 2014	HITS-CLIP	46557	46588	HITSCLIP_HNRNPLShankarling2014d_hg19.bed	113_Dorina_HNRNPL.bed	CD4_plus
114	Dorina	HNRNPL	24164894	Shankarling, 2014	HITS-CLIP	40956	40960	HITSCLIP_HNRNPLShankarling2014a_hg19.bed	114_Dorina_HNRNPL.bed	Jurkat
115	Dorina	HNRNPL	24164894	Shankarling, 2014	HITS-CLIP	31781	31789	HITSCLIP_HNRNPLShankarling2014b_hg19.bed	115_Dorina_HNRNPL.bed	CD4_plus
116	Starbase	HuR	21572407	Kishore S, et al. Nat Methods 2011	HITS-CLIP	9873	9880	starBase2_NAR2014_HHKHT.bed	116_Starbase_HuR.bed	HEK293
117	Starbase	HuR	21572407	Kishore S, et al. Nat Methods 2011	PAR-CLIP	10778	10782	starBase2_NAR2014_HPKHT.bed	117_Starbase_HuR.bed	HEK293
118	Starbase	HuR	21572407	Kishore S, et al. Nat Methods 2011	PAR-CLIP	144886	144918	starBase2_NAR2014_HPKHM.bed	118_Starbase_HuR.bed	HEK293
119	Dorina	HuR	21572407	Kishore, 2011	CLIP-Seq	9873	9880	CLIPSeq_HuR_Kishore2011d_hg19.bed	119_Dorina_HuR.bed	HEK293
120	Dorina	HuR	21572407	Kishore, 2011	PAR-CLIP	10778	10782	PARCLIP_HuR_Kishore2011e_hg19.bed	120_Dorina_HuR.bed	HEK293
121	Dorina	HuR	21572407	Kishore, 2011	PAR-CLIP	144891	144923	PARCLIP_HuRMNase_Kishore2011f_hg19.bed	121_Dorina_HuR.bed	HEK293
122	Dorina	HuR	21723170	Mukherjee, 2011	PAR-CLIP	151468	151519	PARCLIP_HuR_mukherjee2011_hg19.bed	122_Dorina_HuR.bed	HEK293
123	Starbase	IGF2BP1	20371350	Hafner M, et al. Cell 2010	PAR-CLIP	66889	66906	starBase2_NAR2014_HPTI1.bed	123_Starbase_IGF2BP1.bed	HEK293
124	Dorina	IGF2BP123	20371350	Hafner, 2010	PAR-CLIP	43530	43549	PARCLIP_IGF2BP123_Hafner2010d_hg19.bed	124_Dorina_IGF2BP123.bed	HEK293
125	Starbase	IGF2BP2	20371350	Hafner M, et al. Cell 2010	PAR-CLIP	70484	70503	starBase2_NAR2014_HPTI2.bed	125_Starbase_IGF2BP2.bed	HEK293
126	Starbase	IGF2BP3	20371350	Hafner M, et al. Cell 2010	PAR-CLIP	105708	105736	starBase2_NAR2014_HPTI3.bed	126_Starbase_IGF2BP3.bed	HEK293
127	Starbase	LIN28	22959275	Wilbert ML, et al. Mol Cell 2012	HITS-CLIP	20147	20149	starBase2_NAR2014_HHYLH.bed	127_Starbase_LIN28.bed	H9_hESCs
128	Starbase	LIN28	22959275	Wilbert ML, et al. Mol Cell 2012	HITS-CLIP	11534	11537	starBase2_NAR2014_HHYLV.bed	128_Starbase_LIN28.bed	LIN28_V5_293
129	Starbase	LIN28A	23481595	Hafner M, et al. RNA 2013	PAR-CLIP	31723	31732	starBase2_NAR2014_HPTLA.bed	129_Starbase_LIN28A.bed	HEK293
130	Dorina	LIN28A	23481595	Hafner, 2013	PAR-CLIP	16002	16012	PARCLIP_LIN28AHafner2013_hg19.bed	130_Dorina_LIN28A.bed	HEK293
131	Dorina	LIN28A	22959275	Wilbert, 2012	HITS-CLIP	20146	20148	HITSCLIP_LIN28AWilbert2012a_hg19.bed	131_Dorina_LIN28A.bed	H9_hESCs
132	Dorina	LIN28A	22959275	Wilbert, 2012	HITS-CLIP	11533	11536	HITSCLIP_LIN28AWilbert2012b_hg19.bed	132_Dorina_LIN28A.bed	LIN28_V5_293

133	Starbase	LIN28B	23481595	Hafner M, et al. RNA 2013	PAR-CLIP	31796	31802	starBase2_NAR2014_HPTLB.bed	133_Starbase_LIN28B.bed	HEK293
134	Dorina	LIN28B	23481595	Hafner, 2013	PAR-CLIP	13115	13119	PARCLIP_LIN28BHafner2013_hg19.bed	134_Dorina_LIN28B.bed	HEK293
135	Dorina	LIN28B	23770886	Graf, 2013	PAR-CLIP	2540	2542	PARCLIP_LIN28BGraf2013_hg19.bed	135_Dorina_LIN28B.bed	HEK293
136	Dorina	METTL3	24407421	Ping, 2014	PAR-CLIP	17559	17568	PARCLIP_METTL3Ping2014_hg19.bed	136_Dorina_METTL3.bed	HEK293T
137	Starbase	MOV10	22844102	Sievers C, et al. Nucleic Acids Res 2012	PAR-CLIP	17053	17057	starBase2_NAR2014_HPPMO.bed	137_Starbase_MOV10.bed	HEK293
138	Dorina	MOV10	22844102	Sievers, 2012	PAR-CLIP	17052	17056	PARCLIP_MOV10Sievers2014_hg19.bed	138_Dorina_MOV10.bed	HEK293
139	Starbase	PTB	23313552	Xue Y, et al. Cell 2013	HITS-CLIP	308980	309018	starBase2_NAR2014_HHFPT.bed	139_Starbase_PTB.bed	Hela
140	Starbase	PUM2	20371350	Hafner M, et al. Cell 2010	PAR-CLIP	7523	7524	starBase2_NAR2014_HPTPU.bed	140_Starbase_PUM2.bed	HEK293
141	Dorina	PUM2	20371350	Hafner, 2010	PAR-CLIP	10962	10963	PARCLIP_PUM2_Hafner2010b_hg19.bed	141_Dorina_PUM2.bed	HEK293
142	Starbase	QKI	20371350	Hafner M, et al. Cell 2010	PAR-CLIP	6166	6170	starBase2_NAR2014_HPTQK.bed	142_Starbase_QKI.bed	HEK293
143	Dorina	QKI	20371350	Hafner, 2010	PAR-CLIP	12035	12037	PARCLIP_QKI_Hafner2010c_hg19.bed	143_Dorina_QKI.bed	HEK293
144	Dorina	RBM10	24000153	Wang, 2013	PAR-CLIP	240711	240702	PARCLIP_RBM10Wang2013a_hg19.bed	144_Dorina_RBM10.bed	HEK293
145	Dorina	RBM10	24000153	Wang, 2013	PAR-CLIP	218280	218271	PARCLIP_RBM10Wang2013b_hg19.bed	145_Dorina_RBM10.bed	HEK293
146	Dorina	RBPMS	24860013	Farazi, 2014	PAR-CLIP	6207	6207	PARCLIP_RBPMSFarazi2014_hg19.bed	146_Dorina_RBPMS.bed	HEK293
147	Starbase	SFRS1	19116412	Sanford JR, et al. Genome Res 2009	HITS-CLIP	23629	23674	starBase2_NAR2014_HHLSF.bed	147_Starbase_SFRS1.bed	HEK293T
148	Dorina	SFRS1	19116412	Sanford, 2009	CLIP-Seq	23629	23674	CLIPSeq_SFRS1_Sanford2009_hg19.bed	148_Dorina_SFRS1.bed	HEK293T
149	Starbase	TAF15	22081015	Hoell JI, et al. Nat Struct Mol Biol 2011	PAR-CLIP	8677	8678	starBase2_NAR2014_HPHTA.bed	149_Starbase_TAF15.bed	HEK293
150	Dorina	TAF15	22081015	Hoell, 2011	PAR-CLIP	8676	8677	PARCLIP_TAF15Hoel2011_hg19.bed	150_Dorina_TAF15.bed	HEK293
151	Starbase	TDP-43	21358640	Tollervey et al. Nat Neurosci. 2011	iCLIP	111689	111740	starBase2_NAR2014_HIUTD.bed	151_Starbase_TDP-43.bed	brain tissue_SH_SY5Y_neuroblastoma_H9_human_embryonic_stem_cells
152	Dorina	TDP-43	21358640	Tollervey, 2011	iCLIP	118703	118751	iCLIP_TDP-43_tollervey2011_hg19.bed	152_Dorina_TDP-43.bed	Brain
153	Starbase	TIA1	21048981	Wang Z, et al. PLoS Biol 2010	iCLIP	21884	21880	starBase2_NAR2014_HIUTI.bed	153_Starbase_TIA1.bed	Hela
154	Dorina	TIA1	21048981	Wang, 2010	iCLIP	21884	21880	iCLIP_TIA1_wang2010a_hg19.bed	154_Dorina_TIA1.bed	Hela
155	Starbase	TIAL1	21048981	Wang Z, et al. PLoS Biol 2010	iCLIP	51751	51753	starBase2_NAR2014_HIUTL.bed	155_Starbase_TIAL1.bed	Hela
156	Dorina	TIAL1	21048981	Wang, 2010	iCLIP	51751	51753	iCLIP_TIAL1_wang2010b_hg19.bed	156_Dorina_TIAL1.bed	Hela
157	Starbase	TNRC6	20371350	Hafner M, et al. Cell 2010	PAR-CLIP	4620	4623	starBase2_NAR2014_HPTTN.bed	157_Starbase_TNRC6.bed	HEK293
158	Starbase	U2AF65	23374342	Zarnack K, et al. Cell 2013	iCLIP	518794	518790	starBase2_NAR2014_HIUUC.bed	158_Starbase_U2AF65.bed	Hela
159	Starbase	U2AF65	23374342	Zarnack K, et al. Cell 2013	iCLIP	1122142	1122143	starBase2_NAR2014_HIUUS.bed	159_Starbase_U2AF65.bed	Hela
160	Starbase	UPF1	23832275	Zund D, et al. Nat Struct Mol Biol 2013	iCLIP	141390	141377	starBase2_NAR2014_HIMU1.bed	160_Starbase_UPF1.bed	Hela
161	Starbase	UPF1	23832275	Zund D, et al. Nat Struct Mol Biol 2013	iCLIP	590588	590552	starBase2_NAR2014_HIMP2.bed	161_Starbase_UPF1.bed	Hela
162	Starbase	UPF1	23832275	Zund D, et al. Nat Struct Mol Biol 2013	iCLIP	724809	724753	starBase2_NAR2014_HIMU2.bed	162_Starbase_UPF1.bed	Hela
163	Dorina	WTAP	24407421	Ping, 2014	PAR-CLIP	3674	3676	PARCLIP_WTAPPing2014_hg19.bed	163_Dorina_WTAP.bed	HEK293T
164	Starbase	ZC3H7B	22681889	Baltz AG, et al. Mol Cell 2012	PAR-CLIP	32798	32805	starBase2_NAR2014_HPLZC.bed	164_Starbase_ZC3H7B.bed	HEK293
165	Dorina	ZC3H7B	22681889	Baltz, 2012	PAR-CLIP	32798	32805	PARCLIP_ZC3H7B_Baltz2012e_hg19.bed	165_Dorina_ZC3H7B.bed	HEK293
