# Supplementary material for: Distinct and Modular Organization of Protein Interacting Sites in Long Non-coding RNAs
Source: Front Mol Biosci. 2018 Apr 4;5:27. doi: 10.3389/fmolb.2018.00027 (PMC5893854; doi:10.3389/fmolb.2018.00027)
Supplement: Supplementary file 15 [file Table2.PDF]

Supplementary Table 1(B): Detailed list of publically available datasets derieved from CLIPdb database.

S.No	Source Database	RBP Name	Pubmed Id	Publication	Technique Used	No of peaks	No. of peaks after liftover (hg19to38 using CrossMap-0.2.2)	File name	Renamed To	Cell Line
1	CLIPdb_PARalyzer	AGO1	20371350	Hafner, M. et al.	PAR-CLIP	8701	8701	AGO1_GSE21578-GSM545212.bed	1_CLIPdb_PARalyzer_AGO1.bed	HEK293
2	CLIPdb_PARalyzer	AGO1	23446348	Memczak, S. et al.	PAR-CLIP	60710	60720	AGO1_GSE43573-GSM1065667.bed	2_CLIPdb_PARalyzer_AGO1.bed	HEK293
3	CLIPdb_PARalyzer	AGO1	23446348	Memczak, S. et al.	PAR-CLIP	1841	1841	AGO1_GSE43573-GSM1065668.bed	3_CLIPdb_PARalyzer_AGO1.bed	HEK293
4	CLIPdb_PARalyzer	AGO1	23446348	Memczak, S. et al.	PAR-CLIP	10547	10549	AGO1_GSE43573-GSM1065669.bed	4_CLIPdb_PARalyzer_AGO1.bed	HEK293
5	CLIPdb_Piranha	AGO1	20371350	Hafner, M. et al.	PAR-CLIP	1820	1821	AGO1_GSE21578-GSM545212.bed	5_CLIPdb_Piranha_AGO1.bed	HEK293
6	CLIPdb_Piranha	AGO1	23446348	Memczak, S. et al.	PAR-CLIP	9499	9505	AGO1_GSE43573-GSM1065667.bed	6_CLIPdb_Piranha_AGO1.bed	HEK293
7	CLIPdb_Piranha	AGO1	23446348	Memczak, S. et al.	PAR-CLIP	1807	1807	AGO1_GSE43573-GSM1065668.bed	7_CLIPdb_Piranha_AGO1.bed	HEK293
8	CLIPdb_Piranha	AGO1	23446348	Memczak, S. et al.	PAR-CLIP	3450	3455	AGO1_GSE43573-GSM1065669.bed	8_CLIPdb_Piranha_AGO1.bed	HEK293
9	CLIPdb_CIMS	AGO2	22927820	Haecker, I. et al.	HITS-CLIP	148	148	AGO2_GSE41357-GSM1015450.bed	9_CLIPdb_CIMS_AGO2.bed	BC_3
10	CLIPdb_CIMS	AGO2	22927820	Haecker, I. et al.	HITS-CLIP	153	153	AGO2_GSE41357-GSM1015451.bed	10_CLIPdb_CIMS_AGO2.bed	BC_3
11	CLIPdb_CIMS	AGO2	22927820	Haecker, I. et al.	HITS-CLIP	87	87	AGO2_GSE41357-GSM1015452.bed	11_CLIPdb_CIMS_AGO2.bed	BC_3
12	CLIPdb_CIMS	AGO2	22927820	Haecker, I. et al.	HITS-CLIP	141	142	AGO2_GSE41357-GSM1015453.bed	12_CLIPdb_CIMS_AGO2.bed	BCBL_1
13	CLIPdb_CIMS	AGO2	22927820	Haecker, I. et al.	HITS-CLIP	123	123	AGO2_GSE41357-GSM1015454.bed	13_CLIPdb_CIMS_AGO2.bed	BCBL_1
14	CLIPdb_CIMS	AGO2	21572407	Kishore, S. et al.	HITS-CLIP	563	564	AGO2_GSE28865-GSM714642.bed	14_CLIPdb_CIMS_AGO2.bed	HEK293
15	CLIPdb_CIMS	AGO2	21572407	Kishore, S. et al.	HITS-CLIP	812	814	AGO2_GSE28865-GSM714643.bed	15_CLIPdb_CIMS_AGO2.bed	HEK293
16	CLIPdb_CIMS	AGO2	23824327	Karginov, F. V. & Hannon, G. J.	HITS-CLIP	375	376	AGO2_GSE44404-GSM1084040.bed	16_CLIPdb_CIMS_AGO2.bed	HEK293S
17	CLIPdb_CIMS	AGO2	23824327	Karginov, F. V. & Hannon, G. J.	HITS-CLIP	353	353	AGO2_GSE44404-GSM1084041.bed	17_CLIPdb_CIMS_AGO2.bed	HEK293S
18	CLIPdb_CIMS	AGO2	23824327	Karginov, F. V. & Hannon, G. J.	HITS-CLIP	310	310	AGO2_GSE44404-GSM1084042.bed	18_CLIPdb_CIMS_AGO2.bed	HEK293S
19	CLIPdb_CIMS	AGO2	23824327	Karginov, F. V. & Hannon, G. J.	HITS-CLIP	389	390	AGO2_GSE44404-GSM1084043.bed	19_CLIPdb_CIMS_AGO2.bed	HEK293S
20	CLIPdb_CIMS	AGO2	23824327	Karginov, F. V. & Hannon, G. J.	HITS-CLIP	168	168	AGO2_GSE44404-GSM1084044.bed	20_CLIPdb_CIMS_AGO2.bed	HEK293S
21	CLIPdb_CIMS	AGO2	23824327	Karginov, F. V. & Hannon, G. J.	HITS-CLIP	252	252	AGO2_GSE44404-GSM1084045.bed	21_CLIPdb_CIMS_AGO2.bed	HEK293S
22	CLIPdb_CIMS	AGO2	23824327	Karginov, F. V. & Hannon, G. J.	HITS-CLIP	226	226	AGO2_GSE44404-GSM1084046.bed	22_CLIPdb_CIMS_AGO2.bed	HEK293S
23	CLIPdb_CIMS	AGO2	23824327	Karginov, F. V. & Hannon, G. J.	HITS-CLIP	246	246	AGO2_GSE44404-GSM1084047.bed	23_CLIPdb_CIMS_AGO2.bed	HEK293S
24	CLIPdb_CIMS	AGO2	23824327	Karginov, F. V. & Hannon, G. J.	HITS-CLIP	288	288	AGO2_GSE44404-GSM1084064.bed	24_CLIPdb_CIMS_AGO2.bed	HEK293S
25	CLIPdb_CIMS	AGO2	23824327	Karginov, F. V. & Hannon, G. J.	HITS-CLIP	429	429	AGO2_GSE44404-GSM1084065.bed	25_CLIPdb_CIMS_AGO2.bed	HEK293S
26	CLIPdb_CIMS	AGO2	23824327	Karginov, F. V. & Hannon, G. J.	HITS-CLIP	226	226	AGO2_GSE44404-GSM1084066.bed	26_CLIPdb_CIMS_AGO2.bed	HEK293S
27	CLIPdb_CIMS	AGO2	23824327	Karginov, F. V. & Hannon, G. J.	HITS-CLIP	188	188	AGO2_GSE44404-GSM1084067.bed	27_CLIPdb_CIMS_AGO2.bed	HEK293S
28	CLIPdb_CIMS	AGO2	23824327	Karginov, F. V. & Hannon, G. J.	HITS-CLIP	294	294	AGO2_GSE44404-GSM1084068.bed	28_CLIPdb_CIMS_AGO2.bed	HEK293S
29	CLIPdb_CIMS	AGO2	23824327	Karginov, F. V. & Hannon, G. J.	HITS-CLIP	318	318	AGO2_GSE44404-GSM1084069.bed	29_CLIPdb_CIMS_AGO2.bed	HEK293S
30	CLIPdb_CIMS	AGO2	23824327	Karginov, F. V. & Hannon, G. J.	HITS-CLIP	88	88	AGO2_GSE44404-GSM1084072.bed	30_CLIPdb_CIMS_AGO2.bed	HEK293S
31	CLIPdb_CIMS	AGO2	23824327	Karginov, F. V. & Hannon, G. J.	HITS-CLIP	117	117	AGO2_GSE44404-GSM1084073.bed	31_CLIPdb_CIMS_AGO2.bed	HEK293S
32	CLIPdb_CIMS	AGO2	23824327	Karginov, F. V. & Hannon, G. J.	HITS-CLIP	79	79	AGO2_GSE44404-GSM1084074.bed	32_CLIPdb_CIMS_AGO2.bed	HEK293S
33	CLIPdb_CIMS	AGO2	23824327	Karginov, F. V. & Hannon, G. J.	HITS-CLIP	102	102	AGO2_GSE44404-GSM1084075.bed	33_CLIPdb_CIMS_AGO2.bed	HEK293S
34	CLIPdb_CIMS	AGO2	23824327	Karginov, F. V. & Hannon, G. J.	HITS-CLIP	97	97	AGO2_GSE44404-GSM1084076.bed	34_CLIPdb_CIMS_AGO2.bed	HEK293S
35	CLIPdb_CIMS	AGO2	23824327	Karginov, F. V. & Hannon, G. J.	HITS-CLIP	237	237	AGO2_GSE44404-GSM1084077.bed	35_CLIPdb_CIMS_AGO2.bed	HEK293S
36	CLIPdb_CIMS	AGO2	23824327	Karginov, F. V. & Hannon, G. J.	HITS-CLIP	98	98	AGO2_GSE44404-GSM1084078.bed	36_CLIPdb_CIMS_AGO2.bed	HEK293S
37	CLIPdb_CIMS	AGO2	23824327	Karginov, F. V. & Hannon, G. J.	HITS-CLIP	78	78	AGO2_GSE44404-GSM1084079.bed	37_CLIPdb_CIMS_AGO2.bed	HEK293S
38	CLIPdb_CIMS	AGO2	23824327	Karginov, F. V. & Hannon, G. J.	HITS-CLIP	139	139	AGO2_GSE44404-GSM1084080.bed	38_CLIPdb_CIMS_AGO2.bed	HEK293S

39	CLIPdb_CIMS	AGO2	23824327	Karginov, F. V. & Hannon, G. J.	HITS-CLIP	96	96	AGO2_GSE44404-GSM1084081.bed	39_CLIPdb_CIMS_AGO2.bed	HEK293S
40	CLIPdb_CIMS	AGO2	23824327	Karginov, F. V. & Hannon, G. J.	HITS-CLIP	240	240	AGO2_GSE44404-GSM1084082.bed	40_CLIPdb_CIMS_AGO2.bed	HEK293S
41	CLIPdb_CIMS	AGO2	23824327	Karginov, F. V. & Hannon, G. J.	HITS-CLIP	95	95	AGO2_GSE44404-GSM1084083.bed	41_CLIPdb_CIMS_AGO2.bed	HEK293S
42	CLIPdb_CIMS	AGO2	23313552	Xue, Y. et al.	HITS-CLIP	110	111	AGO2_GSE42701-GSM1048187.bed	42_CLIPdb_CIMS_AGO2.bed	HeLa
43	CLIPdb_CIMS	AGO2	23313552	Xue, Y. et al.	HITS-CLIP	270	271	AGO2_GSE42701-GSM1048188.bed	43_CLIPdb_CIMS_AGO2.bed	HeLa
44	CLIPdb_PARalyzer	AGO2	22100165	Gottwein, E. et al.	PAR-CLIP	17763	17763	AGO2_GSE32113-GSM796037.bed	44_CLIPdb_PARalyzer_AGO2.bed	BC_1
45	CLIPdb_PARalyzer	AGO2	22100165	Gottwein, E. et al.	PAR-CLIP	18092	18092	AGO2_GSE32113-GSM796038.bed	45_CLIPdb_PARalyzer_AGO2.bed	BC_1
46	CLIPdb_PARalyzer	AGO2	22100165	Gottwein, E. et al.	PAR-CLIP	9377	9378	AGO2_GSE32113-GSM796039.bed	46_CLIPdb_PARalyzer_AGO2.bed	BC_3
47	CLIPdb_PARalyzer	AGO2	22100165	Gottwein, E. et al.	PAR-CLIP	9729	9730	AGO2_GSE32113-GSM796040.bed	47_CLIPdb_PARalyzer_AGO2.bed	BC_3
48	CLIPdb_PARalyzer	AGO2	22291592	Skalsky, R. L. et al.	PAR-CLIP	19631	19634	AGO2_GSE41437-GSM1020021.bed	48_CLIPdb_PARalyzer_AGO2.bed	EF3D_AGO2
49	CLIPdb_PARalyzer	AGO2	20371350	Hafner, M. et al.	PAR-CLIP	1036	1036	AGO2_GSE21578-GSM545213.bed	49_CLIPdb_PARalyzer_AGO2.bed	HEK293
50	CLIPdb_PARalyzer	AGO2	21572407	Kishore, S. et al.	PAR-CLIP	51090	51094	AGO2_GSE28865-GSM714644.bed	50_CLIPdb_PARalyzer_AGO2.bed	HEK293
51	CLIPdb_PARalyzer	AGO2	21572407	Kishore, S. et al.	PAR-CLIP	8208	8209	AGO2_GSE28865-GSM714645.bed	51_CLIPdb_PARalyzer_AGO2.bed	HEK293
52	CLIPdb_PARalyzer	AGO2	21572407	Kishore, S. et al.	PAR-CLIP	11638	11643	AGO2_GSE28865-GSM714646.bed	52_CLIPdb_PARalyzer_AGO2.bed	HEK293
53	CLIPdb_PARalyzer	AGO2	21572407	Kishore, S. et al.	PAR-CLIP	5344	5347	AGO2_GSE28865-GSM714647.bed	53_CLIPdb_PARalyzer_AGO2.bed	HEK293
54	CLIPdb_PARalyzer	AGO2	23446348	Memczak, S. et al.	PAR-CLIP	21377	21381	AGO2_GSE43573-GSM1065670.bed	54_CLIPdb_PARalyzer_AGO2.bed	HEK293
55	CLIPdb_PARalyzer	AGO2	21572407	Kishore, S. et al.	PAR-CLIP	3534	3536	AGO2_GSE43666-GSM1067869.bed	55_CLIPdb_PARalyzer_AGO2.bed	HEK293
56	CLIPdb_PARalyzer	AGO2	21572407	Kishore, S. et al.	PAR-CLIP	8459	8463	AGO2_GSE43666-GSM1067870.bed	56_CLIPdb_PARalyzer_AGO2.bed	HEK293
57	CLIPdb_PARalyzer	AGO2	22291592	Skalsky, R. L. et al.	PAR-CLIP	6656	6655	AGO2_GSE41437-GSM1020023.bed	57_CLIPdb_PARalyzer_AGO2.bed	LCL_BAC
58	CLIPdb_PARalyzer	AGO2	22291592	Skalsky, R. L. et al.	PAR-CLIP	8401	8401	AGO2_GSE41437-GSM1020024.bed	58_CLIPdb_PARalyzer_AGO2.bed	LCL_BAC_D1
59	CLIPdb_PARalyzer	AGO2	22291592	Skalsky, R. L. et al.	PAR-CLIP	1150	1150	AGO2_GSE46611-GSM1133252.bed	59_CLIPdb_PARalyzer_AGO2.bed	LCL_BAC_D2
60	CLIPdb_PARalyzer	AGO2	22291592	Skalsky, R. L. et al.	PAR-CLIP	8751	8752	AGO2_GSE41437-GSM1020025.bed	60_CLIPdb_PARalyzer_AGO2.bed	LCL_BAC_D3
61	CLIPdb_PARalyzer	AGO2	22291592	Skalsky, R. L. et al.	PAR-CLIP	17231	17231	AGO2_GSE41437-GSM1020022.bed	61_CLIPdb_PARalyzer_AGO2.bed	LCL35
62	CLIPdb_Piranha	AGO2	22100165	Gottwein, E. et al.	PAR-CLIP	2193	2193	AGO2_GSE32113-GSM796037.bed	62_CLIPdb_Piranha_AGO2.bed	BC_1
63	CLIPdb_Piranha	AGO2	22100165	Gottwein, E. et al.	PAR-CLIP	2257	2257	AGO2_GSE32113-GSM796038.bed	63_CLIPdb_Piranha_AGO2.bed	BC_1
64	CLIPdb_Piranha	AGO2	22927820	Haecker, I. et al.	HITS-CLIP	248	248	AGO2_GSE41357-GSM1015450.bed	64_CLIPdb_Piranha_AGO2.bed	BC_3
65	CLIPdb_Piranha	AGO2	22927820	Haecker, I. et al.	HITS-CLIP	229	229	AGO2_GSE41357-GSM1015451.bed	65_CLIPdb_Piranha_AGO2.bed	BC_3
66	CLIPdb_Piranha	AGO2	22927820	Haecker, I. et al.	HITS-CLIP	201	201	AGO2_GSE41357-GSM1015452.bed	66_CLIPdb_Piranha_AGO2.bed	BC_3
67	CLIPdb_Piranha	AGO2	22100165	Gottwein, E. et al.	PAR-CLIP	1358	1358	AGO2_GSE32113-GSM796039.bed	67_CLIPdb_Piranha_AGO2.bed	BC_3
68	CLIPdb_Piranha	AGO2	22100165	Gottwein, E. et al.	PAR-CLIP	1458	1458	AGO2_GSE32113-GSM796040.bed	68_CLIPdb_Piranha_AGO2.bed	BC_3
69	CLIPdb_Piranha	AGO2	22927820	Haecker, I. et al.	HITS-CLIP	325	325	AGO2_GSE41357-GSM1015453.bed	69_CLIPdb_Piranha_AGO2.bed	BCBL_1
70	CLIPdb_Piranha	AGO2	22927820	Haecker, I. et al.	HITS-CLIP	283	283	AGO2_GSE41357-GSM1015454.bed	70_CLIPdb_Piranha_AGO2.bed	BCBL_1
71	CLIPdb_Piranha	AGO2	22291592	Skalsky, R. L. et al.	PAR-CLIP	1307	1308	AGO2_GSE41437-GSM1020021.bed	71_CLIPdb_Piranha_AGO2.bed	EF3D_AGO2
72	CLIPdb_Piranha	AGO2	21572407	Kishore, S. et al.	HITS-CLIP	2730	2731	AGO2_GSE28865-GSM714642.bed	72_CLIPdb_Piranha_AGO2.bed	HEK293
73	CLIPdb_Piranha	AGO2	21572407	Kishore, S. et al.	HITS-CLIP	1041	1043	AGO2_GSE28865-GSM714643.bed	73_CLIPdb_Piranha_AGO2.bed	HEK293
74	CLIPdb_Piranha	AGO2	20371350	Hafner, M. et al.	PAR-CLIP	552	552	AGO2_GSE21578-GSM545213.bed	74_CLIPdb_Piranha_AGO2.bed	HEK293
75	CLIPdb_Piranha	AGO2	21572407	Kishore, S. et al.	PAR-CLIP	7728	7731	AGO2_GSE28865-GSM714644.bed	75_CLIPdb_Piranha_AGO2.bed	HEK293
76	CLIPdb_Piranha	AGO2	21572407	Kishore, S. et al.	PAR-CLIP	3413	3414	AGO2_GSE28865-GSM714645.bed	76_CLIPdb_Piranha_AGO2.bed	HEK293
77	CLIPdb_Piranha	AGO2	21572407	Kishore, S. et al.	PAR-CLIP	3726	3731	AGO2_GSE28865-GSM714646.bed	77_CLIPdb_Piranha_AGO2.bed	HEK293
78	CLIPdb_Piranha	AGO2	21572407	Kishore, S. et al.	PAR-CLIP	2159	2163	AGO2_GSE28865-GSM714647.bed	78_CLIPdb_Piranha_AGO2.bed	HEK293
79	CLIPdb_Piranha	AGO2	23446348	Memczak, S. et al.	PAR-CLIP	1932	1932	AGO2_GSE43573-GSM1065670.bed	79_CLIPdb_Piranha_AGO2.bed	HEK293
80	CLIPdb_Piranha	AGO2	21572407	Kishore, S. et al.	PAR-CLIP	3348	3351	AGO2_GSE43666-GSM1067869.bed	80_CLIPdb_Piranha_AGO2.bed	HEK293
81	CLIPdb_Piranha	AGO2	21572407	Kishore, S. et al.	PAR-CLIP	6995	7003	AGO2_GSE43666-GSM1067870.bed	81_CLIPdb_Piranha_AGO2.bed	HEK293
82	CLIPdb_Piranha	AGO2	23824327	Karginov, F. V. & Hannon, G. J.	HITS-CLIP	8019	8023	AGO2_GSE44404-GSM1084040.bed	82_CLIPdb_Piranha_AGO2.bed	HEK293S
83	CLIPdb_Piranha	AGO2	23824327	Karginov, F. V. & Hannon, G. J.	HITS-CLIP	5478	5480	AGO2_GSE44404-GSM1084041.bed	83_CLIPdb_Piranha_AGO2.bed	HEK293S
84	CLIPdb_Piranha	AGO2	23824327	Karginov, F. V. & Hannon, G. J.	HITS-CLIP	7746	7749	AGO2_GSE44404-GSM1084042.bed	84_CLIPdb_Piranha_AGO2.bed	HEK293S
85	CLIPdb_Piranha	AGO2	23824327	Karginov, F. V. & Hannon, G. J.	HITS-CLIP	13160	13166	AGO2_GSE44404-GSM1084043.bed	85_CLIPdb_Piranha_AGO2.bed	HEK293S

86	CLIPdb_Piranha	AGO2	23824327	Karginov, F. V. & Hannon, G. J.	HITS-CLIP	5536	5540	AGO2_GSE44404-GSM1084044.bed	86_CLIPdb_Piranha_AGO2.bed	HEK293S
87	CLIPdb_Piranha	AGO2	23824327	Karginov, F. V. & Hannon, G. J.	HITS-CLIP	4628	4629	AGO2_GSE44404-GSM1084045.bed	87_CLIPdb_Piranha_AGO2.bed	HEK293S
88	CLIPdb_Piranha	AGO2	23824327	Karginov, F. V. & Hannon, G. J.	HITS-CLIP	3879	3880	AGO2_GSE44404-GSM1084046.bed	88_CLIPdb_Piranha_AGO2.bed	HEK293S
89	CLIPdb_Piranha	AGO2	23824327	Karginov, F. V. & Hannon, G. J.	HITS-CLIP	3869	3870	AGO2_GSE44404-GSM1084047.bed	89_CLIPdb_Piranha_AGO2.bed	HEK293S
90	CLIPdb_Piranha	AGO2	23824327	Karginov, F. V. & Hannon, G. J.	HITS-CLIP	10845	10848	AGO2_GSE44404-GSM1084064.bed	90_CLIPdb_Piranha_AGO2.bed	HEK293S
91	CLIPdb_Piranha	AGO2	23824327	Karginov, F. V. & Hannon, G. J.	HITS-CLIP	13525	13528	AGO2_GSE44404-GSM1084065.bed	91_CLIPdb_Piranha_AGO2.bed	HEK293S
92	CLIPdb_Piranha	AGO2	23824327	Karginov, F. V. & Hannon, G. J.	HITS-CLIP	1334	1334	AGO2_GSE44404-GSM1084066.bed	92_CLIPdb_Piranha_AGO2.bed	HEK293S
93	CLIPdb_Piranha	AGO2	23824327	Karginov, F. V. & Hannon, G. J.	HITS-CLIP	1236	1236	AGO2_GSE44404-GSM1084067.bed	93_CLIPdb_Piranha_AGO2.bed	HEK293S
94	CLIPdb_Piranha	AGO2	23824327	Karginov, F. V. & Hannon, G. J.	HITS-CLIP	3585	3587	AGO2_GSE44404-GSM1084068.bed	94_CLIPdb_Piranha_AGO2.bed	HEK293S
95	CLIPdb_Piranha	AGO2	23824327	Karginov, F. V. & Hannon, G. J.	HITS-CLIP	3780	3781	AGO2_GSE44404-GSM1084069.bed	95_CLIPdb_Piranha_AGO2.bed	HEK293S
96	CLIPdb_Piranha	AGO2	23824327	Karginov, F. V. & Hannon, G. J.	HITS-CLIP	2812	2812	AGO2_GSE44404-GSM1084072.bed	96_CLIPdb_Piranha_AGO2.bed	HEK293S
97	CLIPdb_Piranha	AGO2	23824327	Karginov, F. V. & Hannon, G. J.	HITS-CLIP	2971	2973	AGO2_GSE44404-GSM1084073.bed	97_CLIPdb_Piranha_AGO2.bed	HEK293S
98	CLIPdb_Piranha	AGO2	23824327	Karginov, F. V. & Hannon, G. J.	HITS-CLIP	894	894	AGO2_GSE44404-GSM1084074.bed	98_CLIPdb_Piranha_AGO2.bed	HEK293S
99	CLIPdb_Piranha	AGO2	23824327	Karginov, F. V. & Hannon, G. J.	HITS-CLIP	742	741	AGO2_GSE44404-GSM1084075.bed	99_CLIPdb_Piranha_AGO2.bed	HEK293S
100	CLIPdb_Piranha	AGO2	23824327	Karginov, F. V. & Hannon, G. J.	HITS-CLIP	1763	1762	AGO2_GSE44404-GSM1084076.bed	100_CLIPdb_Piranha_AGO2.bed	HEK293S
101	CLIPdb_Piranha	AGO2	23824327	Karginov, F. V. & Hannon, G. J.	HITS-CLIP	1532	1531	AGO2_GSE44404-GSM1084077.bed	101_CLIPdb_Piranha_AGO2.bed	HEK293S
102	CLIPdb_Piranha	AGO2	23824327	Karginov, F. V. & Hannon, G. J.	HITS-CLIP	2833	2833	AGO2_GSE44404-GSM1084078.bed	102_CLIPdb_Piranha_AGO2.bed	HEK293S
103	CLIPdb_Piranha	AGO2	23824327	Karginov, F. V. & Hannon, G. J.	HITS-CLIP	3823	3824	AGO2_GSE44404-GSM1084079.bed	103_CLIPdb_Piranha_AGO2.bed	HEK293S
104	CLIPdb_Piranha	AGO2	23824327	Karginov, F. V. & Hannon, G. J.	HITS-CLIP	606	606	AGO2_GSE44404-GSM1084080.bed	104_CLIPdb_Piranha_AGO2.bed	HEK293S
105	CLIPdb_Piranha	AGO2	23824327	Karginov, F. V. & Hannon, G. J.	HITS-CLIP	653	653	AGO2_GSE44404-GSM1084081.bed	105_CLIPdb_Piranha_AGO2.bed	HEK293S
106	CLIPdb_Piranha	AGO2	23824327	Karginov, F. V. & Hannon, G. J.	HITS-CLIP	1230	1231	AGO2_GSE44404-GSM1084082.bed	106_CLIPdb_Piranha_AGO2.bed	HEK293S
107	CLIPdb_Piranha	AGO2	23824327	Karginov, F. V. & Hannon, G. J.	HITS-CLIP	1032	1032	AGO2_GSE44404-GSM1084083.bed	107_CLIPdb_Piranha_AGO2.bed	HEK293S
108	CLIPdb_Piranha	AGO2	23313552	Xue, Y. et al.	HITS-CLIP	7329	7330	AGO2_GSE42701-GSM1048187.bed	108_CLIPdb_Piranha_AGO2.bed	HeLa
109	CLIPdb_Piranha	AGO2	23313552	Xue, Y. et al.	HITS-CLIP	1978	1979	AGO2_GSE42701-GSM1048188.bed	109_CLIPdb_Piranha_AGO2.bed	HeLa
110	CLIPdb_Piranha	AGO2	22291592	Skalsky, R. L. et al.	PAR-CLIP	233	233	AGO2_GSE41437-GSM1020023.bed	110_CLIPdb_Piranha_AGO2.bed	LCL_BAC
111	CLIPdb_Piranha	AGO2	22291592	Skalsky, R. L. et al.	PAR-CLIP	201	201	AGO2_GSE41437-GSM1020024.bed	111_CLIPdb_Piranha_AGO2.bed	LCL_BAC_D1
112	CLIPdb_Piranha	AGO2	22291592	Skalsky, R. L. et al.	PAR-CLIP	241	241	AGO2_GSE46611-GSM1133252.bed	112_CLIPdb_Piranha_AGO2.bed	LCL_BAC_D2
113	CLIPdb_Piranha	AGO2	22291592	Skalsky, R. L. et al.	PAR-CLIP	595	595	AGO2_GSE41437-GSM1020025.bed	113_CLIPdb_Piranha_AGO2.bed	LCL_BAC_D3
114	CLIPdb_Piranha	AGO2	22291592	Skalsky, R. L. et al.	PAR-CLIP	709	709	AGO2_GSE41437-GSM1020022.bed	114_CLIPdb_Piranha_AGO2.bed	LCL35
115	CLIPdb_PARalyzer	AGO3	20371350	Hafner, M. et al.	PAR-CLIP	12437	12438	AGO3_GSE21578-GSM545214.bed	115_CLIPdb_PARalyzer_AGO3.bed	HEK293
116	CLIPdb_Piranha	AGO3	20371350	Hafner, M. et al.	PAR-CLIP	3171	3173	AGO3_GSE21578-GSM545214.bed	116_CLIPdb_Piranha_AGO3.bed	HEK293
117	CLIPdb_PARalyzer	AGO4	20371350	Hafner, M. et al.	PAR-CLIP	1820	1820	AGO4_GSE21578-GSM545215.bed	117_CLIPdb_PARalyzer_AGO4.bed	HEK293
118	CLIPdb_Piranha	AGO4	20371350	Hafner, M. et al.	PAR-CLIP	1341	1342	AGO4_GSE21578-GSM545215.bed	118_CLIPdb_Piranha_AGO4.bed	HEK293
119	CLIPdb_PARalyzer	ALKBH5	22681889	Baltz, A. G. et al.	PAR-CLIP	14004	14007	ALKBH5_GSE38201-GSM936506.bed	119_CLIPdb_PARalyzer_ALKBH5.bed	HEK293
120	CLIPdb_Piranha	ALKBH5	22681889	Baltz, A. G. et al.	PAR-CLIP	1128	1129	ALKBH5_GSE38201-GSM936506.bed	120_CLIPdb_Piranha_ALKBH5.bed	HEK293
121	CLIPdb_PARalyzer	ATXN2	24954906	Kawahara, Y. et al.	PAR-CLIP	79438	79459	ATXN2_DRA001158-DRS012388.bed	121_CLIPdb_PARalyzer_ATXN2.bed	HEK293T
122	CLIPdb_PARalyzer	ATXN2	24954906	Kawahara, Y. et al.	PAR-CLIP	44409	44417	ATXN2_DRA001158-DRS012389.bed	122_CLIPdb_PARalyzer_ATXN2.bed	HEK293T
123	CLIPdb_PARalyzer	ATXN2	24954906	Kawahara, Y. et al.	PAR-CLIP	114145	114160	ATXN2_DRA001158-DRS012390.bed	123_CLIPdb_PARalyzer_ATXN2.bed	HEK293T
124	CLIPdb_PARalyzer	ATXN2	24954906	Kawahara, Y. et al.	PAR-CLIP	185015	185042	ATXN2_DRA001158-DRS012391.bed	124_CLIPdb_PARalyzer_ATXN2.bed	HEK293T
125	CLIPdb_Piranha	ATXN2	24954906	Kawahara, Y. et al.	PAR-CLIP	1730	1732	ATXN2_DRA001158-DRS012388.bed	125_CLIPdb_Piranha_ATXN2.bed	HEK293T
126	CLIPdb_Piranha	ATXN2	24954906	Kawahara, Y. et al.	PAR-CLIP	806	807	ATXN2_DRA001158-DRS012389.bed	126_CLIPdb_Piranha_ATXN2.bed	HEK293T
127	CLIPdb_Piranha	ATXN2	24954906	Kawahara, Y. et al.	PAR-CLIP	2421	2422	ATXN2_DRA001158-DRS012390.bed	127_CLIPdb_Piranha_ATXN2.bed	HEK293T
128	CLIPdb_Piranha	ATXN2	24954906	Kawahara, Y. et al.	PAR-CLIP	3296	3299	ATXN2_DRA001158-DRS012391.bed	128_CLIPdb_Piranha_ATXN2.bed	HEK293T
129	CLIPdb_PARalyzer	C17orf85	22681889	Baltz, A. G. et al.	PAR-CLIP	25567	25570	C17orf85_GSE38201-GSM936507.bed	129_CLIPdb_PARalyzer_C17orf85.bed	HEK293
130	CLIPdb_Piranha	C17orf85	22681889	Baltz, A. G. et al.	PAR-CLIP	1048	1049	C17orf85_GSE38201-GSM936507.bed	130_CLIPdb_Piranha_C17orf85.bed	HEK293
131	CLIPdb_PARalyzer	CAPRIN1	22681889	Baltz, A. G. et al.	PAR-CLIP	89568	89571	CAPRIN1_GSE38201-GSM936509.bed	131_CLIPdb_PARalyzer_CAPRIN1.bed	HEK293
132	CLIPdb_Piranha	CAPRIN1	22681889	Baltz, A. G. et al.	PAR-CLIP	4487	4488	CAPRIN1_GSE38201-GSM936509.bed	132_CLIPdb_Piranha_CAPRIN1.bed	HEK293

133	CLIPdb_PARalyzer	CPSF1	22813749	Martin, G., Gruber, A. R., Keller, W. & Zavolan, M.	PAR-CLIP	29842	29855	CPSF1_GSE37401-GSM917672.bed	133_CLIPdb_PARalyzer_CPSF1.bed	HEK293
134	CLIPdb_PARalyzer	CPSF1	22813749	Martin, G., Gruber, A. R., Keller, W. & Zavolan, M.	PAR-CLIP	41771	41780	CPSF1_GSE37401-GSM917673.bed	134_CLIPdb_PARalyzer_CPSF1.bed	HEK293
135	CLIPdb_Piranha	CPSF1	22813749	Martin, G., Gruber, A. R., Keller, W. & Zavolan, M.	PAR-CLIP	5885	5892	CPSF1_GSE37401-GSM917672.bed	135_CLIPdb_Piranha_CPSF1.bed	HEK293
136	CLIPdb_Piranha	CPSF1	22813749	Martin, G., Gruber, A. R., Keller, W. & Zavolan, M.	PAR-CLIP	2261	2264	CPSF1_GSE37401-GSM917673.bed	136_CLIPdb_Piranha_CPSF1.bed	HEK293
137	CLIPdb_PARalyzer	CPSF2	22813749	Martin, G., Gruber, A. R., Keller, W. & Zavolan, M.	PAR-CLIP	13540	13542	CPSF2_GSE37401-GSM917670.bed	137_CLIPdb_PARalyzer_CPSF2.bed	HEK293
138	CLIPdb_PARalyzer	CPSF2	22813749	Martin, G., Gruber, A. R., Keller, W. & Zavolan, M.	PAR-CLIP	1326	1328	CPSF2_GSE37401-GSM917671.bed	138_CLIPdb_PARalyzer_CPSF2.bed	HEK293
139	CLIPdb_Piranha	CPSF2	22813749	Martin, G., Gruber, A. R., Keller, W. & Zavolan, M.	PAR-CLIP	682	682	CPSF2_GSE37401-GSM917670.bed	139_CLIPdb_Piranha_CPSF2.bed	HEK293
140	CLIPdb_Piranha	CPSF2	22813749	Martin, G., Gruber, A. R., Keller, W. & Zavolan, M.	PAR-CLIP	915	916	CPSF2_GSE37401-GSM917671.bed	140_CLIPdb_Piranha_CPSF2.bed	HEK293
141	CLIPdb_PARalyzer	CPSF3	22813749	Martin, G., Gruber, A. R., Keller, W. & Zavolan, M.	PAR-CLIP	36319	36325	CPSF3_GSE37401-GSM917668.bed	141_CLIPdb_PARalyzer_CPSF3.bed	HEK293
142	CLIPdb_PARalyzer	CPSF3	22813749	Martin, G., Gruber, A. R., Keller, W. & Zavolan, M.	PAR-CLIP	55936	55944	CPSF3_GSE37401-GSM917669.bed	142_CLIPdb_PARalyzer_CPSF3.bed	HEK293
143	CLIPdb_Piranha	CPSF3	22813749	Martin, G., Gruber, A. R., Keller, W. & Zavolan, M.	PAR-CLIP	914	915	CPSF3_GSE37401-GSM917668.bed	143_CLIPdb_Piranha_CPSF3.bed	HEK293
144	CLIPdb_Piranha	CPSF3	22813749	Martin, G., Gruber, A. R., Keller, W. & Zavolan, M.	PAR-CLIP	2058	2059	CPSF3_GSE37401-GSM917669.bed	144_CLIPdb_Piranha_CPSF3.bed	HEK293
145	CLIPdb_PARalyzer	CPSF4	22813749	Martin, G., Gruber, A. R., Keller, W. & Zavolan, M.	PAR-CLIP	16791	16793	CPSF4_GSE37401-GSM917666.bed	145_CLIPdb_PARalyzer_CPSF4.bed	HEK293
146	CLIPdb_PARalyzer	CPSF4	22813749	Martin, G., Gruber, A. R., Keller, W. & Zavolan, M.	PAR-CLIP	43245	43248	CPSF4_GSE37401-GSM917667.bed	146_CLIPdb_PARalyzer_CPSF4.bed	HEK293
147	CLIPdb_Piranha	CPSF4	22813749	Martin, G., Gruber, A. R., Keller, W. & Zavolan, M.	PAR-CLIP	1234	1237	CPSF4_GSE37401-GSM917666.bed	147_CLIPdb_Piranha_CPSF4.bed	HEK293
148	CLIPdb_Piranha	CPSF4	22813749	Martin, G., Gruber, A. R., Keller, W. & Zavolan, M.	PAR-CLIP	1349	1350	CPSF4_GSE37401-GSM917667.bed	148_CLIPdb_Piranha_CPSF4.bed	HEK293
149	CLIPdb_PARalyzer	CPSF6	22813749	Martin, G., Gruber, A. R., Keller, W. & Zavolan, M.	PAR-CLIP	103781	103793	CPSF6_GSE37401-GSM917664.bed	149_CLIPdb_PARalyzer_CPSF6.bed	HEK293
150	CLIPdb_PARalyzer	CPSF6	22813749	Martin, G., Gruber, A. R., Keller, W. & Zavolan, M.	PAR-CLIP	155733	155754	CPSF6_GSE37401-GSM917665.bed	150_CLIPdb_PARalyzer_CPSF6.bed	HEK293
151	CLIPdb_Piranha	CPSF6	22813749	Martin, G., Gruber, A. R., Keller, W. & Zavolan, M.	PAR-CLIP	23678	23685	CPSF6_GSE37401-GSM917664.bed	151_CLIPdb_Piranha_CPSF6.bed	HEK293
152	CLIPdb_Piranha	CPSF6	22813749	Martin, G., Gruber, A. R., Keller, W. & Zavolan, M.	PAR-CLIP	32332	32343	CPSF6_GSE37401-GSM917665.bed	152_CLIPdb_Piranha_CPSF6.bed	HEK293
153	CLIPdb_PARalyzer	CPSF7	22813749	Martin, G., Gruber, A. R., Keller, W. & Zavolan, M.	PAR-CLIP	300558	300577	CPSF7_GSE37401-GSM917663.bed	153_CLIPdb_PARalyzer_CPSF7.bed	HEK293
154	CLIPdb_Piranha	CPSF7	22813749	Martin, G., Gruber, A. R., Keller, W. & Zavolan, M.	PAR-CLIP	58842	58849	CPSF7_GSE37401-GSM917663.bed	154_CLIPdb_Piranha_CPSF7.bed	HEK293
155	CLIPdb_CIMS	CSTF2	23112178	Yao, C. et al.	iCLIP	210009	210045	CSTF2_GSE40859-GSM1003587.bed	155_CLIPdb_CIMS_CSTF2.bed	HeLa
156	CLIPdb_CIMS	CSTF2	23112178	Yao, C. et al.	iCLIP	305936	305964	CSTF2_GSE40859-GSM1003588.bed	156_CLIPdb_CIMS_CSTF2.bed	HeLa
157	CLIPdb_CIMS	CSTF2	23112178	Yao, C. et al.	iCLIP	339834	339872	CSTF2_GSE40859-GSM1003589.bed	157_CLIPdb_CIMS_CSTF2.bed	HeLa

158	CLIPdb_CITS	CSTF2	23112178	Yao, C. et al.	iCLIP	210009	210045	CSTF2_GSE40859-GSM1003587.bed	158_CLIPdb_CITS_CSTF2.bed	HeLa
159	CLIPdb_CITS	CSTF2	23112178	Yao, C. et al.	iCLIP	305936	305964	CSTF2_GSE40859-GSM1003588.bed	159_CLIPdb_CITS_CSTF2.bed	HeLa
160	CLIPdb_CITS	CSTF2	23112178	Yao, C. et al.	iCLIP	339834	339872	CSTF2_GSE40859-GSM1003589.bed	160_CLIPdb_CITS_CSTF2.bed	HeLa
161	CLIPdb_PARalyzer	CSTF2	22813749	Martin, G., Gruber, A. R., Keller, W. & Zavolan, M.	PAR-CLIP	233683	233718	CSTF2_GSE37401-GSM917676.bed	161_CLIPdb_PARalyzer_CSTF2.bed	HEK293
162	CLIPdb_Piranha	CSTF2	22813749	Martin, G., Gruber, A. R., Keller, W. & Zavolan, M.	PAR-CLIP	21083	21087	CSTF2_GSE37401-GSM917676.bed	162_CLIPdb_Piranha_CSTF2.bed	HEK293
163	CLIPdb_Piranha	CSTF2	23112178	Yao, C. et al.	iCLIP	49436	49449	CSTF2_GSE40859-GSM1003587.bed	163_CLIPdb_Piranha_CSTF2.bed	HeLa
164	CLIPdb_Piranha	CSTF2	23112178	Yao, C. et al.	iCLIP	49595	49608	CSTF2_GSE40859-GSM1003588.bed	164_CLIPdb_Piranha_CSTF2.bed	HeLa
165	CLIPdb_Piranha	CSTF2	23112178	Yao, C. et al.	iCLIP	52823	52837	CSTF2_GSE40859-GSM1003589.bed	165_CLIPdb_Piranha_CSTF2.bed	HeLa
166	CLIPdb_PARalyzer	CSTF2T	22813749	Martin, G., Gruber, A. R., Keller, W. & Zavolan, M.	PAR-CLIP	489717	489775	CSTF2T_GSE37401-GSM917677.bed	166_CLIPdb_PARalyzer_CSTF2T.bed	HEK293
167	CLIPdb_PARalyzer	CSTF2T	22813749	Martin, G., Gruber, A. R., Keller, W. & Zavolan, M.	PAR-CLIP	16356	16359	CSTF2T_GSE37401-GSM917678.bed	167_CLIPdb_PARalyzer_CSTF2T.bed	HEK293
168	CLIPdb_Piranha	CSTF2T	22813749	Martin, G., Gruber, A. R., Keller, W. & Zavolan, M.	PAR-CLIP	38482	38493	CSTF2T_GSE37401-GSM917677.bed	168_CLIPdb_Piranha_CSTF2T.bed	HEK293
169	CLIPdb_Piranha	CSTF2T	22813749	Martin, G., Gruber, A. R., Keller, W. & Zavolan, M.	PAR-CLIP	15839	15840	CSTF2T_GSE37401-GSM917678.bed	169_CLIPdb_Piranha_CSTF2T.bed	HEK293
170	CLIPdb_CIMS	DGCR8	22796965	Macias, S. et al.	HITS-CLIP	132	132	DGCR8_GSE39086-GSM955510.bed	170_CLIPdb_CIMS_DGCR8.bed	HEK293T
171	CLIPdb_CIMS	DGCR8	22796965	Macias, S. et al.	HITS-CLIP	87	87	DGCR8_GSE39086-GSM955511.bed	171_CLIPdb_CIMS_DGCR8.bed	HEK293T
172	CLIPdb_CIMS	DGCR8	22796965	Macias, S. et al.	HITS-CLIP	842	845	DGCR8_GSE39086-GSM955512.bed	172_CLIPdb_CIMS_DGCR8.bed	HEK293T
173	CLIPdb_CIMS	DGCR8	22796965	Macias, S. et al.	HITS-CLIP	728	729	DGCR8_GSE39086-GSM955513.bed	173_CLIPdb_CIMS_DGCR8.bed	HEK293T
174	CLIPdb_Piranha	DGCR8	22796965	Macias, S. et al.	HITS-CLIP	383	383	DGCR8_GSE39086-GSM955510.bed	174_CLIPdb_Piranha_DGCR8.bed	HEK293T
175	CLIPdb_Piranha	DGCR8	22796965	Macias, S. et al.	HITS-CLIP	172	172	DGCR8_GSE39086-GSM955511.bed	175_CLIPdb_Piranha_DGCR8.bed	HEK293T
176	CLIPdb_Piranha	DGCR8	22796965	Macias, S. et al.	HITS-CLIP	3788	3792	DGCR8_GSE39086-GSM955512.bed	176_CLIPdb_Piranha_DGCR8.bed	HEK293T
177	CLIPdb_Piranha	DGCR8	22796965	Macias, S. et al.	HITS-CLIP	4885	4890	DGCR8_GSE39086-GSM955513.bed	177_CLIPdb_Piranha_DGCR8.bed	HEK293T
178	CLIPdb_CIMS	EIF4A3	23085716	Sauliere, J. et al.	HITS-CLIP	504	505	EIF4A3_GSE40778-GSM1001330.bed	178_CLIPdb_CIMS_EIF4A3.bed	HeLa
179	CLIPdb_CIMS	EIF4A3	23085716	Sauliere, J. et al.	HITS-CLIP	452	453	EIF4A3_GSE40778-GSM1001331.bed	179_CLIPdb_CIMS_EIF4A3.bed	HeLa
180	CLIPdb_Piranha	EIF4A3	23085716	Sauliere, J. et al.	HITS-CLIP	4419	4420	EIF4A3_GSE40778-GSM1001330.bed	180_CLIPdb_Piranha_EIF4A3.bed	HeLa
181	CLIPdb_Piranha	EIF4A3	23085716	Sauliere, J. et al.	HITS-CLIP	19015	19019	EIF4A3_GSE40778-GSM1001331.bed	181_CLIPdb_Piranha_EIF4A3.bed	HeLa
182	CLIPdb_CIMS	ELAVL1	21572407	Kishore, S. et al.	HITS-CLIP	2172	2173	ELAVL1_GSE28865-GSM714635.bed	182_CLIPdb_CIMS_ELAVL1.bed	HEK293
183	CLIPdb_CIMS	ELAVL1	21572407	Kishore, S. et al.	HITS-CLIP	1381	1383	ELAVL1_GSE28865-GSM714636.bed	183_CLIPdb_CIMS_ELAVL1.bed	HEK293
184	CLIPdb_PARalyzer	ELAVL1	21572407	Kishore, S. et al.	PAR-CLIP	92812	92932	ELAVL1_GSE28865-GSM714637.bed	184_CLIPdb_PARalyzer_ELAVL1.bed	HEK293
185	CLIPdb_PARalyzer	ELAVL1	21572407	Kishore, S. et al.	PAR-CLIP	79692	79791	ELAVL1_GSE28865-GSM714638.bed	185_CLIPdb_PARalyzer_ELAVL1.bed	HEK293
186	CLIPdb_PARalyzer	ELAVL1	21572407	Kishore, S. et al.	PAR-CLIP	320634	320689	ELAVL1_GSE28865-GSM714639.bed	186_CLIPdb_PARalyzer_ELAVL1.bed	HEK293
187	CLIPdb_PARalyzer	ELAVL1	21572407	Kishore, S. et al.	PAR-CLIP	268666	268767	ELAVL1_GSE28865-GSM714640.bed	187_CLIPdb_PARalyzer_ELAVL1.bed	HEK293
188	CLIPdb_PARalyzer	ELAVL1	21572407	Kishore, S. et al.	PAR-CLIP	182336	182434	ELAVL1_GSE28865-GSM714641.bed	188_CLIPdb_PARalyzer_ELAVL1.bed	HEK293
189	CLIPdb_PARalyzer	ELAVL1	21723170	Mukherjee, N. et al.	PAR-CLIP	97809	97832	ELAVL1_GSE29780-GSM738185.bed	189_CLIPdb_PARalyzer_ELAVL1.bed	HEK293
190	CLIPdb_PARalyzer	ELAVL1	24393468	Friedersdorf, M. B. & Keene, J. D.	PAR-CLIP	31526	31530	ELAVL1_GSE50989-GSM1234283.bed	190_CLIPdb_PARalyzer_ELAVL1.bed	HEK293
191	CLIPdb_PARalyzer	ELAVL1	24393468	Friedersdorf, M. B. & Keene, J. D.	PAR-CLIP	1128905	1128979	ELAVL1_GSE50989-GSM1234284.bed	191_CLIPdb_PARalyzer_ELAVL1.bed	HEK293
192	CLIPdb_PARalyzer	ELAVL1	21723171	Lebedeva, S. et al.	PAR-CLIP	6280	6280	ELAVL1_GSE29943-GSM741173.bed	192_CLIPdb_PARalyzer_ELAVL1.bed	HeLa
193	CLIPdb_PARalyzer	ELAVL1	21723171	Lebedeva, S. et al.	PAR-CLIP	11695	11695	ELAVL1_GSE29943-GSM741174.bed	193_CLIPdb_PARalyzer_ELAVL1.bed	HeLa
194	CLIPdb_PARalyzer	ELAVL1	21723171	Lebedeva, S. et al.	PAR-CLIP	4033	4034	ELAVL1_GSE29943-GSM741175.bed	194_CLIPdb_PARalyzer_ELAVL1.bed	HeLa
195	CLIPdb_Piranha	ELAVL1	21572407	Kishore, S. et al.	HITS-CLIP	2918	2927	ELAVL1_GSE28865-GSM714635.bed	195_CLIPdb_Piranha_ELAVL1.bed	HEK293
196	CLIPdb_Piranha	ELAVL1	21572407	Kishore, S. et al.	HITS-CLIP	2948	2954	ELAVL1_GSE28865-GSM714636.bed	196_CLIPdb_Piranha_ELAVL1.bed	HEK293
197	CLIPdb_Piranha	ELAVL1	21572407	Kishore, S. et al.	PAR-CLIP	6275	6281	ELAVL1_GSE28865-GSM714637.bed	197_CLIPdb_Piranha_ELAVL1.bed	HEK293
198	CLIPdb_Piranha	ELAVL1	21572407	Kishore, S. et al.	PAR-CLIP	6089	6093	ELAVL1_GSE28865-GSM714638.bed	198_CLIPdb_Piranha_ELAVL1.bed	HEK293

199	CLIPdb_Piranha	ELAVL1	21572407	Kishore, S. et al.	PAR-CLIP	48022	48036	ELAVL1_GSE28865-GSM714639.bed	199_CLIPdb_Piranha_ELAVL1.bed	HEK293
200	CLIPdb_Piranha	ELAVL1	21572407	Kishore, S. et al.	PAR-CLIP	44849	44872	ELAVL1_GSE28865-GSM714640.bed	200_CLIPdb_Piranha_ELAVL1.bed	HEK293
201	CLIPdb_Piranha	ELAVL1	21572407	Kishore, S. et al.	PAR-CLIP	33809	33824	ELAVL1_GSE28865-GSM714641.bed	201_CLIPdb_Piranha_ELAVL1.bed	HEK293
202	CLIPdb_Piranha	ELAVL1	21723170	Mukherjee, N. et al.	PAR-CLIP	13205	13207	ELAVL1_GSE29780-GSM738185.bed	202_CLIPdb_Piranha_ELAVL1.bed	HEK293
203	CLIPdb_Piranha	ELAVL1	24393468	Friedersdorf, M. B. & Keene, J. D.	PAR-CLIP	33969	33976	ELAVL1_GSE50989-GSM1234283.bed	203_CLIPdb_Piranha_ELAVL1.bed	HEK293
204	CLIPdb_Piranha	ELAVL1	24393468	Friedersdorf, M. B. & Keene, J. D.	PAR-CLIP	49132	49139	ELAVL1_GSE50989-GSM1234284.bed	204_CLIPdb_Piranha_ELAVL1.bed	HEK293
205	CLIPdb_Piranha	ELAVL1	21723171	Lebedeva, S. et al.	PAR-CLIP	5448	5450	ELAVL1_GSE29943-GSM741173.bed	205_CLIPdb_Piranha_ELAVL1.bed	HeLa
206	CLIPdb_Piranha	ELAVL1	21723171	Lebedeva, S. et al.	PAR-CLIP	3043	3044	ELAVL1_GSE29943-GSM741174.bed	206_CLIPdb_Piranha_ELAVL1.bed	HeLa
207	CLIPdb_Piranha	ELAVL1	21723171	Lebedeva, S. et al.	PAR-CLIP	5884	5884	ELAVL1_GSE29943-GSM741175.bed	207_CLIPdb_Piranha_ELAVL1.bed	HeLa
208	CLIPdb_PARalyzer	EWSR1	22081015	Hoell, J. I. et al.	PAR-CLIP	45789	45792	EWSR1_SRX029343.bed	210_CLIPdb_PARalyzer_EWSR1.bed	HEK293
209	CLIPdb_PARalyzer	EWSR1	22081015	Hoell, J. I. et al.	PAR-CLIP	37572	37572	EWSR1_SRX029344.bed	211_CLIPdb_PARalyzer_EWSR1.bed	HEK293
210	CLIPdb_Piranha	EWSR1	22081015	Hoell, J. I. et al.	PAR-CLIP	5942	5943	EWSR1_SRX029343.bed	212_CLIPdb_Piranha_EWSR1.bed	HEK293
211	CLIPdb_Piranha	EWSR1	22081015	Hoell, J. I. et al.	PAR-CLIP	2682	2682	EWSR1_SRX029344.bed	213_CLIPdb_Piranha_EWSR1.bed	HEK293
212	CLIPdb_CIMS	EZH2	24141703	Guil, S. et al.	iCLIP	10	10	EZH2_GSE36070-GSM880698.bed	216_CLIPdb_CIMS_EZH2.bed	HCT116
213	CLIPdb_CITS	EZH2	24141703	Guil, S. et al.	iCLIP	10	10	EZH2_GSE36070-GSM880698.bed	217_CLIPdb_CITS_EZH2.bed	HCT116
214	CLIPdb_Piranha	EZH2	24141703	Guil, S. et al.	iCLIP	269	269	EZH2_GSE36070-GSM880698.bed	218_CLIPdb_Piranha_EZH2.bed	HCT116
215	CLIPdb_PARalyzer	FBL	23706177	Kishore, S. et al.	PAR-CLIP	4219	4222	FBL_GSE43666-GSM1067864.bed	219_CLIPdb_PARalyzer_FBL.bed	HEK293
216	CLIPdb_PARalyzer	FBL	23706177	Kishore, S. et al.	PAR-CLIP	3371	3373	FBL_GSE43666-GSM1067865.bed	220_CLIPdb_PARalyzer_FBL.bed	HEK293
217	CLIPdb_Piranha	FBL	23706177	Kishore, S. et al.	PAR-CLIP	1434	1435	FBL_GSE43666-GSM1067864.bed	221_CLIPdb_Piranha_FBL.bed	HEK293
218	CLIPdb_Piranha	FBL	23706177	Kishore, S. et al.	PAR-CLIP	1304	1304	FBL_GSE43666-GSM1067865.bed	222_CLIPdb_Piranha_FBL.bed	HEK293
219	CLIPdb_PARalyzer	FIP1L1	22813749	Martin, G., Gruber, A. R., Keller, W. & Zavolan, M.	PAR-CLIP	231633	231658	FIP1L1_GSE37401-GSM917674.bed	223_CLIPdb_PARalyzer_FIP1L1.bed	HEK293
220	CLIPdb_PARalyzer	FIP1L1	22813749	Martin, G., Gruber, A. R., Keller, W. & Zavolan, M.	PAR-CLIP	186766	186787	FIP1L1_GSE37401-GSM917675.bed	224_CLIPdb_PARalyzer_FIP1L1.bed	HEK293
221	CLIPdb_Piranha	FIP1L1	22813749	Martin, G., Gruber, A. R., Keller, W. & Zavolan, M.	PAR-CLIP	4209	4212	FIP1L1_GSE37401-GSM917674.bed	225_CLIPdb_Piranha_FIP1L1.bed	HEK293
222	CLIPdb_Piranha	FIP1L1	22813749	Martin, G., Gruber, A. R., Keller, W. & Zavolan, M.	PAR-CLIP	24755	24769	FIP1L1_GSE37401-GSM917675.bed	226_CLIPdb_Piranha_FIP1L1.bed	HEK293
223	CLIPdb_PARalyzer	FMR1	23235829	Ascano, M., Jr. et al.	PAR-CLIP	102750	102754	FMR1_GSE39682-GSM977615.bed	227_CLIPdb_PARalyzer_FMR1.bed	HEK293
224	CLIPdb_PARalyzer	FMR1	23235829	Ascano, M., Jr. et al.	PAR-CLIP	60480	60486	FMR1_GSE39682-GSM977616.bed	228_CLIPdb_PARalyzer_FMR1.bed	HEK293
225	CLIPdb_Piranha	FMR1	23235829	Ascano, M., Jr. et al.	PAR-CLIP	13680	13682	FMR1_GSE39682-GSM977615.bed	229_CLIPdb_Piranha_FMR1.bed	HEK293
226	CLIPdb_Piranha	FMR1	23235829	Ascano, M., Jr. et al.	PAR-CLIP	9087	9090	FMR1_GSE39682-GSM977616.bed	230_CLIPdb_Piranha_FMR1.bed	HEK293
227	CLIPdb_CIMS	FUS	23023293	Lagier-Tourenne, C. et al.	HITS-CLIP	5329	5337	FUS_GSE40653-GSM998875.bed	231_CLIPdb_CIMS_FUS.bed	Brain
228	CLIPdb_CIMS	FUS	23389473	Nakaya, T., Alexiou, P., Maragkakis, M., Chang, A. & Mourelatos, Z.	HITS-CLIP	116	116	FUS_GSE43308-GSM1060381.bed	232_CLIPdb_CIMS_FUS.bed	Brain
229	CLIPdb_CIMS	FUS	23389473	Nakaya, T., Alexiou, P., Maragkakis, M., Chang, A. & Mourelatos, Z.	HITS-CLIP	96	96	FUS_GSE43308-GSM1060382.bed	233_CLIPdb_CIMS_FUS.bed	Brain
230	CLIPdb_CIMS	FUS	23389473	Nakaya, T., Alexiou, P., Maragkakis, M., Chang, A. & Mourelatos, Z.	HITS-CLIP	108	108	FUS_GSE43308-GSM1060383.bed	234_CLIPdb_CIMS_FUS.bed	Brain
231	CLIPdb_PARalyzer	FUS	22081015	Hoell, J. I. et al.	PAR-CLIP	67946	67950	FUS_SRX029328.bed	235_CLIPdb_PARalyzer_FUS.bed	HEK293
232	CLIPdb_PARalyzer	FUS	22081015	Hoell, J. I. et al.	PAR-CLIP	31455	31455	FUS_SRX029329.bed	236_CLIPdb_PARalyzer_FUS.bed	HEK293
233	CLIPdb_PARalyzer	FUS	24954906	Kawahara, Y. et al.	PAR-CLIP	46397	46401	FUS_DRA001158-DRS012384.bed	237_CLIPdb_PARalyzer_FUS.bed	HEK293T
234	CLIPdb_Piranha	FUS	23023293	Lagier-Tourenne, C. et al.	HITS-CLIP	109490	109496	FUS_GSE40653-GSM998875.bed	238_CLIPdb_Piranha_FUS.bed	Brain
235	CLIPdb_Piranha	FUS	23389473	Nakaya, T., Alexiou, P., Maragkakis, M., Chang, A. & Mourelatos, Z.	HITS-CLIP	2314	2314	FUS_GSE43308-GSM1060381.bed	239_CLIPdb_Piranha_FUS.bed	Brain
236	CLIPdb_Piranha	FUS	23389473	Nakaya, T., Alexiou, P., Maragkakis, M., Chang, A. & Mourelatos, Z.	HITS-CLIP	1898	1898	FUS_GSE43308-GSM1060382.bed	240_CLIPdb_Piranha_FUS.bed	Brain

237	CLIPdb_Piranha	FUS	23389473	Nakaya, T., Alexiou, P., Maragkakis, M., Chang, A. & Mourelatos, Z.	HITS-CLIP	2418	2418	FUS_GSE43308-GSM1060383.bed	241_CLIPdb_Piranha_FUS.bed	Brain
238	CLIPdb_Piranha	FUS	22081015	Hoell, J. I. et al.	PAR-CLIP	2248	2248	FUS_SRX029328.bed	242_CLIPdb_Piranha_FUS.bed	HEK293
239	CLIPdb_Piranha	FUS	22081015	Hoell, J. I. et al.	PAR-CLIP	12898	12899	FUS_SRX029329.bed	243_CLIPdb_Piranha_FUS.bed	HEK293
240	CLIPdb_Piranha	FUS	24954906	Kawahara, Y. et al.	PAR-CLIP	712	712	FUS_DRA001158-DRS012384.bed	244_CLIPdb_Piranha_FUS.bed	HEK293T
241	CLIPdb_PARalyzer	FXR1	23235829	Ascano, M., Jr. et al.	PAR-CLIP	4449	4449	FXR1_GSE39682-GSM977619.bed	245_CLIPdb_PARalyzer_FXR1.bed	HEK293
242	CLIPdb_Piranha	FXR1	23235829	Ascano, M., Jr. et al.	PAR-CLIP	2262	2262	FXR1_GSE39682-GSM977619.bed	246_CLIPdb_Piranha_FXR1.bed	HEK293
243	CLIPdb_PARalyzer	FXR2	23235829	Ascano, M., Jr. et al.	PAR-CLIP	23734	23734	FXR2_GSE39682-GSM977620.bed	247_CLIPdb_PARalyzer_FXR2.bed	HEK293
244	CLIPdb_Piranha	FXR2	23235829	Ascano, M., Jr. et al.	PAR-CLIP	6905	6905	FXR2_GSE39682-GSM977620.bed	248_CLIPdb_Piranha_FXR2.bed	HEK293
245	CLIPdb_CIMS	HNRNPA1	22574288	Huelga, S. C. et al.	HITS-CLIP	229	229	HNRNPA1_GSE34996-GSM859978-GSM859979-GSM859980-GSM859981.bed	249_CLIPdb_CIMS_HNRNPA1.bed	HEK293T
246	CLIPdb_Piranha	HNRNPA1	22574288	Huelga, S. C. et al.	HITS-CLIP	15577	15580	HNRNPA1_GSE34996-GSM859978-GSM859979-GSM859980-GSM859981.bed	250_CLIPdb_Piranha_HNRNPA1.bed	HEK293T
247	CLIPdb_CIMS	HNRNPA2B1	22574288	Huelga, S. C. et al.	HITS-CLIP	142	145	HNRNPA2B1_GSE34996-GSM859982.bed	251_CLIPdb_CIMS_HNRNPA2B1.bed	HEK293T
248	CLIPdb_Piranha	HNRNPA2B1	22574288	Huelga, S. C. et al.	HITS-CLIP	1783	1784	HNRNPA2B1_GSE34996-GSM859982.bed	252_CLIPdb_Piranha_HNRNPA2B1.bed	HEK293T
249	CLIPdb_CIMS	HNRNPC	23374342	Zarnack, K. et al.	iCLIP	358276	358379	HNRNPC_E-MTAB-1371-ERR196174-ERR196175-ERR196179-ERR196187.bed	253_CLIPdb_CIMS_HNRNPC.bed	HeLa
250	CLIPdb_CIMS	HNRNPC	23374342	Zarnack, K. et al.	iCLIP	466931	467028	HNRNPC_E-MTAB-1371-ERR196176-ERR196177-ERR196178-ERR196181-ERR196182-ERR196185-ERR196186-ERR196188-ERR196189.bed	254_CLIPdb_CIMS_HNRNPC.bed	HeLa
251	CLIPdb_CIMS	HNRNPC	20601959	Konig, J. et al.	iCLIP	6507	6510	HNRNPC_E-MTAB-341-ERR018282.bed	255_CLIPdb_CIMS_HNRNPC.bed	HeLa
252	CLIPdb_CIMS	HNRNPC	20601959	Konig, J. et al.	iCLIP	4323	4328	HNRNPC_E-MTAB-341-ERR018283.bed	256_CLIPdb_CIMS_HNRNPC.bed	HeLa
253	CLIPdb_CIMS	HNRNPC	20601959	Konig, J. et al.	iCLIP	1732	1736	HNRNPC_E-MTAB-341-ERR018284.bed	257_CLIPdb_CIMS_HNRNPC.bed	HeLa
254	CLIPdb_CITS	HNRNPC	23374342	Zarnack, K. et al.	iCLIP	358276	358379	HNRNPC_E-MTAB-1371-ERR196174-ERR196175-ERR196179-ERR196187.bed	258_CLIPdb_CITS_HNRNPC.bed	HeLa
255	CLIPdb_CITS	HNRNPC	23374342	Zarnack, K. et al.	iCLIP	466931	467028	HNRNPC_E-MTAB-1371-ERR196176-ERR196177-ERR196178-ERR196181-ERR196182-ERR196185-ERR196186-ERR196188-ERR196189.bed	259_CLIPdb_CITS_HNRNPC.bed	HeLa
256	CLIPdb_CITS	HNRNPC	20601959	Konig, J. et al.	iCLIP	6507	6510	HNRNPC_E-MTAB-341-ERR018282.bed	260_CLIPdb_CITS_HNRNPC.bed	HeLa
257	CLIPdb_CITS	HNRNPC	20601959	Konig, J. et al.	iCLIP	4323	4328	HNRNPC_E-MTAB-341-ERR018283.bed	261_CLIPdb_CITS_HNRNPC.bed	HeLa
258	CLIPdb_CITS	HNRNPC	20601959	Konig, J. et al.	iCLIP	1732	1736	HNRNPC_E-MTAB-341-ERR018284.bed	262_CLIPdb_CITS_HNRNPC.bed	HeLa
259	CLIPdb_Piranha	HNRNPC	23374342	Zarnack, K. et al.	iCLIP	31016	31020	HNRNPC_E-MTAB-1371-ERR196174-ERR196175-ERR196179-ERR196187.bed	263_CLIPdb_Piranha_HNRNPC.bed	HeLa
260	CLIPdb_Piranha	HNRNPC	23374342	Zarnack, K. et al.	iCLIP	60728	60736	HNRNPC_E-MTAB-1371-ERR196176-ERR196177-ERR196178-ERR196181-ERR196182-ERR196185-ERR196186-ERR196188-ERR196189.bed	264_CLIPdb_Piranha_HNRNPC.bed	HeLa
261	CLIPdb_Piranha	HNRNPC	20601959	Konig, J. et al.	iCLIP	3358	3358	HNRNPC_E-MTAB-341-ERR018282.bed	265_CLIPdb_Piranha_HNRNPC.bed	HeLa
262	CLIPdb_Piranha	HNRNPC	20601959	Konig, J. et al.	iCLIP	2704	2704	HNRNPC_E-MTAB-341-ERR018283.bed	266_CLIPdb_Piranha_HNRNPC.bed	HeLa
263	CLIPdb_Piranha	HNRNPC	20601959	Konig, J. et al.	iCLIP	1648	1649	HNRNPC_E-MTAB-341-ERR018284.bed	267_CLIPdb_Piranha_HNRNPC.bed	HeLa
264	CLIPdb_PARalyzer	HNRNPD	25366541	Hafner M et al.	PAR-CLIP	32415	32416	HNRNPD_GSE52971-GSM1279666.bed	268_CLIPdb_PARalyzer_HNRNPD.bed	HEK293
265	CLIPdb_PARalyzer	HNRNPD	25366541	Hafner M et al.	PAR-CLIP	39621	39627	HNRNPD_GSE52971-GSM1279667.bed	269_CLIPdb_PARalyzer_HNRNPD.bed	HEK293
266	CLIPdb_PARalyzer	HNRNPD	25366541	Hafner M et al.	PAR-CLIP	7699	7701	HNRNPD_GSE52971-GSM1279665.bed	270_CLIPdb_PARalyzer_HNRNPD.bed	HEK293
267	CLIPdb_PARalyzer	HNRNPD	25366541	Hafner M et al.	PAR-CLIP	2619	2621	HNRNPD_GSE52971-GSM1279664.bed	271_CLIPdb_PARalyzer_HNRNPD.bed	HEK293
268	CLIPdb_Piranha	HNRNPD	25366541	Hafner M et al.	PAR-CLIP	2749	2752	HNRNPD_GSE52971-GSM1279666.bed	272_CLIPdb_Piranha_HNRNPD.bed	HEK293

269	CLIPdb_Piranha	HNRNPD	25366541	Hafner M et al.	PAR-CLIP	10922	10924	HNRNPD_GSE52971-GSM1279667.bed	273_CLIPdb_Piranha_HNRNPD.bed	HEK293
270	CLIPdb_Piranha	HNRNPD	25366541	Hafner M et al.	PAR-CLIP	1230	1232	HNRNPD_GSE52971-GSM1279665.bed	274_CLIPdb_Piranha_HNRNPD.bed	HEK293
271	CLIPdb_Piranha	HNRNPD	25366541	Hafner M et al.	PAR-CLIP	599	599	HNRNPD_GSE52971-GSM1279664.bed	275_CLIPdb_Piranha_HNRNPD.bed	HEK293
272	CLIPdb_CIMS	HNRNPF	22574288	Huelga, S. C. et al.	HITS-CLIP	320	325	HNRNPF_GSE34996-GSM859983-GSM859984-GSM859985-GSM859986.bed	276_CLIPdb_CIMS_HNRNPF.bed	HEK293T
273	CLIPdb_Piranha	HNRNPF	22574288	Huelga, S. C. et al.	HITS-CLIP	3892	3895	HNRNPF_GSE34996-GSM859983-GSM859984-GSM859985-GSM859986.bed	277_CLIPdb_Piranha_HNRNPF.bed	HEK293T
274	CLIPdb_CIMS	HNRNPH	21057496	Katz, Y., Wang, E. T., Airoldi, E. M. & Burge, C. B.	HITS-CLIP	587	588	HNRNPH_GSE23694-GSM581051.bed	278_CLIPdb_CIMS_HNRNPH.bed	HEK293T
275	CLIPdb_CIMS	HNRNPH	21057496	Katz, Y., Wang, E. T., Airoldi, E. M. & Burge, C. B.	HITS-CLIP	793	795	HNRNPH_GSE23694-GSM581052.bed	279_CLIPdb_CIMS_HNRNPH.bed	HEK293T
276	CLIPdb_Piranha	HNRNPH	21057496	Katz, Y., Wang, E. T., Airoldi, E. M. & Burge, C. B.	HITS-CLIP	2314	2315	HNRNPH_GSE23694-GSM581051.bed	280_CLIPdb_Piranha_HNRNPH.bed	HEK293T
277	CLIPdb_Piranha	HNRNPH	21057496	Katz, Y., Wang, E. T., Airoldi, E. M. & Burge, C. B.	HITS-CLIP	2271	2273	HNRNPH_GSE23694-GSM581052.bed	281_CLIPdb_Piranha_HNRNPH.bed	HEK293T
278	CLIPdb_CIMS	HNRNPM	22574288	Huelga, S. C. et al.	HITS-CLIP	618	621	HNRNPM_GSE34996-GSM859987-GSM859988.bed	282_CLIPdb_CIMS_HNRNPM.bed	HEK293T
279	CLIPdb_Piranha	HNRNPM	22574288	Huelga, S. C. et al.	HITS-CLIP	14951	14952	HNRNPM_GSE34996-GSM859987-GSM859988.bed	283_CLIPdb_Piranha_HNRNPM.bed	HEK293T
280	CLIPdb_CIMS	HNRNPU	22574288	Huelga, S. C. et al.	HITS-CLIP	222	223	HNRNPU_GSE34996-GSM859989-GSM859990-GSM859991-GSM859992.bed	284_CLIPdb_CIMS_HNRNPU.bed	HEK293T
281	CLIPdb_CIMS	HNRNPU	22325991	Xiao, R. et al.	HITS-CLIP	624	628	HNRNPU_GSE34491-GSM850170.bed	285_CLIPdb_CIMS_HNRNPU.bed	HeLa
282	CLIPdb_CIMS	HNRNPU	22325991	Xiao, R. et al.	HITS-CLIP	522	523	HNRNPU_GSE34491-GSM850171.bed	286_CLIPdb_CIMS_HNRNPU.bed	HeLa
283	CLIPdb_Piranha	HNRNPU	22574288	Huelga, S. C. et al.	HITS-CLIP	875	877	HNRNPU_GSE34996-GSM859989-GSM859990-GSM859991-GSM859992.bed	287_CLIPdb_Piranha_HNRNPU.bed	HEK293T
284	CLIPdb_Piranha	HNRNPU	22325991	Xiao, R. et al.	HITS-CLIP	12893	12904	HNRNPU_GSE34491-GSM850170.bed	288_CLIPdb_Piranha_HNRNPU.bed	HeLa
285	CLIPdb_Piranha	HNRNPU	22325991	Xiao, R. et al.	HITS-CLIP	1519	1526	HNRNPU_GSE34491-GSM850171.bed	289_CLIPdb_Piranha_HNRNPU.bed	HeLa
286	CLIPdb_PARalyzer	IGF2BP1	20371350	Hafner, M. et al.	PAR-CLIP	63011	63015	IGF2BP1_GSE21578-GSM545206.bed	290_CLIPdb_PARalyzer_IGF2BP1.bed	HEK293
287	CLIPdb_PARalyzer	IGF2BP1	20371350	Hafner, M. et al.	PAR-CLIP	7240	7240	IGF2BP1_GSE21578-GSM545207.bed	291_CLIPdb_PARalyzer_IGF2BP1.bed	HEK293
288	CLIPdb_Piranha	IGF2BP1	20371350	Hafner, M. et al.	PAR-CLIP	8803	8805	IGF2BP1_GSE21578-GSM545206.bed	292_CLIPdb_Piranha_IGF2BP1.bed	HEK293
289	CLIPdb_Piranha	IGF2BP1	20371350	Hafner, M. et al.	PAR-CLIP	6657	6658	IGF2BP1_GSE21578-GSM545207.bed	293_CLIPdb_Piranha_IGF2BP1.bed	HEK293
290	CLIPdb_PARalyzer	IGF2BP2	20371350	Hafner, M. et al.	PAR-CLIP	62312	62316	IGF2BP2_GSE21578-GSM545208.bed	294_CLIPdb_PARalyzer_IGF2BP2.bed	HEK293
291	CLIPdb_Piranha	IGF2BP2	20371350	Hafner, M. et al.	PAR-CLIP	9418	9419	IGF2BP2_GSE21578-GSM545208.bed	295_CLIPdb_Piranha_IGF2BP2.bed	HEK293
292	CLIPdb_PARalyzer	IGF2BP3	20371350	Hafner, M. et al.	PAR-CLIP	84085	84091	IGF2BP3_GSE21578-GSM545209.bed	296_CLIPdb_PARalyzer_IGF2BP3.bed	HEK293
293	CLIPdb_Piranha	IGF2BP3	20371350	Hafner, M. et al.	PAR-CLIP	10276	10276	IGF2BP3_GSE21578-GSM545209.bed	297_CLIPdb_Piranha_IGF2BP3.bed	HEK293
294	CLIPdb_CIMS	LIN28A	22959275	Wilbert, M. L. et al.	HITS-CLIP	2340	2342	LIN28A_GSE39873-GSM980593.bed	298_CLIPdb_CIMS_LIN28A.bed	H9
295	CLIPdb_CIMS	LIN28A	22959275	Wilbert, M. L. et al.	HITS-CLIP	1589	1589	LIN28A_GSE39873-GSM980594.bed	299_CLIPdb_CIMS_LIN28A.bed	HEK293
296	CLIPdb_PARalyzer	LIN28A	23481595	Hafner, M. et al.	PAR-CLIP	28102	28105	LIN28A_GSE44616-GSM1087848.bed	300_CLIPdb_PARalyzer_LIN28A.bed	HEK293
297	CLIPdb_Piranha	LIN28A	22959275	Wilbert, M. L. et al.	HITS-CLIP	6668	6669	LIN28A_GSE39873-GSM980593.bed	301_CLIPdb_Piranha_LIN28A.bed	H9
298	CLIPdb_Piranha	LIN28A	22959275	Wilbert, M. L. et al.	HITS-CLIP	3640	3640	LIN28A_GSE39873-GSM980594.bed	302_CLIPdb_Piranha_LIN28A.bed	HEK293
299	CLIPdb_Piranha	LIN28A	23481595	Hafner, M. et al.	PAR-CLIP	2420	2420	LIN28A_GSE44616-GSM1087848.bed	303_CLIPdb_Piranha_LIN28A.bed	HEK293
300	CLIPdb_PARalyzer	LIN28B	23481595	Hafner, M. et al.	PAR-CLIP	2667	2667	LIN28B_GSE44616-GSM1087849.bed	304_CLIPdb_PARalyzer_LIN28B.bed	HEK293
301	CLIPdb_PARalyzer	LIN28B	23481595	Hafner, M. et al.	PAR-CLIP	6901	6901	LIN28B_GSE44616-GSM1087850.bed	305_CLIPdb_PARalyzer_LIN28B.bed	HEK293
302	CLIPdb_PARalyzer	LIN28B	23481595	Hafner, M. et al.	PAR-CLIP	26326	26326	LIN28B_GSE44616-GSM1087851.bed	306_CLIPdb_PARalyzer_LIN28B.bed	HEK293
303	CLIPdb_PARalyzer	LIN28B	23770886	Graf, R. et al.	PAR-CLIP	351772	351797	LIN28B_GSE46908-GSM1140829.bed	307_CLIPdb_PARalyzer_LIN28B.bed	HEK293
304	CLIPdb_Piranha	LIN28B	23481595	Hafner, M. et al.	PAR-CLIP	987	987	LIN28B_GSE44616-GSM1087849.bed	308_CLIPdb_Piranha_LIN28B.bed	HEK293
305	CLIPdb_Piranha	LIN28B	23481595	Hafner, M. et al.	PAR-CLIP	2961	2964	LIN28B_GSE44616-GSM1087850.bed	309_CLIPdb_Piranha_LIN28B.bed	HEK293

306	CLIPdb_Piranha	LIN28B	23481595	Hafner, M. et al.	PAR-CLIP	3530	3530	LIN28B_GSE44616-GSM1087851.bed	310_CLIPdb_Piranha_LIN28B.bed	HEK293
307	CLIPdb_Piranha	LIN28B	23770886	Graf, R. et al.	PAR-CLIP	14133	14135	LIN28B_GSE46908-GSM1140829.bed	311_CLIPdb_Piranha_LIN28B.bed	HEK293
308	CLIPdb_PARalyzer	MOV10	22844102	Sievers, C., Schlumpf, T., Sawarkar, R., Comoglio, F. & Paro, R.	PAR-CLIP	177237	177246	MOV10_GSE37524-GSM921128.bed	312_CLIPdb_PARalyzer_MOV10.bed	HEK293
309	CLIPdb_Piranha	MOV10	22844102	Sievers, C., Schlumpf, T., Sawarkar, R., Comoglio, F. & Paro, R.	PAR-CLIP	13839	13839	MOV10_GSE37524-GSM921128.bed	313_CLIPdb_Piranha_MOV10.bed	HEK293
310	CLIPdb_PARalyzer	NOP56	23706177	Kishore, S. et al.	PAR-CLIP	6214	6219	NOP56_GSE43666-GSM1067863.bed	314_CLIPdb_PARalyzer_NOP56.bed	HEK293
311	CLIPdb_Piranha	NOP56	23706177	Kishore, S. et al.	PAR-CLIP	2184	2186	NOP56_GSE43666-GSM1067863.bed	315_CLIPdb_Piranha_NOP56.bed	HEK293
312	CLIPdb_PARalyzer	NOP58	23706177	Kishore, S. et al.	PAR-CLIP	4901	4902	NOP58_GSE43666-GSM1067861.bed	316_CLIPdb_PARalyzer_NOP58.bed	HEK293
313	CLIPdb_PARalyzer	NOP58	23706177	Kishore, S. et al.	PAR-CLIP	3414	3415	NOP58_GSE43666-GSM1067862.bed	317_CLIPdb_PARalyzer_NOP58.bed	HEK293
314	CLIPdb_Piranha	NOP58	23706177	Kishore, S. et al.	PAR-CLIP	2621	2623	NOP58_GSE43666-GSM1067861.bed	318_CLIPdb_Piranha_NOP58.bed	HEK293
315	CLIPdb_Piranha	NOP58	23706177	Kishore, S. et al.	PAR-CLIP	1099	1099	NOP58_GSE43666-GSM1067862.bed	319_CLIPdb_Piranha_NOP58.bed	HEK293
316	CLIPdb_PARalyzer	NUDT21	22813749	Martin, G., Gruber, A. R., Keller, W. & Zavolan, M.	PAR-CLIP	331633	331651	NUDT21_GSE37401-GSM917661.bed	320_CLIPdb_PARalyzer_NUDT21.bed	HEK293
317	CLIPdb_PARalyzer	NUDT21	22813749	Martin, G., Gruber, A. R., Keller, W. & Zavolan, M.	PAR-CLIP	16026	16033	NUDT21_GSE37401-GSM917662.bed	321_CLIPdb_PARalyzer_NUDT21.bed	HEK293
318	CLIPdb_Piranha	NUDT21	22813749	Martin, G., Gruber, A. R., Keller, W. & Zavolan, M.	PAR-CLIP	11387	11391	NUDT21_GSE37401-GSM917661.bed	322_CLIPdb_Piranha_NUDT21.bed	HEK293
319	CLIPdb_Piranha	NUDT21	22813749	Martin, G., Gruber, A. R., Keller, W. & Zavolan, M.	PAR-CLIP	23232	23240	NUDT21_GSE37401-GSM917662.bed	323_CLIPdb_Piranha_NUDT21.bed	HEK293
320	CLIPdb_Piranha	PTBP1	25219497	Blencowe BJ, et al.	iCLIP	40781	40792	PTBP1_GSE57278-GSM1486178.bed	326_CLIPdb_Piranha_PTBP1.bed	HEK293T
321	CLIPdb_Piranha	PTBP1	25219497	Blencowe BJ, et al.	iCLIP	74553	74564	PTBP1_GSE57278-GSM1486177.bed	327_CLIPdb_Piranha_PTBP1.bed	HEK293T
322	CLIPdb_Piranha	PTBP1	25219497	Blencowe BJ, et al.	PAR-iCLIP	82141	82158	PTBP1_GSE57278-GSM1378377.bed	328_CLIPdb_Piranha_PTBP1.bed	HEK293T
323	CLIPdb_CIMS	PTBP1/PTBP2	20064465	Xue, Y. et al.	HITS-CLIP	79	79	PTBP1/PTBP2_GSE19323-GSM480476.bed	329_CLIPdb_CIMS_PTBP1_PTBP2.bed	HeLa
324	CLIPdb_CIMS	PTBP1/PTBP2	20064465	Xue, Y. et al.	HITS-CLIP	109	109	PTBP1/PTBP2_GSE19323-GSM480477.bed	330_CLIPdb_CIMS_PTBP1_PTBP2.bed	HeLa
325	CLIPdb_CIMS	PTBP1/PTBP2	20064465	Xue, Y. et al.	HITS-CLIP	93	94	PTBP1/PTBP2_GSE19323-GSM480478.bed	331_CLIPdb_CIMS_PTBP1_PTBP2.bed	HeLa
326	CLIPdb_CIMS	PTBP1/PTBP2	20064465	Xue, Y. et al.	HITS-CLIP	288	288	PTBP1/PTBP2_GSE19323-GSM480479.bed	332_CLIPdb_CIMS_PTBP1_PTBP2.bed	HeLa
327	CLIPdb_CIMS	PTBP1/PTBP2	20064465	Xue, Y. et al.	HITS-CLIP	525	528	PTBP1/PTBP2_GSE42701-GSM1048186.bed	333_CLIPdb_CIMS_PTBP1_PTBP2.bed	HeLa
328	CLIPdb_Piranha	PTBP1/PTBP2	20064465	Xue, Y. et al.	HITS-CLIP	8356	8357	PTBP1/PTBP2_GSE19323-GSM480476.bed	334_CLIPdb_Piranha_PTBP1_PTBP2.bed	HeLa
329	CLIPdb_Piranha	PTBP1/PTBP2	20064465	Xue, Y. et al.	HITS-CLIP	10875	10876	PTBP1/PTBP2_GSE19323-GSM480477.bed	335_CLIPdb_Piranha_PTBP1_PTBP2.bed	HeLa
330	CLIPdb_Piranha	PTBP1/PTBP2	20064465	Xue, Y. et al.	HITS-CLIP	4706	4705	PTBP1/PTBP2_GSE19323-GSM480478.bed	336_CLIPdb_Piranha_PTBP1_PTBP2.bed	HeLa
331	CLIPdb_Piranha	PTBP1/PTBP2	20064465	Xue, Y. et al.	HITS-CLIP	13173	13175	PTBP1/PTBP2_GSE19323-GSM480479.bed	337_CLIPdb_Piranha_PTBP1_PTBP2.bed	HeLa
332	CLIPdb_Piranha	PTBP1/PTBP2	20064465	Xue, Y. et al.	HITS-CLIP	17133	17137	PTBP1/PTBP2_GSE42701-GSM1048186.bed	338_CLIPdb_Piranha_PTBP1_PTBP2.bed	HeLa
333	CLIPdb_PARalyzer	PUM2	20371350	Hafner, M. et al.	PAR-CLIP	6831	6831	PUM2_GSE21578-GSM545210.bed	339_CLIPdb_PARalyzer_PUM2.bed	HEK293
334	CLIPdb_Piranha	PUM2	20371350	Hafner, M. et al.	PAR-CLIP	1236	1236	PUM2_GSE21578-GSM545210.bed	340_CLIPdb_Piranha_PUM2.bed	HEK293
335	CLIPdb_PARalyzer	QKI	20371350	Hafner, M. et al.	PAR-CLIP	8565	8565	QKI_GSE21578-GSM545211.bed	341_CLIPdb_PARalyzer_QKI.bed	HEK293
336	CLIPdb_Piranha	QKI	20371350	Hafner, M. et al.	PAR-CLIP	1168	1169	QKI_GSE21578-GSM545211.bed	342_CLIPdb_Piranha_QKI.bed	HEK293

337	CLIPdb_PARalyzer	RTCB	22681889	Baltz, A. G. et al.	PAR-CLIP	31316	31318	RTCB_GSE38201-GSM936508.bed	343_CLIPdb_PARalyzer_RTCB.bed	HEK293
338	CLIPdb_Piranha	RTCB	22681889	Baltz, A. G. et al.	PAR-CLIP	3247	3250	RTCB_GSE38201-GSM936508.bed	344_CLIPdb_Piranha_RTCB.bed	HEK293
339	CLIPdb_Piranha	SRRM4	25219497	Blencowe BJ, et al.	iCLIP	368	369	SRRM4_GSE57278-GSM1486179.bed	346_CLIPdb_Piranha_SRRM4.bed	HEK293T
340	CLIPdb_Piranha	SRRM4	25219497	Blencowe BJ, et al.	PAR-iCLIP	11116	11128	SRRM4_GSE57278-GSM1378376.bed	347_CLIPdb_Piranha_SRRM4.bed	HEK293T
341	CLIPdb_Piranha	SRRM4	25219497	Blencowe BJ, et al.	PAR-iCLIP	2951	2955	SRRM4_GSE57278-GSM1378375.bed	348_CLIPdb_Piranha_SRRM4.bed	HEK293T
342	CLIPdb_Piranha	SRRM4	25219497	Blencowe BJ, et al.	PAR-iCLIP	29378	29394	SRRM4_GSE57278-GSM1378374.bed	349_CLIPdb_Piranha_SRRM4.bed	HEK293T
343	CLIPdb_CIMS	TAF15	23416048	Ibrahim, F. et al.	HITS-CLIP	43	43	TAF15_GSE43294-GSM1060205.bed	350_CLIPdb_CIMS_TAF15.bed	Brain
344	CLIPdb_CIMS	TAF15	23416048	Ibrahim, F. et al.	HITS-CLIP	167	167	TAF15_GSE43294-GSM1060206.bed	351_CLIPdb_CIMS_TAF15.bed	Brain
345	CLIPdb_CIMS	TAF15	23416048	Ibrahim, F. et al.	HITS-CLIP	170	170	TAF15_GSE43294-GSM1060207.bed	352_CLIPdb_CIMS_TAF15.bed	Brain
346	CLIPdb_CIMS	TAF15	23416048	Ibrahim, F. et al.	HITS-CLIP	105	105	TAF15_GSE43294-GSM1060208.bed	353_CLIPdb_CIMS_TAF15.bed	Brain
347	CLIPdb_CIMS	TAF15	23416048	Ibrahim, F. et al.	HITS-CLIP	13	13	TAF15_GSE43294-GSM1060209.bed	354_CLIPdb_CIMS_TAF15.bed	Brain
348	CLIPdb_PARalyzer	TAF15	22081015	Hoell, J. I. et al.	PAR-CLIP	8687	8687	TAF15_SRX029345.bed	355_CLIPdb_PARalyzer_TAF15.bed	HEK293
349	CLIPdb_PARalyzer	TAF15	22081015	Hoell, J. I. et al.	PAR-CLIP	15409	15409	TAF15_SRX029346-SRX029346.bed	356_CLIPdb_PARalyzer_TAF15.bed	HEK293
350	CLIPdb_Piranha	TAF15	23416048	Ibrahim, F. et al.	HITS-CLIP	566	566	TAF15_GSE43294-GSM1060205.bed	357_CLIPdb_Piranha_TAF15.bed	Brain
351	CLIPdb_Piranha	TAF15	23416048	Ibrahim, F. et al.	HITS-CLIP	799	799	TAF15_GSE43294-GSM1060206.bed	358_CLIPdb_Piranha_TAF15.bed	Brain
352	CLIPdb_Piranha	TAF15	23416048	Ibrahim, F. et al.	HITS-CLIP	1567	1567	TAF15_GSE43294-GSM1060207.bed	359_CLIPdb_Piranha_TAF15.bed	Brain
353	CLIPdb_Piranha	TAF15	23416048	Ibrahim, F. et al.	HITS-CLIP	878	878	TAF15_GSE43294-GSM1060208.bed	360_CLIPdb_Piranha_TAF15.bed	Brain
354	CLIPdb_Piranha	TAF15	23416048	Ibrahim, F. et al.	HITS-CLIP	372	372	TAF15_GSE43294-GSM1060209.bed	361_CLIPdb_Piranha_TAF15.bed	Brain
355	CLIPdb_Piranha	TAF15	22081015	Hoell, J. I. et al.	PAR-CLIP	5778	5778	TAF15_SRX029345.bed	362_CLIPdb_Piranha_TAF15.bed	HEK293
356	CLIPdb_Piranha	TAF15	22081015	Hoell, J. I. et al.	PAR-CLIP	5791	5792	TAF15_SRX029346.bed	363_CLIPdb_Piranha_TAF15.bed	HEK293
357	CLIPdb_CIMS	TARDBP	21358640	Tollervey, J. R. et al.	iCLIP	399	399	TARDBP_E-MTAB-530-ERR039842.bed	364_CLIPdb_CIMS_TARDBP.bed	Brain
358	CLIPdb_CIMS	TARDBP	21358640	Tollervey, J. R. et al.	iCLIP	2441	2442	TARDBP_E-MTAB-530-ERR039843.bed	365_CLIPdb_CIMS_TARDBP.bed	Brain
359	CLIPdb_CIMS	TARDBP	21358640	Tollervey, J. R. et al.	iCLIP	717	717	TARDBP_E-MTAB-530-ERR039844.bed	366_CLIPdb_CIMS_TARDBP.bed	Brain
360	CLIPdb_CIMS	TARDBP	21358640	Tollervey, J. R. et al.	iCLIP	1118	1119	TARDBP_E-MTAB-530-ERR039845.bed	367_CLIPdb_CIMS_TARDBP.bed	Brain
361	CLIPdb_CIMS	TARDBP	21358640	Tollervey, J. R. et al.	iCLIP	772	773	TARDBP_E-MTAB-530-ERR039846.bed	368_CLIPdb_CIMS_TARDBP.bed	Brain
362	CLIPdb_CIMS	TARDBP	21358640	Tollervey, J. R. et al.	iCLIP	729	729	TARDBP_E-MTAB-530-ERR039847-ERR039848.bed	369_CLIPdb_CIMS_TARDBP.bed	Brain
363	CLIPdb_CIMS	TARDBP	21358640	Tollervey, J. R. et al.	iCLIP	108620	108651	TARDBP_E-MTAB-530-ERR039849.bed	370_CLIPdb_CIMS_TARDBP.bed	Brain
364	CLIPdb_CIMS	TARDBP	21358640	Tollervey, J. R. et al.	iCLIP	437	437	TARDBP_E-MTAB-530-ERR039850.bed	371_CLIPdb_CIMS_TARDBP.bed	H9
365	CLIPdb_CIMS	TARDBP	21358640	Tollervey, J. R. et al.	iCLIP	3599	3600	TARDBP_E-MTAB-530-ERR039851-ERR039852.bed	372_CLIPdb_CIMS_TARDBP.bed	SH_SY5Y
366	CLIPdb_CIMS	TARDBP	21358640	Tollervey, J. R. et al.	iCLIP	75	75	TARDBP_E-MTAB-530-ERR039853.bed	373_CLIPdb_CIMS_TARDBP.bed	SH_SY5Y
367	CLIPdb_CIMS	TARDBP	21358640	Tollervey, J. R. et al.	iCLIP	1698	1698	TARDBP_E-MTAB-530-ERR039854.bed	374_CLIPdb_CIMS_TARDBP.bed	SH_SY5Y
368	CLIPdb_CIMS	TARDBP	21358640	Tollervey, J. R. et al.	iCLIP	39625	39637	TARDBP_E-MTAB-530-ERR039855.bed	375_CLIPdb_CIMS_TARDBP.bed	SH_SY5Y
369	CLIPdb_CITS	TARDBP	21358640	Tollervey, J. R. et al.	iCLIP	399	399	TARDBP_E-MTAB-530-ERR039842.bed	376_CLIPdb_CITS_TARDBP.bed	Brain
370	CLIPdb_CITS	TARDBP	21358640	Tollervey, J. R. et al.	iCLIP	2441	2442	TARDBP_E-MTAB-530-ERR039843.bed	377_CLIPdb_CITS_TARDBP.bed	Brain
371	CLIPdb_CITS	TARDBP	21358640	Tollervey, J. R. et al.	iCLIP	717	717	TARDBP_E-MTAB-530-ERR039844.bed	378_CLIPdb_CITS_TARDBP.bed	Brain
372	CLIPdb_CITS	TARDBP	21358640	Tollervey, J. R. et al.	iCLIP	1118	1119	TARDBP_E-MTAB-530-ERR039845.bed	379_CLIPdb_CITS_TARDBP.bed	Brain
373	CLIPdb_CITS	TARDBP	21358640	Tollervey, J. R. et al.	iCLIP	772	773	TARDBP_E-MTAB-530-ERR039846.bed	380_CLIPdb_CITS_TARDBP.bed	Brain
374	CLIPdb_CITS	TARDBP	21358640	Tollervey, J. R. et al.	iCLIP	729	729	TARDBP_E-MTAB-530-ERR039847-ERR039848.bed	381_CLIPdb_CITS_TARDBP.bed	Brain
375	CLIPdb_CITS	TARDBP	21358640	Tollervey, J. R. et al.	iCLIP	108620	108651	TARDBP_E-MTAB-530-ERR039849.bed	382_CLIPdb_CITS_TARDBP.bed	Brain
376	CLIPdb_CITS	TARDBP	21358640	Tollervey, J. R. et al.	iCLIP	437	437	TARDBP_E-MTAB-530-ERR039850.bed	383_CLIPdb_CITS_TARDBP.bed	H9
377	CLIPdb_CITS	TARDBP	21358640	Tollervey, J. R. et al.	iCLIP	3599	3600	TARDBP_E-MTAB-530-ERR039851-ERR039852.bed	384_CLIPdb_CITS_TARDBP.bed	SH_SY5Y
378	CLIPdb_CITS	TARDBP	21358640	Tollervey, J. R. et al.	iCLIP	75	75	TARDBP_E-MTAB-530-ERR039853.bed	385_CLIPdb_CITS_TARDBP.bed	SH_SY5Y
379	CLIPdb_CITS	TARDBP	21358640	Tollervey, J. R. et al.	iCLIP	1698	1698	TARDBP_E-MTAB-530-ERR039854.bed	386_CLIPdb_CITS_TARDBP.bed	SH_SY5Y

380	CLIPdb_CITS	TARDBP	21358640	Tollervey, J. R. et al.	iCLIP	39625	39637	TARDBP_E-MTAB-530-ERR039855.bed	387_CLIPdb_CITS_TARDBP.bed	SH_SY5Y
381	CLIPdb_PARalyzer	TARDBP	24954906	Kawahara, Y. et al.	PAR-CLIP	69393	69456	TARDBP_DRA001158-DRS012385.bed	388_CLIPdb_PARalyzer_TARDBP.bed	HEK293T
382	CLIPdb_PARalyzer	TARDBP	24954906	Kawahara, Y. et al.	PAR-CLIP	511590	511687	TARDBP_DRA001158-DRS012386.bed	389_CLIPdb_PARalyzer_TARDBP.bed	HEK293T
383	CLIPdb_PARalyzer	TARDBP	24954906	Kawahara, Y. et al.	PAR-CLIP	268144	268228	TARDBP_DRA001158-DRS012387.bed	390_CLIPdb_PARalyzer_TARDBP.bed	HEK293T
384	CLIPdb_Piranha	TARDBP	21358640	Tollervey, J. R. et al.	iCLIP	33	33	TARDBP_E-MTAB-530-ERR039842.bed	391_CLIPdb_Piranha_TARDBP.bed	Brain
385	CLIPdb_Piranha	TARDBP	21358640	Tollervey, J. R. et al.	iCLIP	668	670	TARDBP_E-MTAB-530-ERR039843.bed	392_CLIPdb_Piranha_TARDBP.bed	Brain
386	CLIPdb_Piranha	TARDBP	21358640	Tollervey, J. R. et al.	iCLIP	322	323	TARDBP_E-MTAB-530-ERR039844.bed	393_CLIPdb_Piranha_TARDBP.bed	Brain
387	CLIPdb_Piranha	TARDBP	21358640	Tollervey, J. R. et al.	iCLIP	658	659	TARDBP_E-MTAB-530-ERR039845.bed	394_CLIPdb_Piranha_TARDBP.bed	Brain
388	CLIPdb_Piranha	TARDBP	21358640	Tollervey, J. R. et al.	iCLIP	617	618	TARDBP_E-MTAB-530-ERR039846.bed	395_CLIPdb_Piranha_TARDBP.bed	Brain
389	CLIPdb_Piranha	TARDBP	21358640	Tollervey, J. R. et al.	iCLIP	1578	1579	TARDBP_E-MTAB-530-ERR039847-ERR039848.bed	396_CLIPdb_Piranha_TARDBP.bed	Brain
390	CLIPdb_Piranha	TARDBP	21358640	Tollervey, J. R. et al.	iCLIP	16541	16547	TARDBP_E-MTAB-530-ERR039849.bed	397_CLIPdb_Piranha_TARDBP.bed	Brain
391	CLIPdb_Piranha	TARDBP	21358640	Tollervey, J. R. et al.	iCLIP	123	123	TARDBP_E-MTAB-530-ERR039850.bed	398_CLIPdb_Piranha_TARDBP.bed	H9
392	CLIPdb_Piranha	TARDBP	24954906	Kawahara, Y. et al.	PAR-CLIP	2280	2283	TARDBP_DRA001158-DRS012385.bed	399_CLIPdb_Piranha_TARDBP.bed	HEK293T
393	CLIPdb_Piranha	TARDBP	24954906	Kawahara, Y. et al.	PAR-CLIP	7595	7601	TARDBP_DRA001158-DRS012386.bed	400_CLIPdb_Piranha_TARDBP.bed	HEK293T
394	CLIPdb_Piranha	TARDBP	24954906	Kawahara, Y. et al.	PAR-CLIP	3904	3904	TARDBP_DRA001158-DRS012387.bed	401_CLIPdb_Piranha_TARDBP.bed	HEK293T
395	CLIPdb_Piranha	TARDBP	21358640	Tollervey, J. R. et al.	iCLIP	2734	2736	TARDBP_E-MTAB-530-ERR039851-ERR039852.bed	402_CLIPdb_Piranha_TARDBP.bed	SH_SY5Y
396	CLIPdb_Piranha	TARDBP	21358640	Tollervey, J. R. et al.	iCLIP	86	86	TARDBP_E-MTAB-530-ERR039853.bed	403_CLIPdb_Piranha_TARDBP.bed	SH_SY5Y
397	CLIPdb_Piranha	TARDBP	21358640	Tollervey, J. R. et al.	iCLIP	1092	1092	TARDBP_E-MTAB-530-ERR039854.bed	404_CLIPdb_Piranha_TARDBP.bed	SH_SY5Y
398	CLIPdb_Piranha	TARDBP	21358640	Tollervey, J. R. et al.	iCLIP	8103	8107	TARDBP_E-MTAB-530-ERR039855.bed	405_CLIPdb_Piranha_TARDBP.bed	SH_SY5Y
399	CLIPdb_CIMS	TIA1	21048981	Wang, Z. et al.	iCLIP	7908	7907	TIA1_E-MTAB-432-ERR039774-ERR039775-ERR039777.bed	406_CLIPdb_CIMS_TIA1.bed	HeLa
400	CLIPdb_CIMS	TIA1	21048981	Wang, Z. et al.	iCLIP	5320	5317	TIA1_E-MTAB-432-ERR039776-ERR039778.bed	407_CLIPdb_CIMS_TIA1.bed	HeLa
401	CLIPdb_CIMS	TIA1	21048981	Wang, Z. et al.	iCLIP	9811	9816	TIA1_E-MTAB-432-ERR039786-ERR039787.bed	408_CLIPdb_CIMS_TIA1.bed	HeLa
402	CLIPdb_CITS	TIA1	21048981	Wang, Z. et al.	iCLIP	7908	7907	TIA1_E-MTAB-432-ERR039774-ERR039775-ERR039777.bed	409_CLIPdb_CITS_TIA1.bed	HeLa
403	CLIPdb_CITS	TIA1	21048981	Wang, Z. et al.	iCLIP	5320	5317	TIA1_E-MTAB-432-ERR039776-ERR039778.bed	410_CLIPdb_CITS_TIA1.bed	HeLa
404	CLIPdb_CITS	TIA1	21048981	Wang, Z. et al.	iCLIP	9811	9816	TIA1_E-MTAB-432-ERR039786-ERR039787.bed	411_CLIPdb_CITS_TIA1.bed	HeLa
405	CLIPdb_Piranha	TIA1	21048981	Wang, Z. et al.	iCLIP	2388	2386	TIA1_E-MTAB-432-ERR039774-ERR039775-ERR039777.bed	412_CLIPdb_Piranha_TIA1.bed	HeLa
406	CLIPdb_Piranha	TIA1	21048981	Wang, Z. et al.	iCLIP	2014	2014	TIA1_E-MTAB-432-ERR039776-ERR039778.bed	413_CLIPdb_Piranha_TIA1.bed	HeLa
407	CLIPdb_Piranha	TIA1	21048981	Wang, Z. et al.	iCLIP	4188	4189	TIA1_E-MTAB-432-ERR039786-ERR039787.bed	414_CLIPdb_Piranha_TIA1.bed	HeLa
408	CLIPdb_PARalyzer	TNRC6A	20371350	Hafner, M. et al.	PAR-CLIP	969	969	TNRC6A_GSE21578-GSM545218.bed	415_CLIPdb_PARalyzer_TNRC6A.bed	HEK293
409	CLIPdb_Piranha	TNRC6A	20371350	Hafner, M. et al.	PAR-CLIP	659	659	TNRC6A_GSE21578-GSM545218.bed	416_CLIPdb_Piranha_TNRC6A.bed	HEK293
410	CLIPdb_PARalyzer	TNRC6B	20371350	Hafner, M. et al.	PAR-CLIP	744	744	TNRC6B_GSE21578-GSM545219.bed	417_CLIPdb_PARalyzer_TNRC6B.bed	HEK293
411	CLIPdb_Piranha	TNRC6B	20371350	Hafner, M. et al.	PAR-CLIP	367	367	TNRC6B_GSE21578-GSM545219.bed	418_CLIPdb_Piranha_TNRC6B.bed	HEK293
412	CLIPdb_PARalyzer	TNRC6C	20371350	Hafner, M. et al.	PAR-CLIP	664	664	TNRC6C_GSE21578-GSM545220.bed	419_CLIPdb_PARalyzer_TNRC6C.bed	HEK293
413	CLIPdb_Piranha	TNRC6C	20371350	Hafner, M. et al.	PAR-CLIP	586	586	TNRC6C_GSE21578-GSM545220.bed	420_CLIPdb_Piranha_TNRC6C.bed	HEK293
414	CLIPdb_PARalyzer	WDR33	25301781	Wahle E et al.	PAR-CLIP	8467	8470	WDR33_GSE61123-GSM1496985.bed	421_CLIPdb_PARalyzer_WDR33.bed	HEK293
415	CLIPdb_Piranha	WDR33	25301781	Wahle E et al.	PAR-CLIP	6065	6067	WDR33_GSE61123-GSM1496985.bed	422_CLIPdb_Piranha_WDR33.bed	HEK293

416	CLIPdb_PARalyzer	YTHDF2	24284625	Wang, X. et al.	PAR-CLIP	10613	10614	YTHDF2_GSE49339-GSM1197605.bed	423_CLIPdb_PARalyzer_YTHDF2.bed	HeLa
417	CLIPdb_PARalyzer	YTHDF2	24284625	Wang, X. et al.	PAR-CLIP	18210	18214	YTHDF2_GSE49339-GSM1197606.bed	424_CLIPdb_PARalyzer_YTHDF2.bed	HeLa
418	CLIPdb_PARalyzer	YTHDF2	24284625	Wang, X. et al.	PAR-CLIP	36382	36388	YTHDF2_GSE49339-GSM1197607.bed	425_CLIPdb_PARalyzer_YTHDF2.bed	HeLa
419	CLIPdb_Piranha	YTHDF2	24284625	Wang, X. et al.	PAR-CLIP	5378	5379	YTHDF2_GSE49339-GSM1197605.bed	426_CLIPdb_Piranha_YTHDF2.bed	HeLa
420	CLIPdb_Piranha	YTHDF2	24284625	Wang, X. et al.	PAR-CLIP	4525	4529	YTHDF2_GSE49339-GSM1197606.bed	427_CLIPdb_Piranha_YTHDF2.bed	HeLa
421	CLIPdb_Piranha	YTHDF2	24284625	Wang, X. et al.	PAR-CLIP	4476	4478	YTHDF2_GSE49339-GSM1197607.bed	428_CLIPdb_Piranha_YTHDF2.bed	HeLa
422	CLIPdb_PARalyzer	ZC3H7B	22681889	Baltz, A. G. et al.	PAR-CLIP	190909	190927	ZC3H7B_GSE38201-GSM936510.bed	429_CLIPdb_PARalyzer_ZC3H7B.bed	HEK293
423	CLIPdb_Piranha	ZC3H7B	22681889	Baltz, A. G. et al.	PAR-CLIP	20869	20872	ZC3H7B_GSE38201-GSM936510.bed	430_CLIPdb_Piranha_ZC3H7B.bed	HEK293
